# Integrating Biological Early Warning Systems with High-Resolution Online Chemical Monitoring in Wastewater Treatment Plants

**DOI:** 10.1021/acs.est.4c07316

**Published:** 2024-12-18

**Authors:** Ali Kizgin, Danina Schmidt, Julian Bosshard, Heinz Singer, Juliane Hollender, Eberhard Morgenroth, Cornelia Kienle, Miriam Langer

**Affiliations:** † Swiss Centre for Applied Ecotoxicology, Dübendorf, 8600 Zürich, Switzerland; ‡ Eawag, Swiss Federal Institute of Aquatic Science and Technology, 8647 Kastanienbaum, Switzerland; § University of Tübingen, Animal Physiological Ecology, 72074 Tübingen, Germany; ∥ Eawag, Swiss Federal Institute of Aquatic Science and Technology, Dübendorf, 8600 Zürich, Switzerland; ⊥ Institute of Biogeochemistry and Pollutant Dynamics, ETH Zürich, 8092 Zürich, Switzerland; # Institute of Environmental Engineering, ETH Zürich, 8092 Zürich, Switzerland; ∇ Institute for Ecopreneurship, FHNW Muttenz, 4132 Muttenz, Switzerland

**Keywords:** biological early warning systems, in situ high resolution mass spectrometry, monitoring, wastewater, effluent

## Abstract

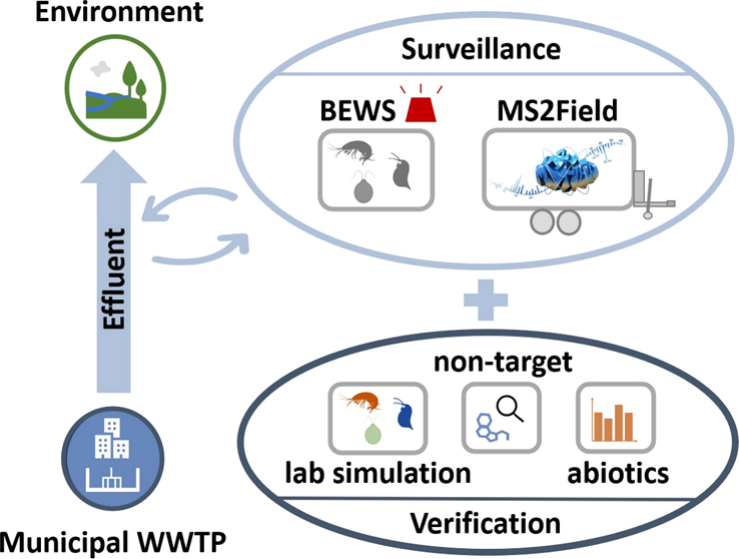

Detection of micropollutants (MPs) in wastewater effluents using traditional toxicity tests or chemical analysis with discrete samples is challenging due to concentration dynamics. This study evaluates a continuous monitoring approach for detecting MPs in wastewater effluents using a combination of biological early warning systems (BEWS). Three BEWS with *Chlorella vulgaris*, *Daphnia magna*, and *Gammarus pulex* were operated in parallel in a full-scale municipal wastewater treatment plant. Concentrations of MPs were monitored by simultaneous online chemical analysis using high performance liquid chromatography-high resolution mass spectrometry (MS2Field). Over 5 weeks, behavioral changes observed in the BEWS occasionally exceeded acute toxicity thresholds, triggering alarms. These changes were related to MPs identified by the MS2Field, to abiotic factors, or to operational parameters of the BEWS. For one toxic event, behavioral responses were linked to a pesticide, not authorized in Switzerland, at concentrations close to literature EC_50_ values. Verification tests confirmed that the pesticide in the effluent was the most likely cause for the organism response. The study demonstrates the potential of BEWS as a stand-alone technique for detecting contamination peaks in wastewater, and identifies key limitations and critical factors that need to be addressed to optimize the use of BEWS in wastewater monitoring.

## Abstract 2

Biological Early Warning Systems allow real-time assessment of wastewater quality and in combination with (online) chemical analysis identification of toxic compounds.

## Introduction

Wastewater treatment plants (WWTPs) remain a major point source of surface water pollution.^1,2^ Municipal WWTPs, receiving both domestic and industrial discharges, must cope with irregular peak emissions of micropollutants (MPs).^3^ The highly variable load of MPs from industrial production sites and agriculture challenges regular wastewater monitoring approaches and calls for improved monitoring techniques to effectively trace MPs back to their origin.^4^ The revision of the European zero pollution action plan under the European Green Deal in 2022 marks a significant advancement in wastewater management strategies. The revised plan emphasizes not only the effective removal or reduction of MPs to levels where their environmental impact is negligible, but also underscores the critical importance of source management.^5^ Current research, such as that of the Environmental Protection Agency (EPA), points to the development and evaluation of analytical methods, including nontargeted analytical techniques combined with bioassays, for the detection of a wide range of MPs and emerging contaminants in WWTP effluent.^6^ These advanced methods go beyond traditional approaches, which are mostly based on time-limited analyses covering various wastewater sampling techniques, chemical analyses, and, rarely, bioanalytical screening tools.^7^

While the costs and time associated with effluent monitoring, from sampling to laboratory chemical screening and bioassays, can be significant, the high spatial and temporal dynamics of MPs in wastewater pose an additional challenge. Key issues are (i) the composition of the WWTP influent can vary significantly within hours and days due to changing industrial production cycles,^8^ (ii) weather conditions, such as heavy rainfall, can overwhelm sewers^9^ and (iii) treatment plants, and temperature variations can affect treatment efficiency.^10^ These dynamics cannot be adequately reflected in grab or composite samples, but should be considered in the interpretation and management of wastewater quality. Continuous, cost-effective, and simultaneously high-resolution monitoring approaches could capture these dynamics. These include biological early warning systems (BEWS), which use living aquatic organisms to continuously monitor water quality and provide rapid warnings of emerging chemical hazards.^11^ Several decades ago, the applicability of BEWS for wastewater assessment was tested and sidelined due to practical challenges such as clogging, insufficient sensitivity, or incompatibility of organisms with the wastewater matrix leading to false alarms.^12^ Recent technological advancements and enhanced data analysis capabilities have helped streamline some aspects of using BEWS in wastewater monitoring.^13−15^ Our previous study tested BEWS with organisms of different trophic levels in prefiltered effluent from a pilot WWTP, demonstrating reliability and providing valuable data to advance the adoption of BEWS for effluent monitoring.^16^ Nonetheless, the significance of BEWS responses and alarms need to be proven before BEWS can be integrated into the wastewater sector and accepted for wastewater monitoring practices. Comprehensive studies including the identification of toxic compounds triggered by BEWS signals are required to ensure the reliability of the BEWS in practice. Schymanski et al.^17^ emphasized the necessity of nontarget chemical analysis, besides target screening, to identify MPs that may be present but remain untraceable using traditional methods. Neale et al.^18^ and Kienle et al.,^1^ who evaluated how much of the effect measured in bioassays could be explained by targeted chemical analysis of samples from up to 24 WWTPs and adjacent streams, noted the limitations of using target analysis alone. Accordingly, Anliker et al.^19^ demonstrated the effectiveness of a nontarget approach in detecting industrial emissions in wastewater streams, and Köke et al.^20^ showed the potential of an online monitoring setup to investigate the dynamics of MPs in WWTP effluent. These studies, together with our current research, demonstrate the critical need for integrating BEWS and online target and nontarget chemical monitoring in wastewater contexts, helping to close the existing gap in long-term, high-resolution monitoring strategies. The development of the transportable automated liquid chromatography-high resolution mass spectrometry (LC-HRMS/MS) platform, MS2Field,^21^ allows such monitoring with high resolution over long periods of time. It has recently been demonstrated that the MS2Field can be used to successfully track the presence of active pharmaceutical ingredients in treated wastewater.^22^ However, attributing chemical occurrence to behavioral responses is challenging due to potential false positive alarms caused by other parameters. Therefore, concurrent monitoring of physicochemical (e.g., nitrite, nitrate, ammonia) and abiotic parameters (e.g., temperature, pH, conductivity) of the wastewater should be included in the evaluation as highlighted by Van der Schalie et al.^23^ In this study, a battery of three BEWS using three different organisms targeting sublethal effects was coupled to the MS2Field to investigate the relationship between BEWS alarms and the occurrence of chemicals in the effluent of a municipal WWTP. The BEWS provided continuous monitoring of wastewater for biological responses such as behavior changes, while the full scan data of the MS2Field were used to identify compounds to which the organisms in the BEWS had responded. The objectives of this study were: (i) to detect BEWS signals occurring during effluent measurements at a municipal WWTP and, (ii) to check whether they can be attributed to the presence of MPs identified by MS2Field and verified by laboratory experiments or (iii) to other parameters. The results provide important insights that should guide improvements of BEWS for future applications in WWTPs.

## Materials and Methods

### Wastewater Treatment Plants

This study was conducted at a full-scale WWTP (Canton St. Gallen, Switzerland) that receives municipal wastewater from about 39 000 population equivalents (3.6 million m^3^/a). The treatment plant is equipped with activated sludge treatment including nitrification and denitrification as well as phosphate precipitation. The verification experiments were conducted at a pilot-scale WWTP (Eawag, Switzerland) receiving municipal wastewater and treating approximately 200 population equivalents (25 000 m^3^/a inflow). The mechanically treated wastewater passes through a sedimentation stage, followed by activated sludge treatment with denitrification and nitrification. Wastewater was transferred directly from the secondary clarifier to both the BEWS and the MS2Field systems.

### Biological Early Warning Systems

BEWS were originally developed for surface or drinking water monitoring—the use in wastewater presents new technical challenges such as adapting to a new water matrix, ensuring continuous maintenance-free measurements. Ultrafiltration (0.1 μm) was used to prevent biofouling and minimizing cleaning intervals for continuous operation. In addition, it helped to reduce background interference from bacteria and particulates that could affect BEWS organisms and/or detection systems and potentially cause false positives. This allowed the focus to be on dissolved chemical contaminants, such as micropollutants, improving the reliable detection of chemical toxicity. The ultrafiltration system was connected to the secondary clarifier of the WWTP to distribute treated wastewater continuously to each BEWS. The applied BEWS were (1) the Algae Toximeter (bbe Moldaenke), equipped with a photobioreactor that automatically cultivates the microalgae *Chlorella vulgaris* and exposes it to the water to be analyzed. The measurement of photosynthetic activity, which is compared to a control, serves as an indicator for the presence of toxic compounds.^24^ (2) the DaphTox II (bbe Moldaenke), which uses video tracking of the behavior of the water flea *Daphnia magna* to monitor changes in water quality,^25^ and (3) the Sensaguard (REMONDIS Aqua), which measures the locomotor behavior of *Gammarus pulex* in individual test chambers.^26^ The test chambers are placed in the monitored water and are equipped with sensors that detect the impedance generated by the organisms’ movement in an electromagnetic field. Maintenance of the devices was performed once a week including replacement of the test organisms. Additional information on organisms, BEWS and the filtration system can be found in Section S1–S6 in the Supporting information (SI).

### Evaluation of BEWS Reactions

**Figure 1:**
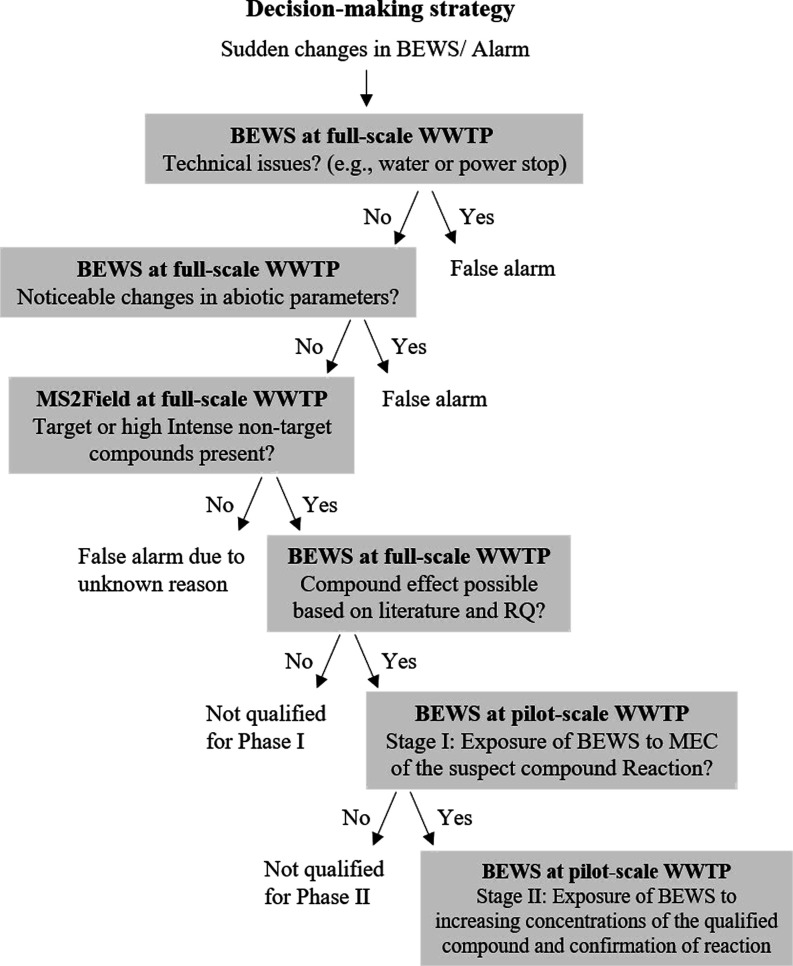
Strategy to evaluate BEWS responses for causative micropollutants.

Using BEWS for wastewater monitoring requires defined data evaluation criteria to interpret biological responses correctly. The alarm systems of the commercial BEWS provide an immediate indication of potential chemical hazards or significant disturbances in water quality and are triggered when there is a notable deviation from normal organism behavior. However, visible changes in organism behavior that do not trigger alarms may indicate subtle shifts in movement, metabolism, or growth patterns that may not exceed the alarm threshold but may still be indicative of underlying environmental disturbances, as shown by Kizgin et al.^16^ In our study, we evaluated both alarms and visible changes in behavior without alarms using our own more sensitive data evaluation methods. To enhance the reliability of BEWS data and improve the accuracy of wastewater quality assessment, a decision-making strategy was developed to take this into account (Figure 1). To identify potential causes of behavioral changes without alarms, sudden changes in behavior were treated in the same way as alarms. Identification of potential causes of abnormal behavior and alarms followed the strategy depicted in Figure 1. First, technical aspects such as water supply interruption were evaluated. Second, the potential influence of abiotic parameters, such as ammonium levels in wastewater, was considered. Third, the search for causative compounds was initiated through target and nontarget screening (NTS) of high intense signals. When elevated concentrations of compounds were detected, corresponding toxicity data from the literature were screened to assess if a potential contribution to the observed response was likely. If several suspect compounds were present simultaneously, a risk quotient (RQ) was calculated showing a ratio of the measured environmental concentration (MEC) and the effect concentration (EC_50_) derived from the literature. With this approach the most likely causative compounds for confirmation with laboratory experiments were prioritized. In stage I of the laboratory experiments conducted at the pilot WWTP, the selected compounds were added to the effluent at the concentration previously measured in the effluent of the full-scale WWTP and subsequently evaluated with the BEWS. If the organisms in the BEWS responded, they were exposed to the compounds again in stage II, this time at five increasing concentrations to identify reaction patterns and benchmark effect thresholds.

### Monitoring of Abiotic Parameters

Water quality parameters such as conductivity, temperature, pH and oxygen of the treated wastewater were measured continuously (every 5 min) with a Multi 3320 (WTW, Germany). The daily values for nitrite, nitrate and ammonium were obtained from the WWTP. Additional information on monitoring parameters can be found in Table X1 in Excel-SI.

### Chemical Data Analysis with MS2Field

The MS2Field was used in parallel with the BEWS for online chemical analysis. It is a transportable trailer with a fully automated LC-HRMS/MS platform that can autonomously sample and measure water samples with high temporal resolution.^21^ After filtration (<3–5 μm) through a cross-flow filter, a wastewater sample was collected every 20 min using a PAL Robotic Tool Charge autosampler (CTC Analytics, Switzerland). For the measurement, a diluter syringe was filled with the wastewater sample, standard (STD), isotopically labeled internal standards (ILIS) and nanopure/Evianwater (80:20). To remove small particles, the sample was directed through a self-packed precolumn (stainless steel, 2.1 mm × 20 mm, BGB Analytik AG, Switzerland) containing Atlantis T3 material (10 μm, Waters, Ireland). Then, analytes were separated on a reverse phase analytical column (Atlantis T3 5 μm, 3.0 mm × 50 mm, Waters, Ireland). The sample was transferred into the LC-HRMS/MS system by large volume direct injection and was eluted using a gradient of ultrapure water and methanol (both acidified with 0.1% formic acid). LC-HRMS/MS data was acquired on a hybrid quadrupole-orbitrap mass spectrometer (Q-Exactive HF, Thermo Scientific) with an ESI source. Technical and analytical details on the MS2Field are provided in S14–S16 in SI.

### Quantification of Target Substances and Method Validation

Target quantification in MS2Field samples was performed using an internal standard calibration method in TraceFinder 5.1^27^ (Thermo Fisher Scientific). Calibration curves were generated in ultrapure water using a single external STD for all compounds, and compounds were considered quantifiable if the relative recovery was between 75 and 125% and the relative standard deviation of triplicates was below 20%.

Matrix limits of quantification (LOQs) were calculated as the lowest detectable standard in ultrapure water, corrected by a matrix factor, respectively. Concentrations of compounds without a matching STD were adjusted based on relative recovery in spiked wastewater samples. Details on the quantification method of the targets are provided in S19 in SI.

### Non-Target Screening

If abiotic parameters and target compounds could be ruled out as the cause for behavioral responses, compounds present with high intensities in the effluent 48 h before, during, and 48 h after the behavioral response were identified using nontarget screening (NTS). Compound Discoverer (V3.3) software was used with the following filtering criteria: retention time between 1 and 20 min, blank subtraction as well as a minimum area of 1.0 × 10^8^. The structures of the filtered nontarget compounds including carbofuran, 2,4-dichlorophenol, and tributyl phosphate were matched and confirmed utilizing spectral libraries, including mzCloud^28^ and MassBank.^29^ Predicted molecular formula were derived based on the accurate mass of the molecular ions and the isotope pattern with the database ChemSpider.^30^ Reference standards for all three compounds confirmed level-1 identification according to Schymanski et al.^17^ The concentrations of the nontarget compounds were estimated using ILIS. To determine the response ratios between the ILIS and the nontarget compounds, calibration curves for the nontarget compounds were generated in ultrapure water from purchased standards. The peak intensities of the nontarget compounds in the samples were then compared to these calibration curves to determine their concentrations. It should be noted that matrix effects were not fully addressed, as calibration was conducted with ultrapure water rather than a wastewater matrix. To partially compensate for these matrix effects, an ILIS with a retention time similar to each nontarget compound was selected. The selection of exposure concentrations for laboratory verification was based on ecotoxicological criteria (EC_50_ values and mode of action). Details on the nontarget procedure are provided in S20 and S21 in SI.

### Verification Experiments

Laboratory BEWS experiments were conducted at Eawag’s pilot WWTP with substances that had been selected based on calculated RQs. Two stages of experiments were conducted: In the first stage, all selected substances were spiked individually or as mixture (according to the WWTP scenario) into the wastewater effluent. The first stage setup consisted of four phases: organism introduction (1 day), acclimation (2 day), exposure (24 h), and recovery phase (2 day). All phases were conducted under flow-through conditions, except for the exposure phase, where spiked wastewater was recirculated within the closed systems. In the second stage, the substance to which the organisms had responded in the first stage was added in increasing concentration. After a two-day acclimatization period, five concentration steps were applied with an exposure time of 24 h per concentration. For the exposure phase, a 100 L substance pool was prepared with filtered wastewater and the appropriate amount of compound. The stock solution was added to the wastewater pool 1 h before exposure and mixed using an aquarium pump (CompactOn 5000, EHEIM, Germany). Samples to determine analytical concentrations in the spiked wastewater pool were taken 1 and 20 h after the start of the exposure. The spiked pool was aerated (APS 300, USA) and cooled to 17 °C (Ultra Titan 150, Hailea, China). For details on stock preparation and final analytical concentrations see S22 in SI.

### Data Analysis and Statistics

All data were analyzed and visualized using the statistical software R, version 4.3.2 for Windows.^31^ In the case of DaphTox II and Algae Toximeter, the statistical data analysis has already been integrated into the algorithms of the systems. Therefore, no additional statistical tests were performed on these behavioral data. Statistical analysis of the Sensaguard data focused on changes in the behavioral patterns for the eight individual gammarids. For visualization, a multivariate changepoint detection based on Pickering (2015)^32^ using the “SMOB” package^33^ in R was applied and accompanied by a SIMQUANT analysis to evaluate the verification test, which is a linear random effects model to assess the daily rhythms of the gammarids.

A Generalized Linear Mixed Model (GLMM) was used in our analysis to investigate the relationship between chemical data or abiotic parameters and organism behavior over time, using the “glmmTMB” package in R.^34^ The model for the analysis considered behavioral data as the response variable, chemical and abiotic data as predictor variables, and individual differences between organisms and the temporal correlation of measurements taken on the same organism over time as random variation. To test the assumptions of the model and to ensure that they were met, we used a Type II analysis of variance, analogous to ANOVA for generalized linear models, to test the null hypothesis that each predictor has no effect on the response variable. This analysis was performed using Wald chi-squared tests. For more details on statistical evaluation see S11–S13 in SI.

## Results and Discussion

### BEWS Responses

**Figure 2:**
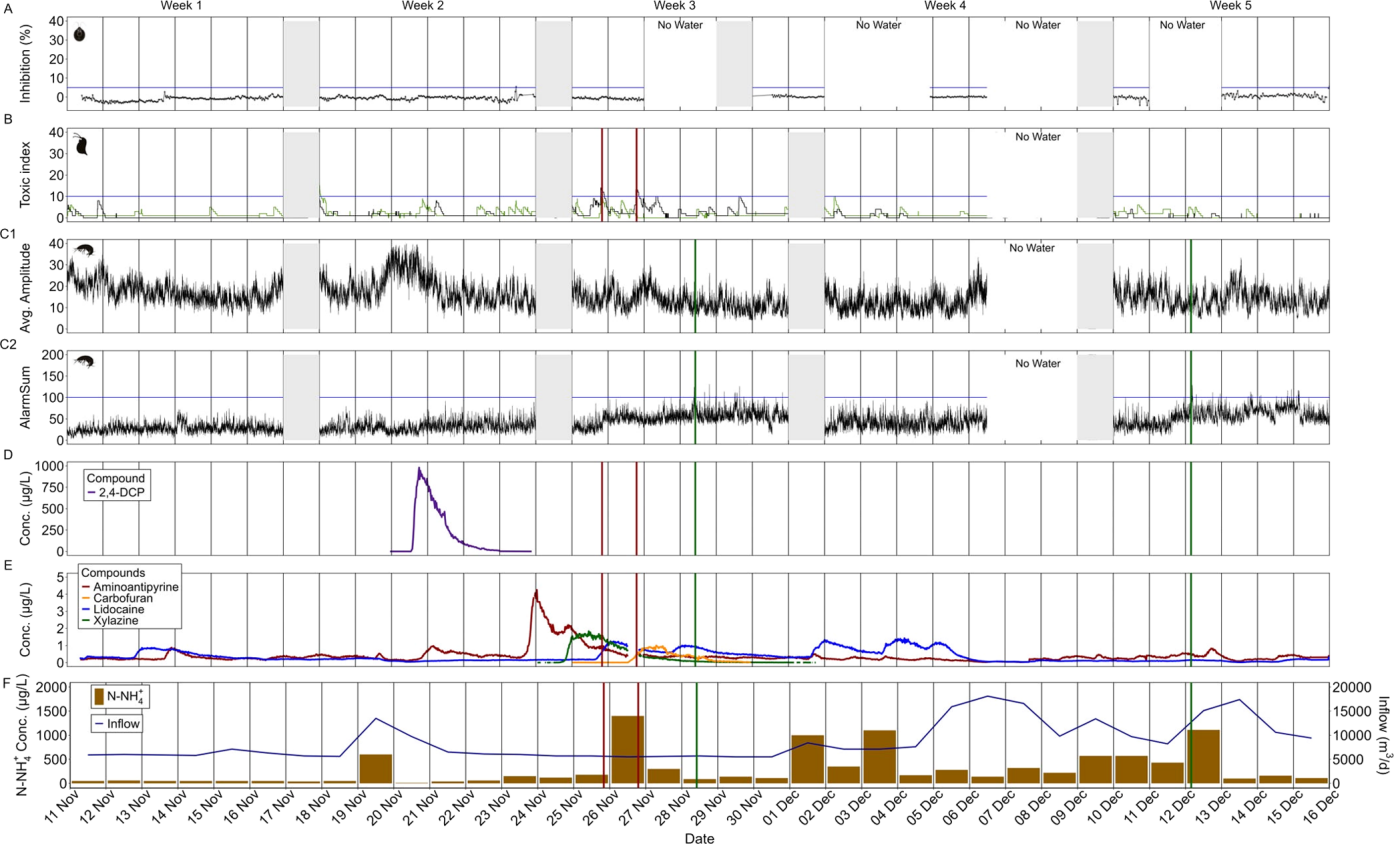
Complete measurement period of the BEWS, MS2Field, inflow rate and N-NH_4_^+^ at the WWTP. There were technical problems in the third, fourth and fifth weeks, during which no valid measurements with the respective BEWS could be recorded for 1–3 days indicated by “no water”. The gray shaded area represents maintenance days with organism exchange. (A) Algae Toximeter—Photosynthesis inhibition (%) of *C. vulgaris* in black. Blue horizontal line indicates the alarm threshold (B) DaphTox II—Toxic index of *D. magna*: Green and black lines represent behavioral activity in the test chambers 1 and 2. The blue horizontal line indicates the alarm threshold. Vertical solid red lines indicate triggered alarms. (C1) Sensaguard—Average amplitude of *G. pulex*: Black lines show the average behavioral activity of all eight organisms. Vertical solid dark green lines show alarms. (C2) Sensaguard—Alarmsum of *G. pulex*: Black lines show the alarm parameter for the behavioral activity of all eight organisms. The horizontal blue line shows the alarm threshold. The vertical solid green lines show alarms. (D) MS2Field—concentration trend of 2,4-DCP in μg/L. (E) MS2Field—concentration trends of aminoantipyrine, carbofuran, lidocaine and xylazine in μg/L. (F) N-NH_4_^+^ concentrations (μg/L) as brown bars and WWTP influent (m^3^/day) as dark blue line, measured daily.

During our study at a municipal WWTP for 5 weeks, the Algae Toximeter showed no significant deviations of the photosynthetic activity of *C. vulgaris* between the effluent and the reference water in the system, and therefore the alarm threshold was not exceeded (Figure 2A). Herbicides are the most important group affecting photosynthesis. As there are no known pesticide production sites in the catchment, and a weed control for agriculture is not required in winter, the absence of photosynthesis inhibiting herbicides in November and December was expected.

The DaphTox II and Sensaguard exhibited several behavioral irregularities and alarms throughout the monitoring campaign (Figure 2B,C), which will be systematically described in the subsequent sections. The Sensaguard triggered an alarm on December 12 following the death of an individual gammarid (Figure 2C2). Since it is assumed that the death was due to natural causes rather than chemical exposure, it can be implied that the alarm threshold would not have been exceeded if the individual gammarid had survived. Therefore, this event will not be discussed further. For additional details see S7 in SI.

#### Rain Event Caused Increased Behavioral Activity in *G. pulex*

During the second week, the DaphTox II showed no deviation from normal responses in *D. magna* (Figure 2B). However, in *G. pulex*, an increase in behavioral activity was observed shortly after a rain event increased the inflow rate from 5610 to 13 500 m^3^/L (Figure 2F), although this rise in activity was not accompanied by an alarm in the Sensaguard system (Figure 2C1,C2).

Regarding the abiotic parameters, the rain event led to a decrease in effluent conductivity (from 1.1 to 0.5 mS/cm), pH (from 8.3 to 7.8), and temperature (from 18.5 to 16.5 °C) within 24 h. The change in conductivity was not expected to have a major effect on the test organisms as the values (0.5–1.2 mS/cm) were within the tolerance ranges of *D. magna* (24 h-EC_50_ = 5.9 mS/cm)^35^ and *G. pulex* (72 h-EC_50_ = 12.8 mS/cm).^36^ Regarding pH, no obvious change in the behavior of *D. magna* and *G. pulex* was expected, since pH in aquatic habitats naturally fluctuates between 6.0 and 8.0, and the observed change in pH was generally small (0.5). The 2 °C drop in effluent temperature may have affected the gammarids in the Sensaguard since this biomonitor does not have an own thermostat to independently regulate the water temperature like the other BEWS used. Although the temperature drop was relatively small, it is known that subtle changes in temperature can affect behavior such as ventilation in freshwater organisms like *G. pulex*. For example, the locomotor activity of *G. pulex* was found to vary with temperature (0–25 °C), with a significant increase in locomotion at temperatures above 20 °C.^37^ In natural environments, temperature changes are not uncommon and most species, including *G. pulex*, have some degree of resilience to cope with such fluctuations, but according to the presented literature, a behavioral response toward a slight increase or decrease in temperature cannot be excluded. Regarding chemical concentrations, the analysis of measured target compounds, lidocaine, xylazine, and aminoantipyrine, revealed that the increase in the inflow led to a dilution of these compounds in the effluent. Consequently, a compound-specific response was deemed unlikely in this scenario. To explore the possibility of unknown compounds contributing to the observed effects, a NTS was conducted using the MS2Field data. No compounds correlated with gammarid behavior and with known toxic effects on the test organisms were detected at elevated levels in the NTS, with the notable exception of the plant growth regulator herbicide 2,4-dichlorophenoxyacetic acid (2,4-D) and its metabolite 2,4-dichlorophenol (2,4-DCP) (Figure 2D). Quantification revealed remarkable concentrations of approximately 300 μg/L for 2,4-D and 1000 μg/L for 2,4-DCP. However, it is essential to acknowledge that this estimation may not fully address the extent of matrix effects and other sources of variability inherent in retrospective quantification methods. The concentrations reported should be interpreted with caution, as a more comprehensive assessment involving calibration curves directly prepared in wastewater matrices would provide greater confidence in the accuracy of quantification. This refinement is particularly relevant for retrospective quantification, where wastewater matrix effects can significantly impact the results as reported by Köke et al.^20^ Despite the substantial concentrations detected, the Algae Toximeter did not exhibit a response. This lack of response may be attributed to the fact that the primary mechanism of action of 2,4-D does not target photosynthetic activity in algae but rather growth and other metabolic processes within the algae. Comparison with publicly available toxicity values of *D. magna* and *G. pulex* indicated that the measured concentration for 2,4-DCP was respectively three to 4-fold lower than the reported effect concentrations in acute toxicity tests in the literature.^38^ Using automated swimming activity monitoring, Bahrndorff et al.^39^ demonstrated that 1200 μg/L of 2,4-DCP significantly reduced *D. magna* activity after 6 h of exposure. However, despite the proximity of effect concentrations, no discernible influence on *D. magna* behavior was observed during effluent monitoring in the present study. Gammarids activity reached maximal levels 12 h before the peak concentration of 2,4-DCP was recorded by the MS2Field. We utilized a GLMM to explore the relationship between organismal behavior, compounds and abiotic factors. To test the null hypothesis that each predictor has no effect on the response variable behavior, an analysis of variance with a Type II Wald chi-square test was applied. Notably, our findings unveiled a significant correlation (*p* < 0.03) between temperature and gammarid behavioral patterns, among the parameters examined. Given the absence of triggered alarms and the lack of elevated compound concentrations identified through NTS, we interpreted the higher gammarid behavior activity in this case as likely a response not induced by specific compounds, but rather by the slight decrease in temperature. In addition, it has to be kept in mind that, although NTS is a valuable tool for identifying unexpected contaminants, it is inherently limited by factors such as detection limits, ionization efficiencies, and matrix complexity. Therefore, the absence of detected compound peaks does not exclude the possibility that undetected substances could have contributed to the behavioral response.

Considering the potential influence of temperature on organism behavior, temperature control remains a critical factor in minimizing confounding variables in BEWS. Future systems should incorporate a more powerful thermostat to avoid fluctuations and reduce the risk of misleading interpretations driven by abiotic factors such as temperature changes.

#### BEWS Responses Cannot be Linked to Elevated Concentrations of Selected Target Compounds

During the third week of the measurement period, both test organisms, *D. magna* in the DaphTox II (Figure 2B) and *G. pulex* in the Sensaguard (Figure 2C), showed behavioral changes that triggered an alarm. The alarm threshold of the DaphTox II was exceeded twice within an interval of 24 h. During the first alarm, the toxic index parameter, which calculates the deviation from normal behavior of daphnia by measuring individual parameters, was strongly influenced by the fluctuating swimming height and the swimming distance between the organisms. The second alarm on November 26 in chamber two was mainly caused by the parameter “number of active organisms”. It should be noted that the DaphTox II camera does not always detect individual daphnia for short periods of time when the organisms move close to the grating where the wastewater exits the test chamber. The undetected daphnia are counted as dead by the system algorithm and will therefore affect the alarm parameter, technically contributing to a false alarm. However, a noticeable change in the swimming position can also be categorized as typical avoidance behavior resulting in increased swimming speed,^40^ which is an important input to the alarm parameter.

The Sensaguard triggered an alarm 1 day after the second alarm of the DaphTox II. During the first 2 days of this third week, the typical circadian behavior of *G. pulex* was observed, represented by the activity value, which is the average of the 8 individual gammarids^41^ (Figure 2C1). This circadian pattern decreased in the second half of the week, which led to the exceedance of the alarm threshold (Figure 2C2). The Alarmsum parameter increased with the death of one individual gammarid on November 25 and indicated an exceedance of the alarm threshold on November 28 (Figure 2C2). Any influence of technical problems or abiotic parameters was checked during the third week according to the strategy shown in Figure 1. All parameters remained constant except for the N-NH_4_^+^ concentration, which reached 1400 μg/L in the effluent on November 26 (Figure 2F). According to the abiotic conditions in the effluent (pH of 8 and temperature of 16 °C), the concentration of un-ionized ammonia (NH_3_) was approximately 41 μg/L. Ammonia affects a variety of freshwater species, including small invertebrates such as gammarids and daphnia, and can be explained by similar mechanisms such as gill damage and nervous system effects.^42^ According to Serra et al.,^43^ performance of daphnia in terms of swimming speed, filtration rate and mortality would not be expected to change if the concentrations of N-NH_4_^+^ and NO_2_^–^ remained below 5000 and 35 000 μg/L, respectively, which is 3–25 times higher than the measured concentration in the effluent in our study. It is also a factor of 38 lower than the 96 h-LC_50_ (1540 μg/L) for NH_3_ determined by Prenter et al.^44^ for *G. pulex*. We assumed that the observed ammonium concentration was far below the levels relevant for responses such as behavioral changes in the test organisms.

Chemical analysis revealed three target substances during this event: lidocaine, xylazine and aminoantipyrine, which were present in increased concentrations during the alarm phase in the third week (Figure 2E and Table 1). Lidocaine, is a local anesthetic that blocks voltage-gated Na^+^ channels in the neuronal cell membrane and belongs to the amide group. Different compounds belonging to this group (tetracaine and bupivacaine) can alter the behavior of *D. magna* at high concentrations (16 000–256 000 μg/L), leading to an increase in swimming speed.^45^ The effective concentration of lidocaine for *D. magna* is a factor of 250 000 higher than the concentration found in the effluent (1.2 μg/L, Table 1).^46^ As shown in Figure 2E, lidocaine was detected at similar concentrations in the fourth week again. As no alarms or sudden behavioral changes were observed in either group of organisms during the fourth week, we considered that direct effects of lidocaine at the analyzed water concentration on the test organisms were unlikely.

**Table 1:** Peak Concentrations of Target and Non-Target Compounds during Week 3 in Figure 2E^a^

compound	substance group	target or nontarget?	max concentration in effluent (μg/L)	EC_50_/LC_50_ (μg/L) *Daphnia sp.*	RQ (MEC/EC_50_)	sum RQ-*Daphnia sp*. contribution to total risk (%)	reference	EC_50_/LC_50_ (μg/L) *Gammarus sp.*	reference
lidocaine	anesthetic	target	1.2	308 800 (24 h)	3.9 × 10^–6^	0.04	Lomba et al.^46^	no data	
xylazine	sedative	target	1.8	48 200 (48 h)	3.7 × 10^–5^	0.4	Bayer^48^	no data	
aminoanti-pyrine	analgesic	target	4.2	13 500 (24 h)	3.1 × 10^–4^	3.5	Wünnemann et al.^49^	no data	
carbofuran	insecticide	nontarget	1.4	168 (24 h)	8.6 × 10^–3^	95	Barata et al.^55^	21 (24 h)	Ashauer et al.^38^

a Measurement was performed by the MS2Field.

The second compound, xylazine, used as a veterinary sedative, is an agonist at the α2 class of adrenergic receptors and leads to a decrease in the neurotransmission of norepinephrine and dopamine in the central nervous system. Very little is known about the ecotoxicological effects of such substances. 48 h-EC_50_ values from safety data sheets are at 15 500–43 200 μg/L for daphnia,^47,48^ which is again a factor of 1000 above the concentration we found in the effluent (1.8 μg/L, Table 1).

The third target compound, aminoantipyrine, an analgesic drug, was found at the highest concentration of the targets in the effluent (4.2 μg/L, Table 1). Compared to the other two targets, the analgesic has the lowest 24 h-EC_50_ (13500 μg/L) for *D. magna*.^49^ With respect to sublethal effects on *G. pulex*, the literature shows that much lower doses (10–100 ng/L) of xenobiotics such as the analgesic ibuprofen can reduce amphipod activity.^50^ Ashauer et al.^38^ compared the sensitivity of *D. magna* and *G. pulex* to organic xenobiotics in acute toxicity tests. Single sensitivity differences were found for neonicotinoids and pyrethroids, where *G. pulex* was more sensitive by a factor of 100–1000, but in conclusion, both organisms were equally sensitive to xenobiotics. For this evaluation, we assumed that the toxicity of the three target substances is comparable between *D. magna* and *G. pulex*.

Given the maximum concentrations of the single target substances measured in the effluent, based on toxicity data from the literature, we concluded that none of the target compounds were present in concentrations high enough to cause behavioral responses in the organisms in the BEWS that would have induced an alarm. According to our research strategy, the target compounds were excluded from stage I laboratory experiments due to their EC_50_ values exceeding those detected in the effluent by factors of 100 or 1000. However, it cannot be ruled out that the mixture of substances may have triggered an effect.

#### Non-Target Screening Reveals the Detection of Carbofuran in Wastewater

The NTS in week 3 identified the insecticide carbofuran, which appeared at a relevant concentration simultaneously to the detected target compounds in the third week (Figure 2E). This detection unexpected, given that this insecticide has been banned in the European Union since 2007 (in Switzerland since 2011). However, it has been reported that carbofuran is still used illegally in intentional animal poisoning.^51^ In the literature, a 24 h-EC50 value of 168 μg/L for carbofuran has been reported for daphnia immobility^52^ and a significantly lower LC50 value for gammarid lethality (24 h-LC50 = 21 μg/L; Ashauer et al.)^38^ (Table 1). These differences in sensitivity underscore the utility of gammarids as sensitive bioindicators for detecting low concentrations of contaminants such as carbofuran.

In the retrospective analysis, carbofuran’s identity was confirmed using a reference standard and a maximum effluent concentration of 1.4 μg/L was quantified based on a five-point calibration. However, it is crucial to acknowledge the limitations of this retrospective approach, as discussed in the Materials and Methods section, particularly since the calibration was conducted in ultrapure water. Based on the small difference between the estimated concentration and the expected toxicity, also compared to the target compounds, which was also indicated by the calculated RQ (Table 1), we concluded that carbofuran was the most likely causative compound that triggered the alarm of the Sensaguard. The GLMM analysis showed that the insecticide was the most significant predictor in our model, with a *p*-value of less than 0.001 (0.000472) for gammarid behavior (for detailed statistical information see Section S5 in SI). Therefore, carbofuran was selected for the following laboratory experiments.

#### Exceedance Events and Hypothesized Causes in BEWS Monitoring

Table 2 provides an overview of the key findings regarding BEWS responses, listing the potential causes for each event, the system involved, and the supporting data (such as abiotic parameters and chemical concentrations). It highlights that many alarms were due to false triggers or natural causes, while carbofuran detection stood out as a likely cause for toxicity responses in week 3.

**Table 2:** Summary of BEWS Responses, Linking Hypothesized Explanations to the Observed Behavior of Test Organisms and Relevant Factors

week	BEWS involved	event	hypothesized explanation	supporting evidence
week 2	Sensaguard (*G. pulex*)	increase in behavioral activity, no alarm	slight decrease in water temperature after rain event caused behavioral change	temperature drops from 18.5 to 16.5 °C; *G. pulex* sensitive to even small temperature changes; no toxic compounds detected
week 3	DaphTox II (*D. magna*)	exceeded alarm threshold (alarm 1)	possible false alarm due to camera not detecting organisms close to chamber grating	camera system limitations; no supporting chemical or abiotic evidence of toxicity
week 3	DaphTox II	alarm caused by the parameter “number of active organisms” (alarm 2)	false alarm due to system error, organisms undetected near grating	undetected organisms counted as dead by system algorithm
week 3	Sensaguard	exceedance of alarm threshold after death of one individual gammarid (alarm 3)	death of *G. pulex* on November 25 likely due to natural causes	no relevant chemical concentrations detected; abiotic factors constant
week 3	Sensaguard and DaphTox II	slight increase in N-NH_4_^+^ concentration (1.4 mg/L)	N-NH_4_^+^ levels far below effect thresholds	N-NH_4_^+^ concentration well below toxic levels for test organisms
week 3	Sensaguard and DaphTox II	increased concentrations of target compounds lidocaine, xylazine, and aminoantipyrine	concentrations too low to cause behavioral changes	lidocaine (1.2 μg/L), xylazine (1.8 μg/L), aminoantipyrine (4.2 μg/L) present but below effective concentrations for behavioral effects
week 3	Sensaguard and DaphTox II	increased concentration of carbofuran, alarm triggered by behavioral changes	carbofuran presence likely cause	detected concentration of 1.4 μg/L close to known toxicity thresholds for *G. pulex*

#### Laboratory Experiments with Carbofuran

To verify the field results carbofuran was spiked to effluent of the Eawag pilot WWTP as in Kizgin et al.^16^ In the first stage, the organisms in the BEWS were exposed to the 3-fold measured environmental concentration of carbofuran and no behavioral changes or exceedance of the alarm threshold was observed in the daphnia. However, gammarids responded with increased activity during the exposure phase. This qualified the insecticide for the second stage verification test. For detailed results of laboratory experiments of stage I see Section S4.2 in SI.

**Figure 3:**
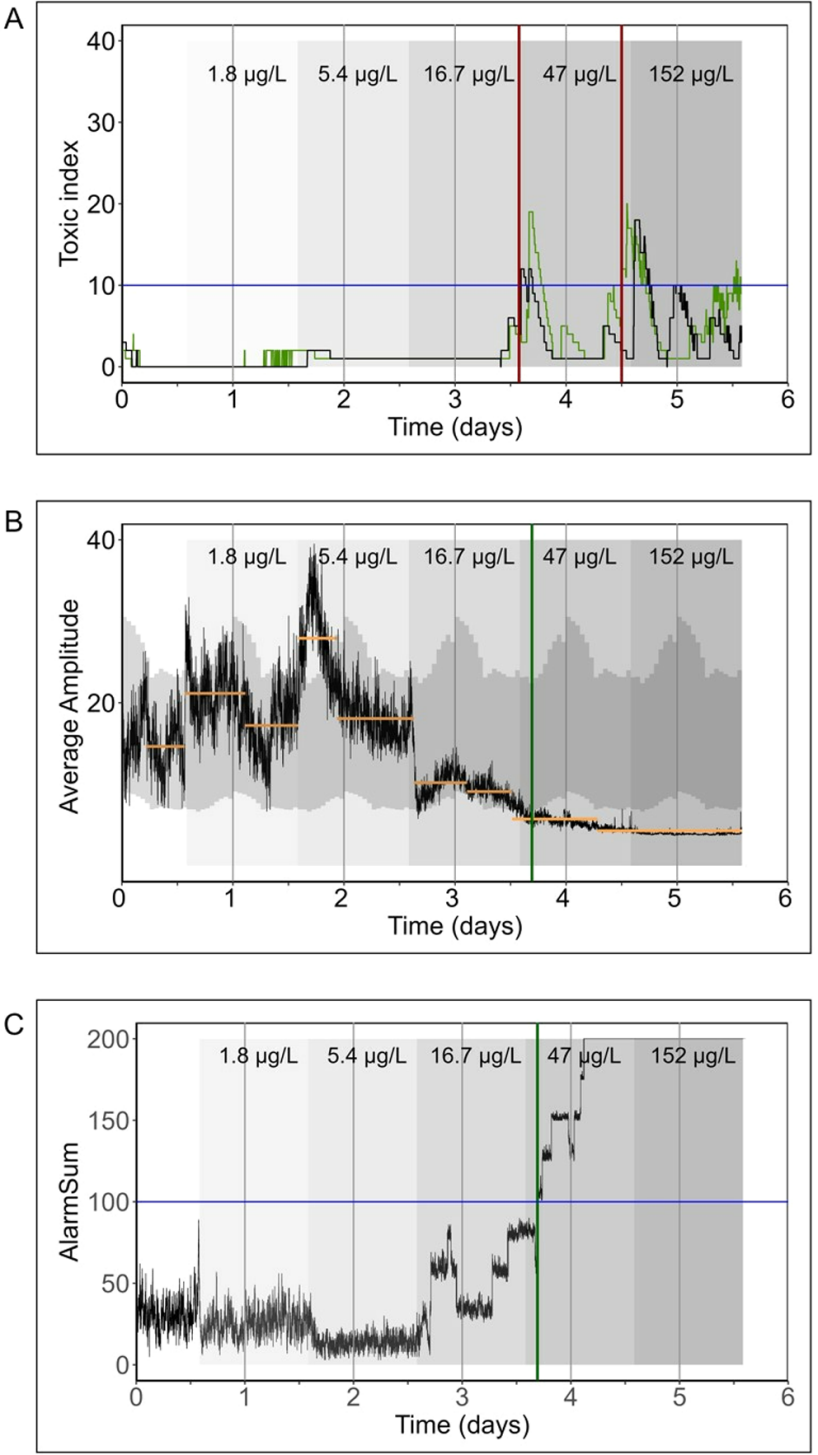
Stage II of laboratory experiments. Behavioral response of test organisms in the BEWS to measured concentrations of carbofuran. (A) DaphTox II—measured toxic index of *D. magna*: blue horizontal line indicates alarm threshold. Green and black lines represent behavioral activity in test chambers 1 and 2. The vertical red lines show alarms (B) Sensaguard—measured average amplitude of *G. pulex*: black lines represent the average behavioral activity of all 8 organisms. Horizontal yellow lines indicate the mean of each segment whose length is determined by the time-points detected in the multivariate changepoint analysis. Gray ribbons indicate model of 95% confidence intervals. Vertical green line shows the alarm. (C) Sensaguard—calculated Alarmsum of *G. pulex*: black lines show the alarm parameter for the behavioral activity of all eight organisms. Blue horizontal line indicates alarm threshold. The vertical green line shows the alarm.

In the second stage of the laboratory experiments, the organisms were exposed to increasing concentrations of carbofuran over periods of 24 h to assess the concentration level at which the compound affected their behavior. The exposure concentrations were determined analytically as 1.8, 5.4, 16.7, 46.9, and 152.1 μg/L. For the daphnia, we observed a cyclic pattern in the toxic index of the DaphTox II following each concentration increase, attributed to the fixed holding time of 300 min (Figure 3A). This means that all toxic points of an alarm type (e.g., on the speed classes) except of the number of organisms are gradually reduced after 300 min. Our findings revealed a response from *D. magna* at a concentration of 16.7 μg/L, with mortality observed at 150.1 μg/L, consistent with the 24 h-EC_50_ value of 168 μg/L reported by Barata et al.^52^ (Figure 3A). However, behavioral alterations were detected starting at 16.7 μg/L, indicating that daphnia are more sensitive to carbofuran than previously anticipated, as found also for other compounds.^39^ These results emphasize the significance of behavioral end points as sublethal indicators and caution against underestimating their ecological relevance. Additionally, the concentration affecting daphnia behavior in the verification test is also more consistent with an older study where the 24 h-LC_50_ value was determined as 44.7 μg/L.^53^ Despite the sensitivity of daphnia to carbofuran, our analysis of the measured concentrations in the municipal WWTP suggests that the levels of carbofuran were insufficient to elicit a significant behavioral response in *D. magna*. However, mixture effects with other chemicals cannot be excluded.

For *G. pulex*, the SMOP algorithm of the multivariate analysis detected an increased diurnal rhythmicity of *G. pulex* with increasing insecticide concentration (Figure 3B). The increased activity at 1.8 μg/L and the exceedance of the normal behavioral level defined by bootstrap analysis of baseline data (gray ribbons) at 5.5 μg/L, indicated an escape movement typical for avoidance behavior.^15^ Mortality in the Sensaguard and the first alarm occurred at 16.7 and 47 μg/L (Figure 3C). The organism response occurred below the effect concentration for this compound measured by Ashauer et al.^38^ (24 h-LC_50_ = 20 μg/L). This showed that it is highly recommended to evaluate and statistically analyze the average amplitude from the raw data of the system, in addition to the alarm algorithm calculated by the system, as highlighted by Kizgin et al.^16^ Both the reaction of the gammarids in stage I and the behavioral activity in stage II showed that carbofuran at concentrations between 1.8 and 5.4 μg/L can certainly influence the behavior of the gammarids. Additional additive mixture effects cannot be excluded. Based on these findings, we assumed that carbofuran was mainly responsible for the observed changes in gammarid behavior in the municipal WWTP.

### Rapid Methods for Monitoring WWTP Effluent–Opportunities and Limitations

In recent years the application of BEWS has started to gain momentum and studies by Gerhardt et al.^54^ and Ruck et al.^15^ showed the potential of using BEWS for wastewater monitoring with amphipods focusing on single biomonitoring systems. Kizgin et al.^16^ has demonstrated that the use of multiple systems with organisms of different trophic levels and different end points simultaneously can be beneficial for acquiring critical information from WWTP effluents. The present study showcased for the first time the simultaneous use of multiple BEWS in combination with online LC-HR-MS/MS analysis (MS2Field), and demonstrated the potential of BEWS as a technique for measuring adverse effects in wastewater.

To increase the reliability of BEWS data and improve wastewater quality assessment, we developed a decision-making strategy. This helped in the interpretation of BEWS signals, which can be complex and may require experience to notice gradual or subtle changes in organism behavior to avoid false positive alarms, as highlighted by Bownik and Wlodkowic.^11^ We pointed out that a full understanding of the behavior of organisms in BEWS requires consideration of (1) technical aspects such as water supply interruption and temperature control, (2) the monitoring and influence of physicochemical parameters such as ammonium levels, (3) the implementation of chemical screening for high-intensity signals to identify potential causative compounds, (4) the screening of the literature for compounds with elevated concentrations to assess their potential contribution to the observed effects, and (5) the introduction of a risk quotient (RQ) to prioritize compounds for laboratory confirmation. The aforementioned steps should also be pursued to standardize and align validation approaches with guidelines for BEWS deployment as part of broader protocols for water monitoring and chemical risk assessment. Regarding chemical analysis, moving beyond target screening to nontarget retrospective analysis without preselecting or purchasing standards,^55^ helped to detect potential causative compounds in the background. Moreover, innovative approaches such as Virtual Effect-Directed Analysis (Virtual EDA)^56−59^ could be explored on the MS data to identify potential toxicants. The integration of BEWS and Virtual EDA could provide a cost-effective and continuous analytical innovation for chemical identification of toxicity drivers following BEWS alerts. Biological and LC-HR-MS/MS methods may be increasingly applied in future wastewater monitoring concepts. Beyond municipal wastewater treatment, BEWS have significant potential for applications in other environmental management areas. In industrial contexts, BEWS can be particularly advantageous, as they can adapt to the rapidly changing wastewater composition associated with different industrial clusters and multiple production lines. Similarly, in agricultural settings, BEWS can monitor runoff and assess the impact of pesticides and fertilizers on local water bodies, thus guiding sustainable agriculture practices. By providing continuous, real-time toxicity assessments, BEWS could trigger treatment efficiency improvement, and reduce environmental risks by rapidly identifying and mitigating harmful substances. Future studies should evaluate whether the application of BEWS, combined with reaction-triggered sampling and laboratory-based chemical analyses, can be further enhanced by improving the quantification approach. Potential improvements include the use of matrix-matched calibration curves to better account for matrix effects (e.g., preparing standards directly in wastewater samples) as well as expanding the use of isotopically labeled internal standards for each target compound, and exploring advanced signal correction methods. These refinements, along with the integration of Virtual EDA, would strengthen the precision of chemical identification and risk assessment for toxic drivers following BEWS alerts. The relevance of this approach is underscored by the recent EU Parliament negotiations that agreed on a deal to enhance the efficiency of municipal wastewater treatment and reuse through improved monitoring of wastewater content.^60^ The findings of the present study indicate that BEWS, particularly when integrated with robust abiotic and chemical data, can pave the way for forthcoming environmental monitoring and management practices to reach the goals of the European zero pollution action plan.

## Supporting Information Available

Details on BEWS; chemicals; statistics; analytical method; quantification; data processing; source allocation; and physiochemical parameters (PDF)
List of WWTP parameters; general water chemistry and analytical results (XLSX)

## Abbreviations

2,4-D: 2,4-dichlorophenoxyacetic acid
2,4-DCP: 2,4-dichlorophenol
BEWS: biological early warning systems
EC: effect concentration
EDA: effect-directed analysis
ILIS: isotopically labeled internal standards
LC: lethal concentration
LOQ: limit of quantification
LC-HRMS/MS: liquid chromatography-high resolution mass spectrometry
MEC: measured environmental concentration
MP: micropollutant
NTS: nontarget screening
RQ: risk quotient
SI: supporting information
WWTP: wastewater treatment plant
