# Supplementary material for: Integrating Biological Early Warning Systems with High-Resolution Online Chemical Monitoring in Wastewater Treatment Plants
Source: Environ Sci Technol. 2024 Dec 18;58(52):23148–59. doi: 10.1021/acs.est.4c07316 (PMC11697333; doi:10.1021/acs.est.4c07316)
Supplement: Supplementary file 1 — es4c07316_si_001.pdf [file es4c07316_si_001.pdf]

Integrating Biological Early Warning Systems with High-Resolution Online Chemical Monitoring in Wastewater Treatment Plants

Supplementary Information

Ali Kizgin*[a,d], Danina Schmidt[b,c], Julian Bosshard[d], Heinz Singer[d], Juliane Hollender[d,e], Eberhard Morgenroth[d,f], Cornelia Kienle[a], Miriam Langer[d,g]

[a]Swiss Centre for Applied Ecotoxicology, 8600 Dübendorf, Zürich, Switzerland

[b]Eawag, Swiss Federal Institute of Aquatic Science and Technology, 8647 Kastanienbaum, Switzerland

[c]University of Tübingen, Animal Physiological Ecology, 72074 Tübingen, Germany[1]

[d]Eawag, Swiss Federal Institute of Aquatic Science and Technology, 8600 Dübendorf, Switzerland

[e]Institute of Biogeochemistry and Pollutant Dynamics, ETH Zürich, 8092 Zürich, Switzerland

[f]Institute of Environmental Engineering, ETH Zürich, 8092 Zürich, Switzerland

[g]Institute for Ecopreneurship, FHNW Muttenz, 4132 Muttenz, Switzerland

* Corresponding Author

Email: ali.kizgin@eawag.ch; Phone:+41 772676798

Number of Pages: 63

Number of Figures: 26

Number of Tables: 10

Table of Contents

S1 Organisms and Cultivation

S2 Methodology of Biological Early Warning Systems

S3 BBE Algae Toximeter

S4 BBE DaphTox II

S5 Sensaguard REMONDIS Aqua

S6 Technical Information on Membrane System

S7 Behavioral assessment

S8 Extended Results of BEWS at Municipal WWTP

S9 Comparison of Behavioral Activity of *G. pulex* with and without mortality

S10 Extended Results of BEWS Laboratory Tests with Carbofuran

S11 Statistics

S12 Statistical Methods for Biomonitoring Data

S13 Regression Model to Correlate Behavioral Data with Chemical and Abiotic Information

S14 MS2Field

S15 Technical Information

S16 Analytical Setup

S17 LC-HRMS Method

S18 LC-HRMS Settings

S19 Quantification of Selected Targets

S20 Non-Target Screening

S21 Identification and Quantification of Non-Targets

S22 Preparation and Analysis of Laboratory Experiment Samples

S23 Sample Preparation for Laboratory Experiments

S24 Quantification of Spike Samples of Laboratory Experiments

S25 Physicochemical Parameters

S26 Space and Time Requirements for Online Monitoring Tools

References

S1 Organisms and Cultivation

Chlorella vulgaris

A *Chlorella vulgaris* suspension from a laboratory culture at Eawag (Dübendorf, Switzerland) was used to cultivate the algae for the Algae Toximeter. The algae were kept in aerated separatory funnels (2 L) in a climate cabinet (1301XL, Rumed, Lostorf, Switzerland) at 20 °C with 3000 Lux (16:8 h) (Figure S1). For ventilation, sterile filters (Minisart High Flow, Sartorius, Göttingen, Germany) with a diameter of 0.45 µm were fitted to the tubes to exclude external particles and contamination. An algae culture of 2 L was introduced before every experiment and a small culture of 100 mL algae suspension was refilled with 2 L of nutrient solution as preparation for the upcoming experimental week (Table S1)

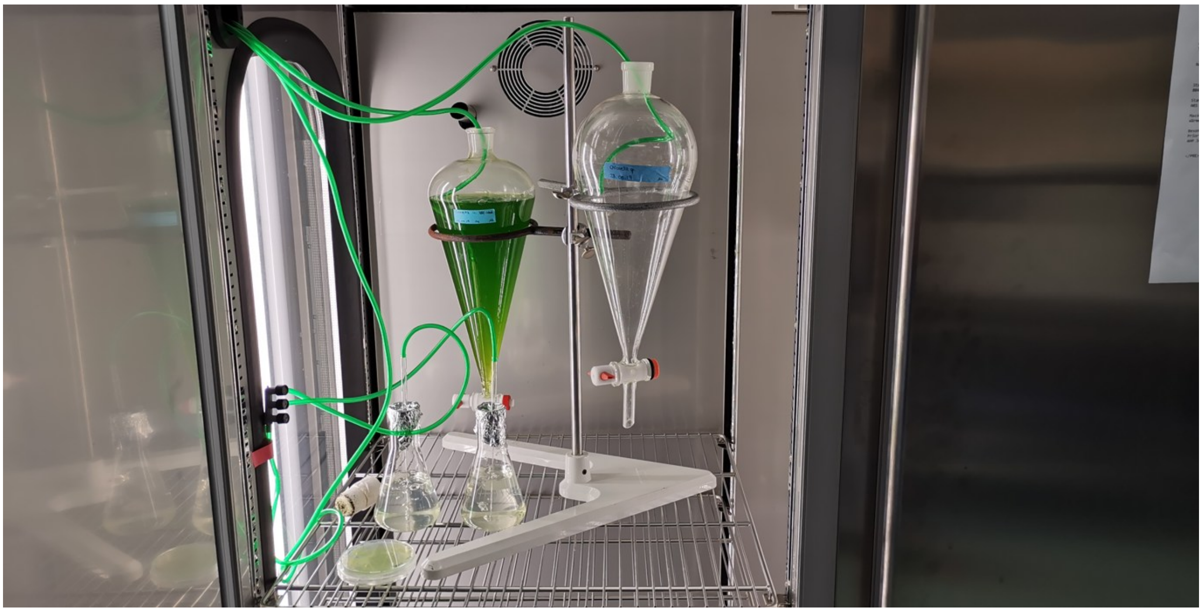

Figure S1. *Chlorella vulgaris* cultivation in separatory funnels placed in a climate cabinet

Table S1. Nutrient solution for algae cultivation

Chemicals	In mg	CAS	Vendor
$CaCl_2 * 2H_2O$	116	10035-04-8	*Sigma Aldrich*
Urea $CO(NH_2)_2$	5000	124-43-6	*Sigma Aldrich*
K_2HPO_4	2400	7758-11-4	*Sigma Aldrich*
KH_2PO_4	1533	7778-77-0	*Sigma Aldrich*
$MgSO_4 * 7 H_2O$	2000	10034-99-8	*Sigma Aldrich*
+ 10 mL of Micronutrient solution "Woods Hole"			
Chemicals for "Woods hole"	In mg	CAS No.	Vendor
Biotin	0.5	58-85-5	*Sigma Aldrich*
CoCl2 * 6 H2O	10	7791-13-1	*Sigma Aldrich*
$CuSO_4 * 5 H_2O$	10	7758-98-7	*Sigma Aldrich*
FeCl3 * 6 H_2O	1000	10025-77-1	*Sigma Aldrich*
H_3BO_3	1000	10043-35-3	*Sigma Aldrich*
$MnCl_2 * 4 H_2O$	180	13446-34-9	*Sigma Aldrich*
Na_2EDTA	25000	6381-92-6	*Sigma Aldrich*
$Na_2MoO_4 * 2 H_2O$	6	10102-40-6	*Sigma Aldrich*
Thiamin HCl	100	67-03-8	*Sigma Aldrich*
Vitamin B12	0.5	68-19-9	*Sigma Aldrich*
$ZnSO_4$ * 7 H2O	22	7733-02-0	*Sigma Aldrich*

Daphnia magna

Daphnia magna for the online-biomonitoring experiments came from a laboratory breeding at Eawag (Düebendorf, Switzerland). The culture was kept in 1 L glass jars at 20 °C and diurnal light conditions (16:8 h, 3000 Lux) in a climate cabinet (1301XL, Rumed, Lostorf, Switzerland) (Figure S2). They were cultivated in pre-filtered (grass fibre prefilter, 0.45 µm, Sartorius, Göttingen, Germany) lake water (Lake Greifensee, Zürich, Switzerland) and fed with 5 mL *Scenedesmus oblique* algae suspension every second day. *S. oblique* was cultivated in 200 mL Erlenmeyer flasks and transferred into a chemostat vessel (continuous culture device). For food preparation, the outflow of the chemostat was introduced into beakers and centrifuged for 10 min at 3000 rpm at 18 °C. The resulting algae concentration was stored in autoclaved (sterilized) lake water at 4 °C. A suspension was made from the algae concentrate and lake water using a digital photometer. The correlation curve between carbon content of the algae and photometer was made. The wavelength was 800 nm and a cuvette with a size of 1 cm x

1 cm. The resulting suspension had an absorbance value of 0.3-0.35 nm. The diluted algae suspension was stored in 4 °C in the dark. Fifty percent of the water in the jars was exchanged monthly. For the experiment individuals no older than 3 days were used.

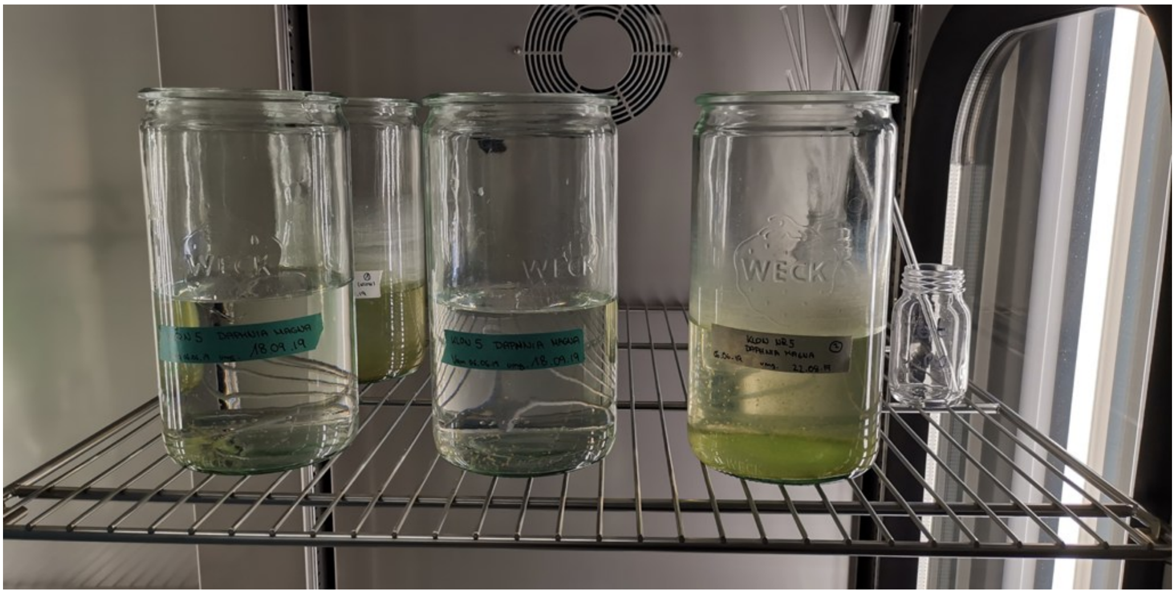

Figure S2. Cultivation of daphnia magna in climate cabinet

Gammarus pulex

Gammarus pulex were sampled using a sieve from an unpolluted tributary of the "Chriesbach" in a forested area close to Dübendorf, Switzerland (Latitude: 47.24277, Longitude: 8.37359). Less than 2 h after collection, the organisms were maintained in a 30 L aquarium with water from Chriesbach River at 17 ± 2 °C for a maximum of five weeks. A pump (505 S, Watson Marlow, Rommerskirchen, Germany) circulated the water through a filter (CristalProfi greenline e402 filter, JBL, Neuhofen, Germany). Before the experimental weeks, the organisms were kept for one week in aerated river water from "Chriesbach" where they had access to leaf litter (*Alnus glutinosa*). As size, sex and parasitism are known to influence the sensitivity of the test species only male adults (identified by their position in the precopular pair) with a cephalothorax width between 1.2 and 1.6 mm and visually uninfected by parasites were used in the experiments[1].

S2 Methodology of Biological Early Warning Systems

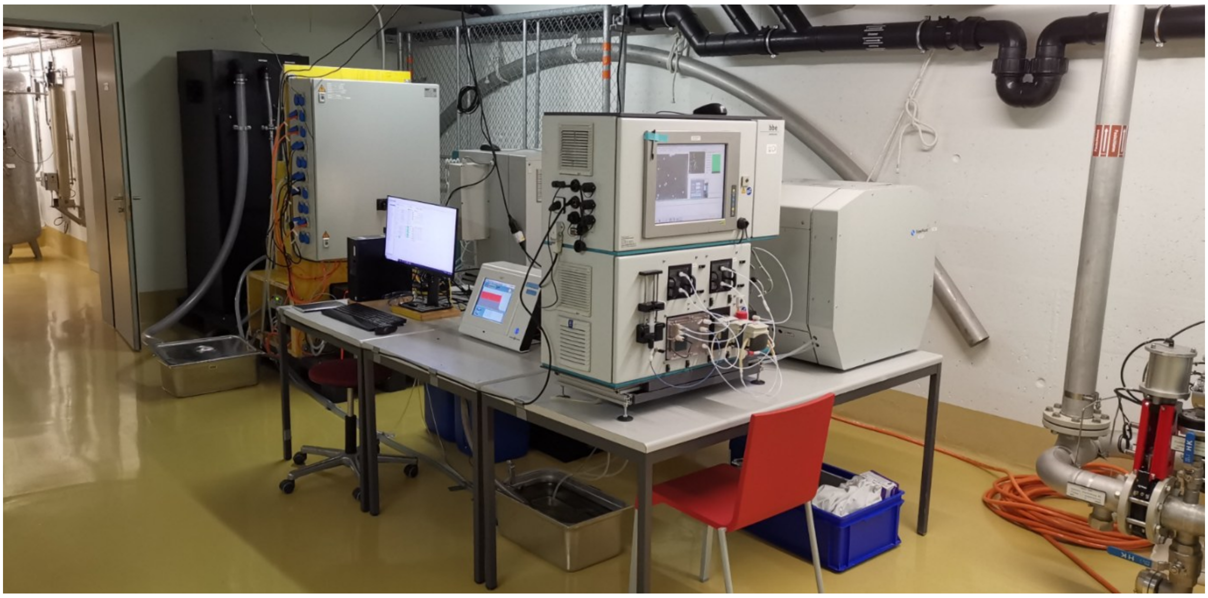

Figure S3. Setup of biological early warning systems (BEWS) and ultrafiltration system at municipal WWTP

S3 BBE Algae Toximeter

Principle

C. vulgaris were cultivated in a lighted, aerated and heated fermenter (2 L volume), which is an integrated component of the biomonitoring system (Figure S4). The temperature in the fermenter was regulated by a peltier element and a water-heat exchanger, which was connected to a circulation cooler (F32-HL, Julabo, Germany). The cooler was set to 17 °C and the peltier-element kept the temperature in the algae fermenter at 23 ± 1 °C. The hose system consists of different types of tubes. PVC tubes (food quality) are used in long supply and drain tubes. Interior tubes in contact with algae are made of PVDF while pump and valve tubes are made of silicone coated with platinum. The main sample pump uses Marprene tubing for long stability. Connectors and y-pieces are generally made of POM (polyoxymethylene) or stainless steel. Nutrient solution stored in a tank (Kautex, Germany) (Table S1) is supplied automatically to keep the algae concentration steady for 7 days. The turbidostatic principle allows the culture to

be always diluted with nutrient solution until it contains the right concentration. In order to take measurements, a small amount of the algae culture is transferred automatically along with sample water (which was in our case treated and filtered wastewater and will be addressed as “wastewater” in the following sections) to the chlorophyll sensor. By measuring the fluorescent response to light impulses of different intensities, the physiological activity of the photosynthesis is determined in the sensor unit. Since healthy algae lose smaller amounts of energy due to photosynthetic activity than damaged ones or even dead ones, the condition of the algae can be determined by measuring the reaction to small impulses of light. Normally the inhibition is expected to stay near 0 %. However, the occurrence of noise cannot be avoided, e.g. due to poor algae quality or a high algae background in the wastewater. That’s why the toxicity threshold was set to 5 %, to avoid a false triggering of an alarm. For the alarm evaluation two data points were considered.

The measurement process starts by the determination of the concentration of the different algae classes in the wastewater and their activity. The “Genty” Parameter, that indicates the activity of the algae in percent, is measured. The parameter is calculated by the measurement of the fluorescent response to a weak light impulse under two different conditions. One measurement takes place with (F_m) and one without a very bright background light (F_0) (equation 1)

(1) $$\text{Genty [\%]} = 100 \times \frac{F_m - F_0}{F_m}$$

Then, algae from the fermenter are added to the wastewater and again the concentration and the activity of the algae classes are determined. The values of the added algae are calculated from the difference, i.e., the number of algae added and their activity. This determines the condition of the algae in the sample. A further measurement, in which algae from the fermenter are added to drinking water rather than to the wastewater sample, is used as a reference.

(2) $$\text{inhibition [\%]} = 100 \times \left(1 - \frac{\text{Genty}_{\text{sample}}}{\text{Genty}_{\text{reference}}}\right)$$

The influence of wastewater quality on the photosynthesis activity is calculated by comparing the fluorescence of the algae ($\text{Genty}_{\text{sample}}$) in the water sample with reference water[2] ($\text{Genty}_{\text{reference}}$) (deionized water) (equation 2).

Each measurement of the wastewater sample, the wastewater sample + algae and the reference + algae is carried out in the same manner. The corresponding water is fed into the sensor. There the water is stored for a pre-defined period of time (incubation time). When the pre-defined incubation period has expired, the mixture is measured and the sensor is rinsed and refilled. The incubation time for the measurement intervals was set to 10 - 12 min (pre-setting by the system). The sample flow rate is about 30 mL per measurement cycle, which lasts around 45 min as the system operates in quasi-continuous mode. In this mode, measurements are taken at regular intervals, with a brief pause between each measurement.

More information is given in BBE's software and operating manuals for the Algae Toximeter[3].

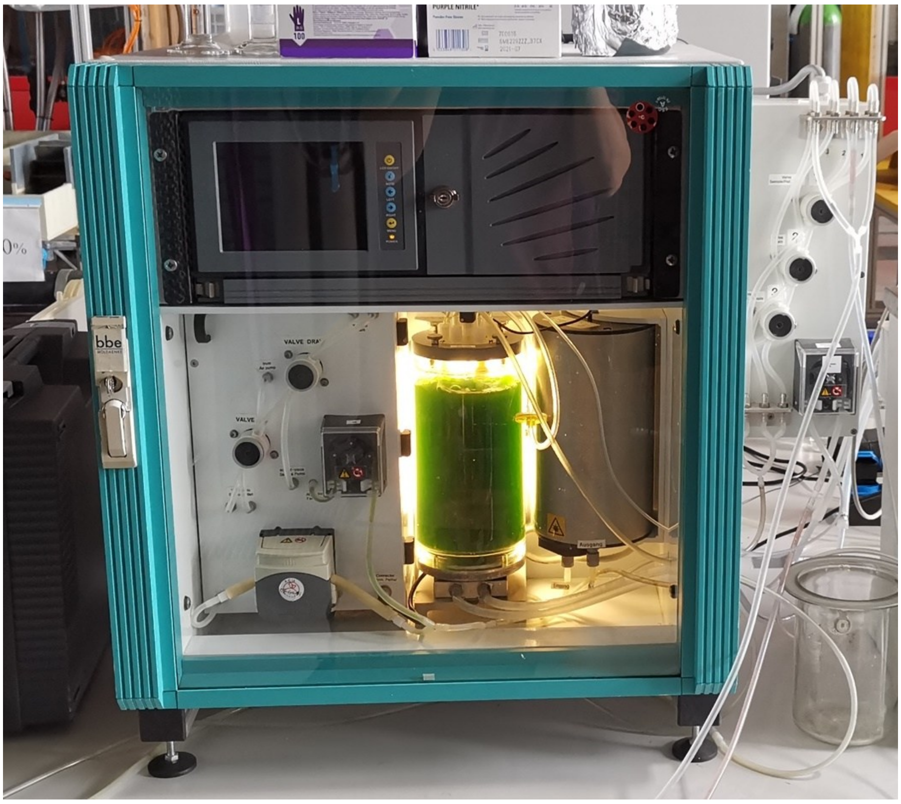

A

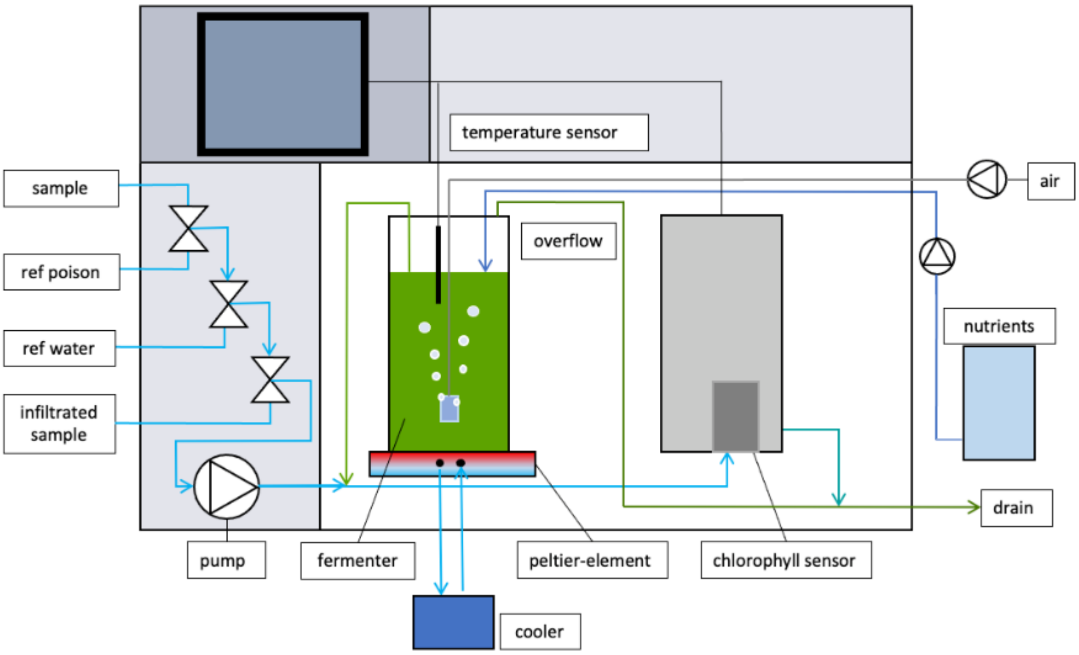

B

Figure S4. A. BBE Algae Toximeter, B. Diagram of the flow system of the Algae Toximeter

S4 BBE DaphTox II

Principle

The DaphTox II is a system with two measuring chambers and a computer unit (Figure S5). The wastewater is pumped through an ultrasonic filtering unit, which was not in operation due to the membrane filtration (see S6 Technical Information on Membrane System) in our case. The wastewater is passed through another filter step with glass wool and directed into a degassing unit for temperature control (kept at 20 °C). In this section an algae suspension (*C. vulgaris*) is continuously added as food source to the sample stream by a syringe pump to to ensure constant food supply. After the wastewater is passed through a little sponge filter, it is conveyed into the two chambers with 10 test organisms each, from where it is pumped into a drain. Two integrated video cameras, located behind the chambers, record the behavior of the organisms inside the red-illuminated test chambers and send the data to the computer. Illumination with red light cannot be detected by the daphnids. The image data are evaluated

and analyzed by the company-specific DTOX software and can be visualized with the DViewer software. The flow rate of the DaphTox II is 0.3 L/h with a residence time of 1-2 min. To reduce the deposit of particles in the supply hoses of the device, which may occur due to biofilm formation in the nutrient-rich wastewater, teflon tubes (VWR, USA) were used. The main pumps use marprene tubing for long stability. After each week, the test chambers of the DaphTox II were rinsed with deionized water, all tubes leading to the test chambers were replaced and the filters were removed and cleaned.

For behavior evaluation the toxic index, the number of active organisms and five behavior parameters are calculated[4]: the average swimming distance, which can indicate a change in social behavior, the average swimming height, which provides information on the vertical location in the chamber, the average swimming speed, which can either increase because of fleeing or decrease due to physiological damage, the speed-class index and the speed distribution. The speed distribution index is based on the momentary speeds of all individuals that are classified into 50 areas with 0 cm/s as the lowest class and >1.5 cm/s as the highest class. With the speed class index, only the particularly fast and the particularly slow individuals are taken into account. In addition, the fractal dimension describes the curve pattern in which the individuals move.The company-specific alarm principle is based on the Hinkley algorithm[5]. BBE DaphTox II uses the Hinkley algorithm to identify sudden changes in each parameter: The trends are searched for sudden changes using a mathematical calculation method. Statistically significant changes in the behavior of the organisms described by the different parameters are added up as toxic points and translated into the toxicity index. The holding time is set at 300 min. This means that all toxic points of an alarm type (e.g., on the speed classes) are gradually reduced after 300 min. The initial count of organisms has no follow-up time. The loss of each daphnia is permanently scored with the corresponding number of toxic points. The alarm

threshold for the experiments was set to toxic index=10. More information is given in BBE's software and operating manuals for the DaphTox II[6].

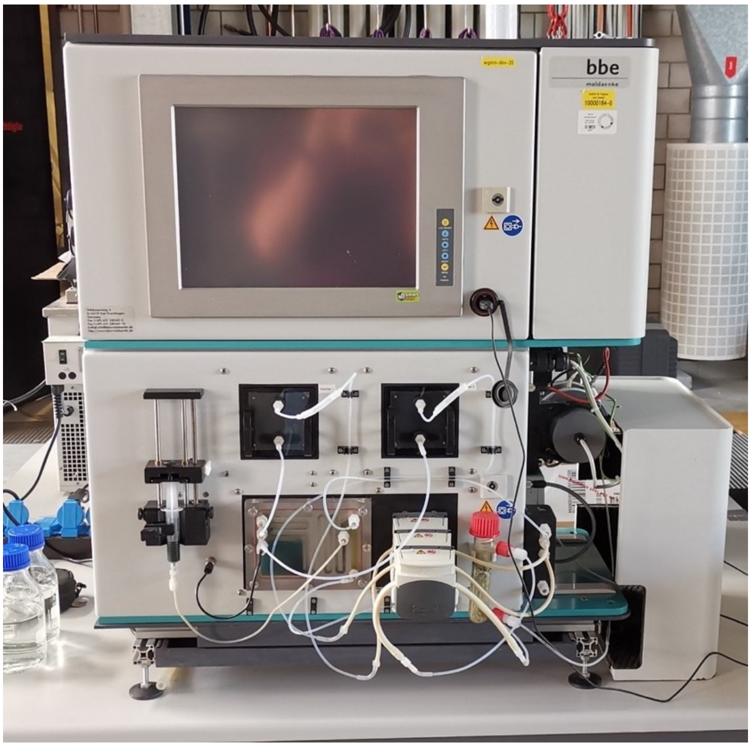

A

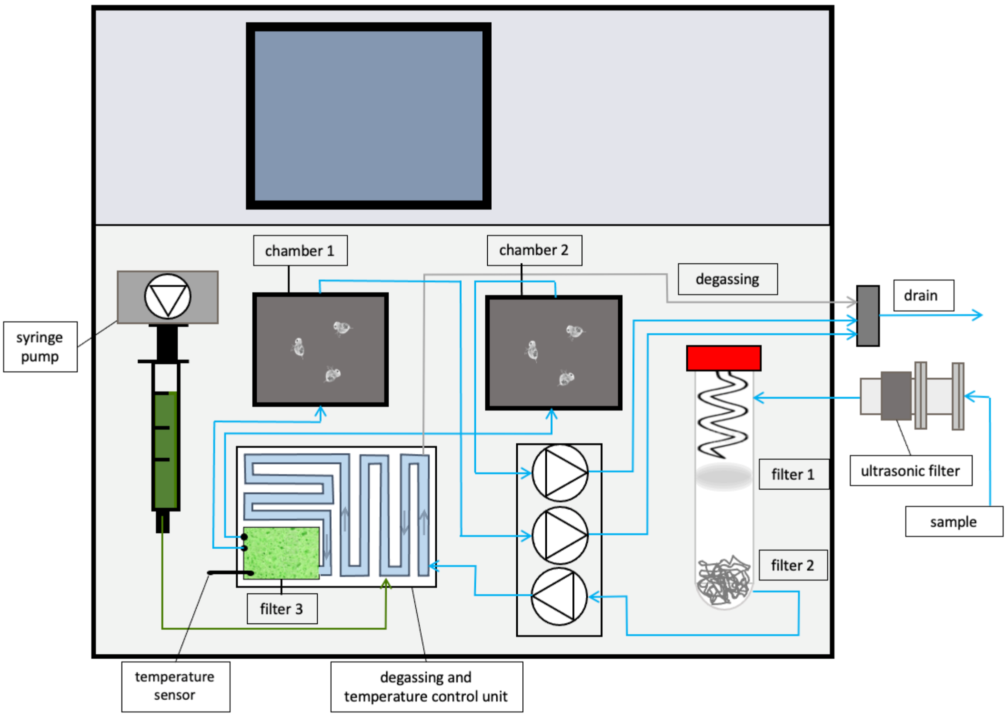

B

Figure S5. A. BBE DaphTox II B. Diagram of the flow system of the DaphTox II

S5 Sensaguard REMONDIS Aqua

The Sensaguard biomonitor consists of three main elements: the Sensaguard computer, which is the command and analysis unit of the system, the SensaFlow and the sensor chambers. The SensaFlow is the test unit of the system located in a 15 L glass basin (Figure S6): The sample water enters the basin from the left through the inlet. The water flows through the second part, filled with steel sponges to hold back coarse suspended matter from the sample, and subsequently to the third part, where the sensor chambers are located. Those are connected to the computer unit via cables to transfer the collected data. There are eight sensor chambers, with one test organism and three leaf disks (Ø = 17 mm) inside. The sample water flows through the chambers from below before it is discharged via the outlet. With this setup, a continuous flow of wastewater through the SensaFlow unit is achieved by a pump (Watson Marlow, UK). The flow rate was approximately 36 L/h and a residence time of approximately 25 min. For the maintenance after each experiment, the SensaFlow unit was rinsed with deionized water and the steel sponges were removed and cleaned. In previous experiments, it was discovered that the test organisms show an irregular daily pattern when left without food, which can influence the result[7]. During the experiment, the SensaFlow unit was covered with a cardboard box to exclude any influence from light. To transfer the water to the biomonitor silicone tubes (VWR, USA) were used. The wastewater was pre-cooled to 16 ± 1 °C.

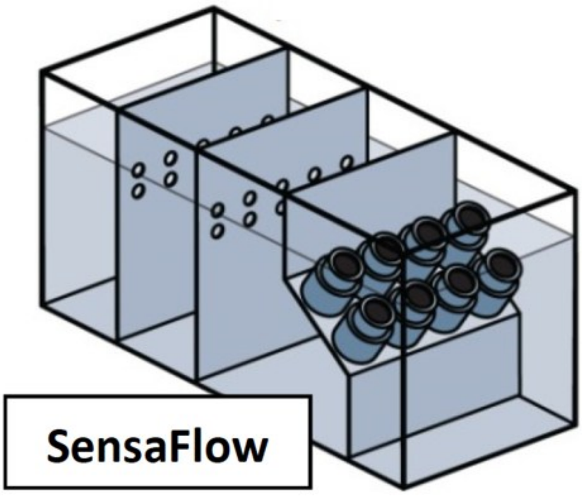
SensaFlow

A

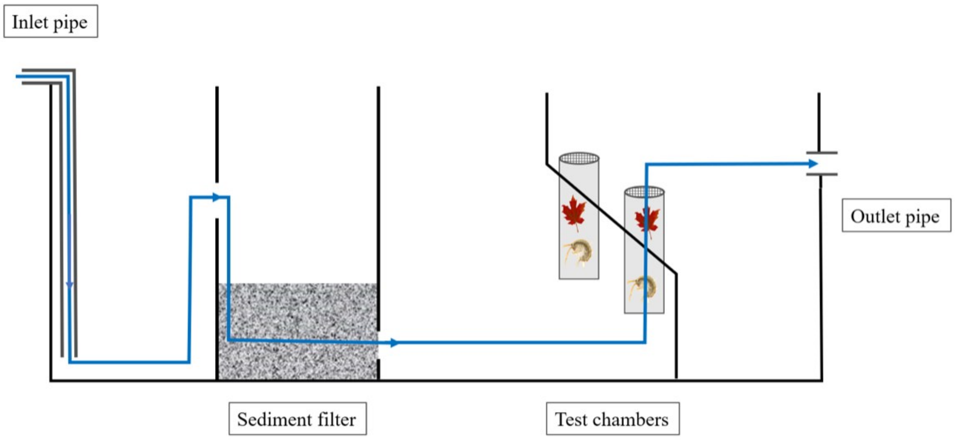
Inlet pipe
Outlet pipe
Sediment filter
Test chambers

B

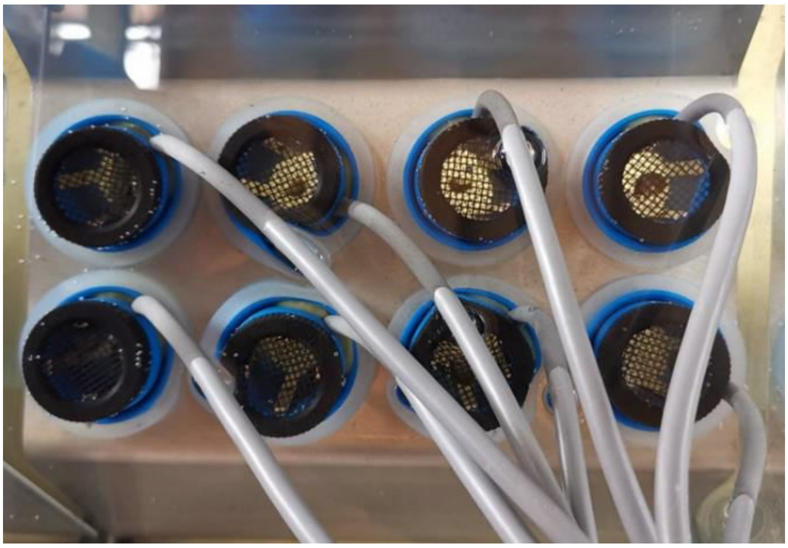

C

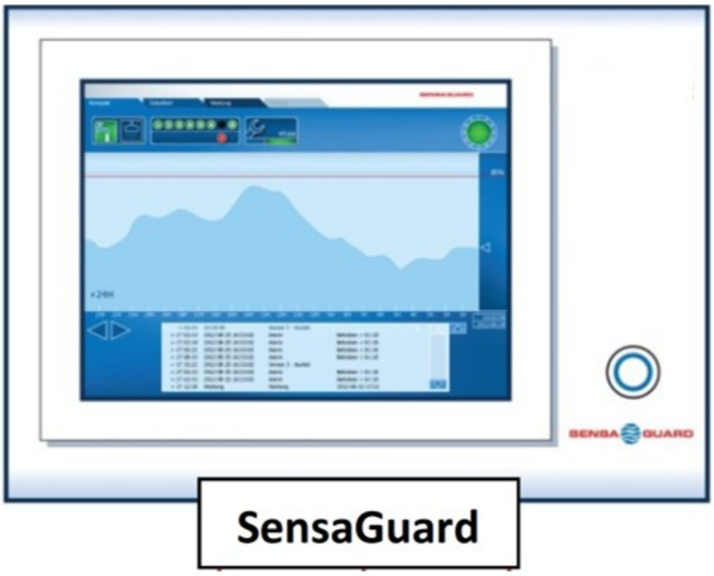

D
Figure S6. A. Sensaflow compartment in three-dimensional B. Sensaflow diagram of flow-through C. Test chambers of Sensaguard biomonitor D. Computer unit of Sensaguard

S6 Technical Information on Membrane System

Monitoring chemical water quality with biological test systems requires a largely germ-free wastewater matrix that ensures continuous and maintenance-free operation of biomonitoring-systems: Fungal spores such as bacteria can have an undesirable effect on the test systems. To ensure this, the already pre-treated wastewater must be additionally filtered. This can be efficiently achieved by using ultrafiltration with pores between 0.1 and 0.01 µm. For this purpose, a membrane filter unit with plate membranes was built to provide sufficient filtered wastewater (permeate) to the test systems

The module block is formed out of several flat-sheet membrane plates attached to an aeration system producing medium-size bubbles. The gas-liquid mixture ascending in between the plates enables to clean the membrane surface[8]. The membrane area was 7 m^2. The filtration/backflush/relaxation cycles were 8 min / 2 min / 2 min and were achieved by a peristaltic pump (MCP Standard, Ismatec, Wertheim, Germany). A membrane air flow rate of 5 m^3/h was created using a rotary vane compressor (V-DTE 6, Elmo Rietschle, Davidson, USA.

The filtration module was equipped with a data acquisition system that allowed to monitor the membrane performance (transmembrane pressure, filtration flow rate and temperature). The permeate pool was aerated (compactON 2100, EHEIM, Deizisau, Germany) and cooled (TK 500, Tecoonline, Ravenna, Italy) to 17 °C. In the permeate pool, temperature, dissolved oxygen and conductivity were measured at 5-min intervals by a multi-parameter portable meter (Multi 3430, WTW, Weilheim, Germany). The treated and filtered wastewater was distributed continuously to the individual BEWS from the pool by internal and pumping systems. For the Algae Toximeter (6 L/h) and the DaphTox II (3 L/h) internal pumps were used. An external pump (505 S, Watson Marlow, Rommerskirchen, Germany) with a flow rate set to 36 L/h provided the Sensaguard unit with wastewater.

Set-up

The filtration system consists of 3 modules on two platforms (Figure S7). Modules I and II are located on the first platform. Module I consists of the control system and is physically separated from Module II with the permeate tank. Module III is located on the second platform. It consists of the membrane filtration unit, which is located in the filtration tank. In addition, both the wastewater and permeate pumps are located on Module III. All the elements required to ensure filtration operation are discussed in detail in the following section.

Pumping of biologically treated wastewater from the outlet of a secondary clarifier

In order to fill the filtration tank (600 L) (E002) with treated wastewater, a pump (M004) is set up or hydrostatic pressure was used, which transports the water from the secondary clarifier of the WWTP.

Pumping the permeate through the membrane

In order to pump the permeate through the membrane filter unit (E001), another pump (M003) is required, which must ensure a transmembrane flow (flux) of up to 100 L/h in order to provide sufficient water for the BEWS (T001-3). The membrane is sensitive to dryness and can suffer

irreversible damage if left "waterless". To prevent this, the level in the tank is monitored by a pressure sensor (B006) which automatically stops the operation of the membrane filter unit if the fill level is too low. The membrane filter unit is formed out of several flat-sheet membrane plates attached to an aeration system. The membrane area is 7 m^2. The filtration/backflush/relaxation cycles were set to 8 min / 2 min / 2 min and were achieved by a peristaltic pump (MCP Standard, Ismatec, Wertheim, Germany).

The flow is monitored here by a flow sensor (B005). To prevent air from accumulating in the permeate line and influencing the pressure measurement (B006), a vent (Y010) is positioned in the connection between the permeate pump and the membrane filter unit.

Monitoring the transmembrane pressure of the membrane

When permeate is conveyed across the membrane, the pressure acting on the entire membrane surface must be monitored by a pressure sensor (B006). Increasing pressure across the membrane and decreasing flow indicate that wastewater matrix components on the membrane surface are impeding membrane flow. If the pressure is to high there is a risk of damaging the membrane plates irreversibly. To prevent this, chemical cleaning of the membrane filter unit is required.

Aeration of the membrane filter unit

The membrane filter unit (E001) consists of the membrane filter plates and an aerator. The aerator is continuously supplied with compressed air from a blower (M005). This causes coarse air bubbles to flow continuously between the vertically arranged membrane plates, reducing the deposition and accumulation of particles from the wastewater matrix onto the membrane surface. A membrane air flow rate of 5 m3/h was created using a rotary vane compressor (V-DTE 6, Elmo Rietschle, Davidson, USA).

Possibility of backwashing and chemical cleaning of the membrane.

By periodically backwashing the membrane filter unit, between the regular pumping of the permeate, the membrane surface can be kept largely free of particles from the wastewater matrix to ensure the desired membrane flux over a longer period of time. For this purpose, a reversible pump (M003) can be used to backwash the membrane filter unit with the permeate that was previously pumped across the membrane. The permeate tank (60 L) (E003) serves here as a pre-tank for the permeate to be backwashed. The permeate pool is aerated (compactON 2100, EHEIM, Deizisau, Germany) and cooled (TK 500, Tecoonline, Ravenna, Italy) to 17 °C. In the permeate pool, temperature, dissolved oxygen and conductivity is measured at 5-min intervals by a multi-parameter portable meter (Multi 3430, WTW, Weilheim, Germany). During chemical cleaning, the wastewater in the filtration tank is first completely drained via the drain valve (Y005), filled with water and added optionally with sodium hypochlorite (NaOCl) or citric acid. After incubation for 5-6 h, the solution is transferred across the membrane to the permeate tank and discharged via the overflow (Y007) until the filtration tank is again filled exclusively with wastewater from the secondary clarifier. Details on membrane system components are listed in Table S2.

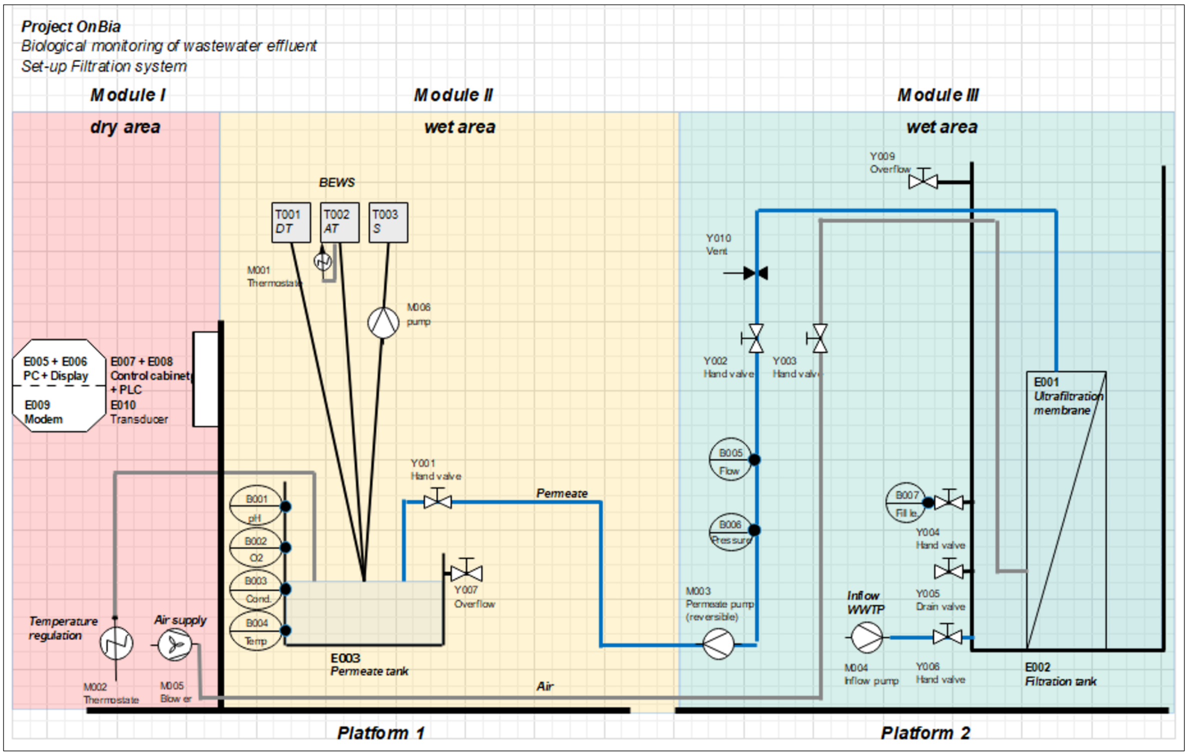

Figure S7. P&ID diagram of modular membrane system

Table S2. List of membrane system components

**Abbr.**	Component	Type	Description	Vendor
**E001**	Ultrafiltration Membrane	IPC-7	Membrane unit with aerator	*Blue Foot Membranes*
**E002**	Filtration tank	-	KVA-container	*Kubaco*
**E003**	Permeate container	-	-	-
**E004**	Backflush container	-	-	-
**E005**	PC	OptiPlex3060	PC	*Dell*
**E006**	Monitor	P2319H	Screen for PC	*Dell*
**E007**	Control cabinet	-	Access to measuring transducer and PLC	-
**E008**	PLC	750-8212	Programmable logic control	*Wago*
**E009**	Modem	SCR-L200	Access to internet	*InSys*
**E010**	Transducer	CM448	*Liquiline* - 8-Channel-transducer	*Endress+Hausser*

**M001**	Thermostat	FN25	Cooler for Algae Toximeter	*Julabo*
**M002**	Thermostat	TK-500	Cooler for permeate tank	*Teco*
**M003**	Permeate pump	ISM 404	Reversible peristaltic pump	*Ismatec*
**M004**	Wastewater pump	DULCOflex	Peristaltic pump	*Prominent*
**M005**	Compressor	V-DTE 6	Aeration of membrane unit	*Elmo Rietschle*
**M006**	Pump	VC-380	Peristaltic pump for Sensaguard	*Ismatec*
**B001**	pH - Sensor	CPS11D	*Orbisint* – pH und temperature measurement	*Endress+Hausser*
**B002**	Oxygen-Sensor	COS61	*Oxymax* –Measurement of oxygen	*Endress+Hausser*
**B003**	Conductivity-Sensor	DMA20	*Picomag* – Measurement of conductivity	*Endress+Hausser*
**B004**	Flow-Sensor	DMA20	*Picomag* – Measurement of flow rate	*Endress+Hausser*
**B005**	Pressure-Sensor	PMC11	*Cerabar* – Measurement of transmembrane pressure	*Endress+Hausser*
**B006**	Pressure-Sensor	PMC11	*Cerabar* – Measurement of fill level	*Endress+Hausser*
**B007**	Temperature-Sensor	CPS11D	*Orbisint* – Temperature measurement	*Endress+Hausser*

S7 Behavioral assessment

S8 Extended Results of BEWS at Municipal WWTP

The following section includes the results from all behavioral parameters of the BEWS during the WWTP measurements and the systems own temperature measurement. For measurements of physicochemical parameters during the experiments see Excel-SI (Table X1).

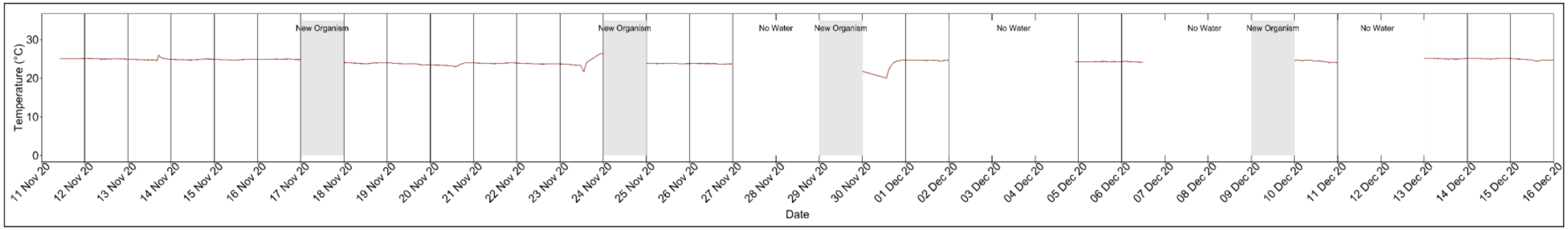

Figure S8. Algae Toximeter.: Temperature (°C) indicated by red lines.

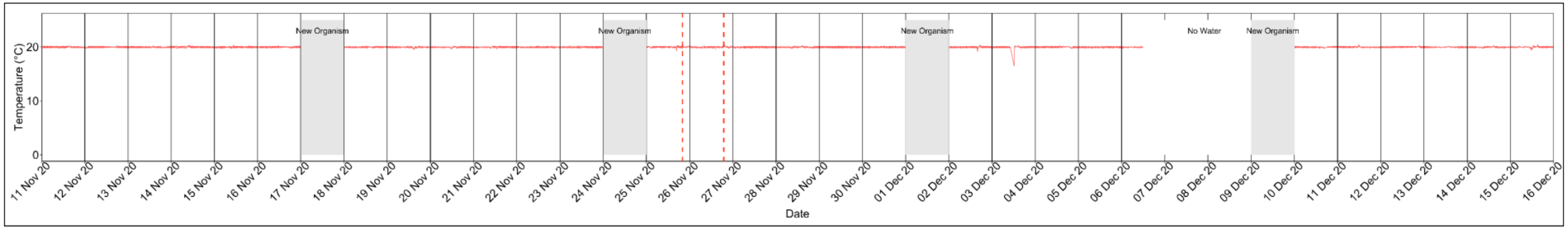

A

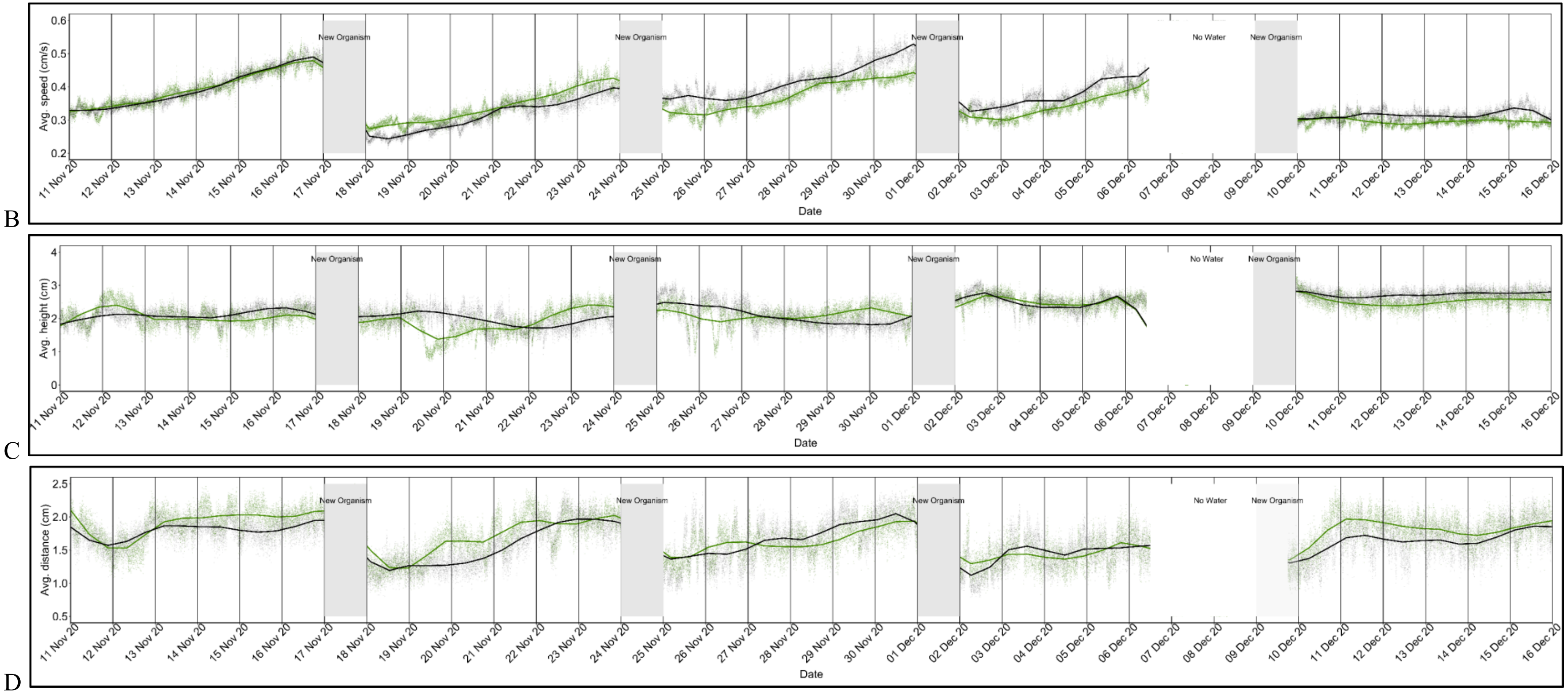

Figure S9. DaphTox II. A: Temperature (°C) indicated by horizontal red lines. Vertical dashed red lines indicate alarms triggered. B: Average speed (cm/s) - Green and black lines represent swimming speed in the test chambers 1 and 2. C: Average height (cm) - Green and black lines represent swimming height in the test chambers 1 and 2. D: Average distance (cm) - Green and black lines represent swimming distance in the test chambers 1 and 2.

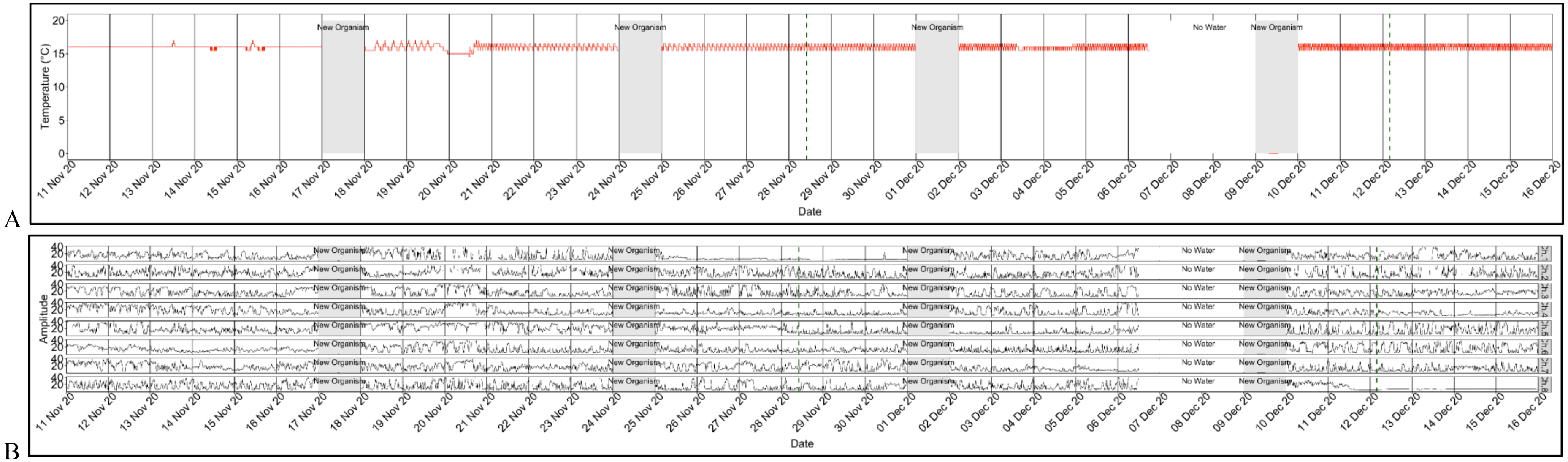

Figure S10. Sensaguard. A: Temperature (°C) indicated by horizontal red lines. Vertical dashed green lines indicate alarms triggered. B: Single amplitudes of all 8 individuals indicated by black lines.

S9 Comparison of Behavioral Activity of *G. pulex* with and without mortality

During week five, no alarms occurred in the DaphTox II and very little variability in the toxic index was displayed. The Sensaguard triggered an alarm on December 13, related to the death of an individual on December 12 (Figure 2C in the main manuscript). The seven other organisms in the system showed no deviation from normal behavior. Since the death of an individual one day prior to the alarm event contributed to the alarm signal of the Sensaguard, it was decided to evaluate 24 h before the alarm event for all possible causative possibilities. The physiochemical parameters were checked and only the ammonia level in the effluent increased slightly from 0.4 to 1.1 mg/L from December 12 to 13. This was still below the effective concentration level discussed in the previous section. As the concentrations of target compounds were rather low during this period, an analysis of non-target chemicals was performed. The non-target analysis during the Sensaguard alarm event revealed the industrial chemical tributyl phosphate, an organophosphorus compound, at 0.015 mg/L. After reviewing the time of occurrence and quantification of this compound and comparing toxicity levels for *D. magna* (24 h-LC50 = 5.48 mg/L)[9] and *G. psudolimnaeus* (96 h-EC50 = 1.7 mg/L)[10], it was decided that no laboratory testing was conducted as this compound occurred after the death of the individual in a concentration factor more than 100 times less than the toxicity value in literature. Testing the hypothesis that the mortality could have been due to natural causes like age, the data were re-evaluated after excluding the dead individual from the analysis, which indicated that the alarm parameter would not have been exceeded if the individual had been alive (Figure S11).

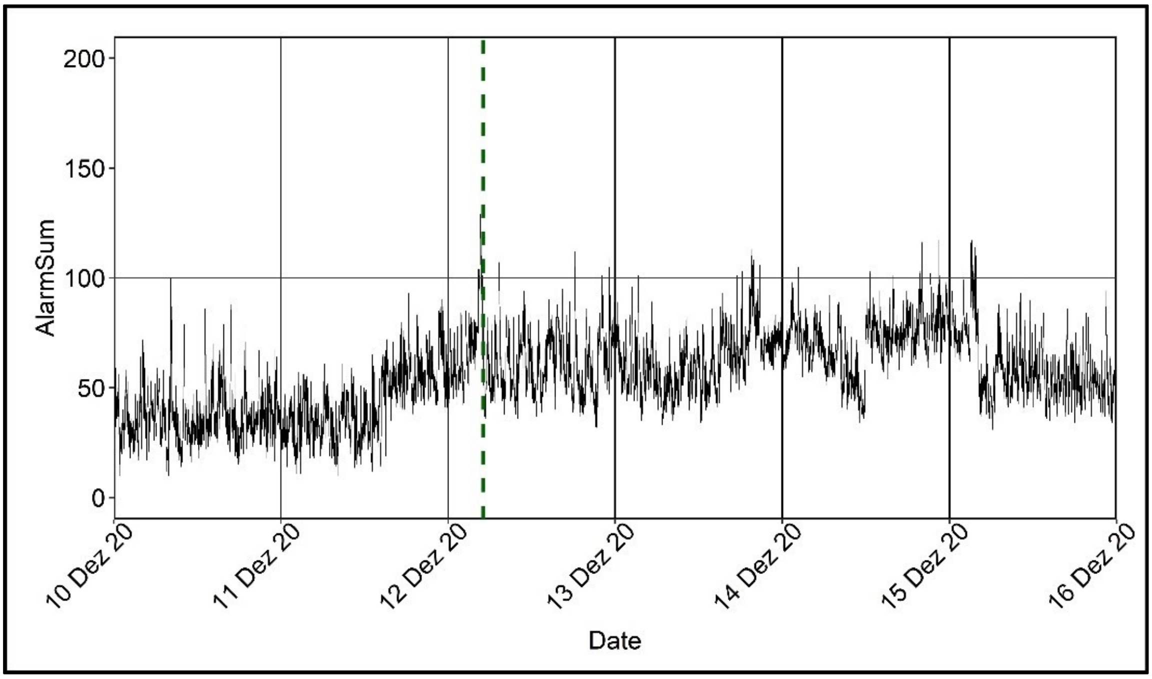

A

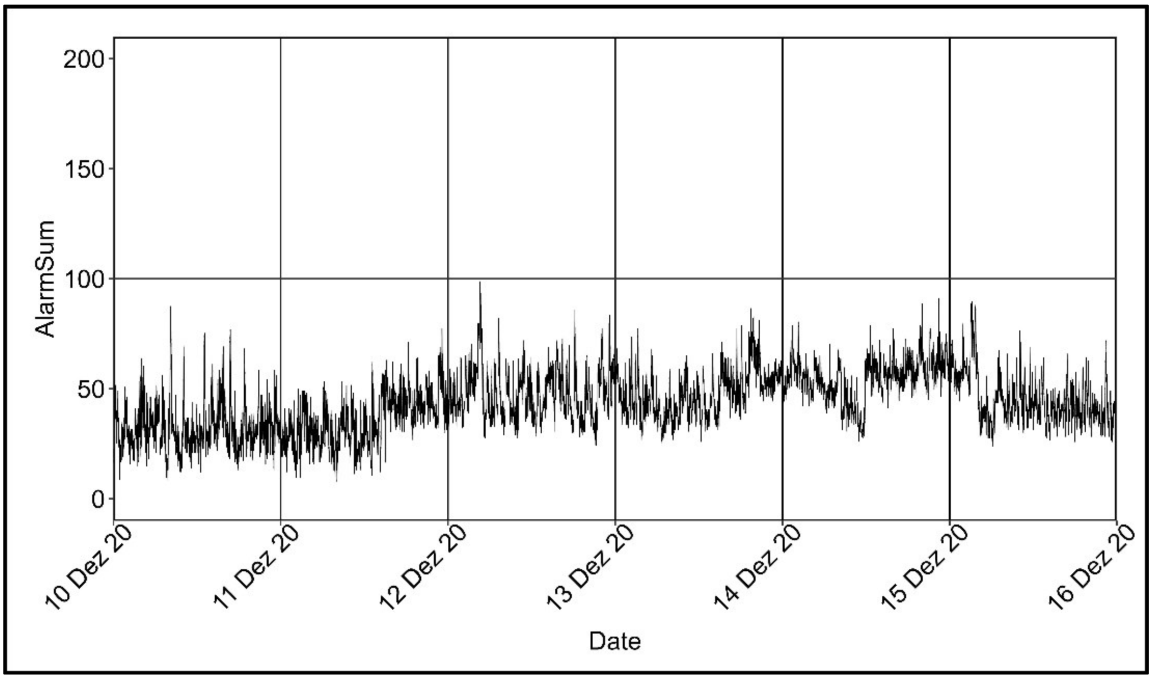

B

Figure S11. Sensaguard - Alarmsum of *Gammarus pulex* behavior during fifth week of the WWTP monitoring. A: Black lines display the average behavioral activity of all 8 organisms. Vertical dashed green line shows alarm triggered. Horizontal black line shows alarm threshold B: Black lines display the average behavioral activity of 7 individuals.

S10 Extended Results of BEWS Laboratory Tests with Carbofuran

Experimental setup

Six experiments were conducted in total with wastewater from pilot-scale WWTP. The pilot-scale WWTP (Eawag, Switzerland) receives municipal wastewater and treats approximately 200 population equivalents (<72 m^3/d inflow). The mechanically treated wastewater is passing through a sedimentation stage, followed by denitrification and nitrification. Subsequently, it is collected in the secondary clarification tank (10.6 m^3). Wastewater from the secondary clarifier was directed into a filtration tank, where a backflushable ultrafiltration membrane module (IPC 7, PVDF, pore size: 0.08 µm, BlueFootMembranes, Belgium) was placed (see chapter S3). A constant permeate flux of 11.42 L/m^2h was provided from the membrane stack and directed into an aerated plexiglass container (permeate pool), where the permeate was conditioned to 17 °C. The treated and filtered wastewater was distributed continuously to the individual BEWS from the pool by pumping systems. Additional information on the membrane filter unit can be found in S3. The first experiment served as a baseline for the DaphTox II and the Sensaguard and was conducted with treated effluent only for 7 days. To simulate the exposure, tanks (100L) with treated wastewater were prepared without carbofuran and switched every 24 h for the baseline week. Subsequently, four concentrations of carbofuran with a spacing factor of 3 were added once a week for 24 h to the treated wastewater exposed to the BEWS. In the final experiment, five concentrations of carbofuran were spiked every 24 h within one week after two days of acclimation to the treated effluent exposed to the BEWS. For information on physicochemical data during the verification tests see Excel-SI (Table X2).

DaphTox II

The behavioral parameters “average speed”, “fractal dimensions”, “average height” and “average distance” of the DaphTox II contribute to the calculation of the toxic index which represents the live state of the daphnia which are exposed to the monitored water. In this section these parameters and the temperature are presented as supporting information for the baseline and all treatments with carbofuran (Figure S12-14).

Baseline

First Carbofuran Concentration

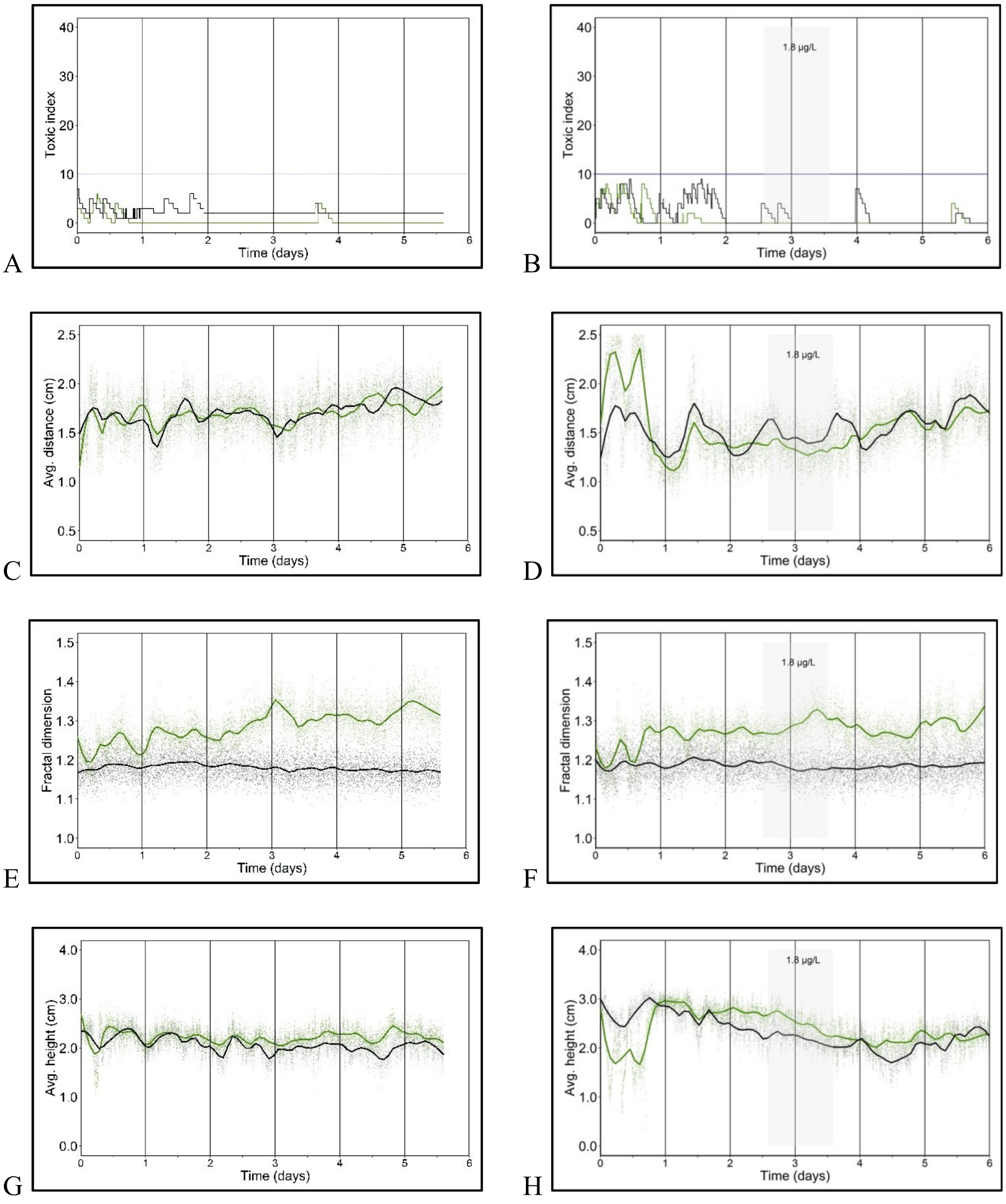

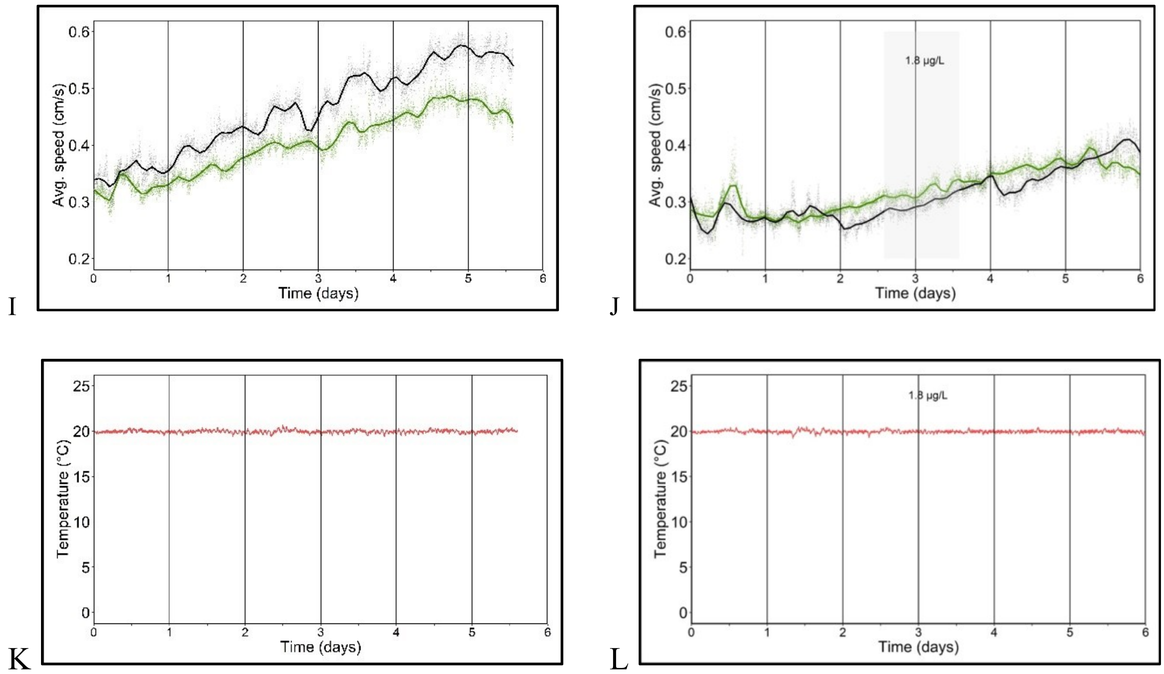

Figure S12. DaphTox II - Calculated parameters during the experimental baseline week with exposure of *D. magna* with wastewater (A; C; E; G; I) and exposure of first carbofuran concentration (1.8 µg/L) control (B; D; F; H; J). Experimental period of 6 days is displayed. Black line and green line represent behavioral activity in the test chambers 1 and 2. A-B: toxic index of *D. magna*. Vertical blue line indicates alarm threshold C-D: Average Distance of *D. magna*. E-F: Fractal dimension of *D. magna*. G-H: Average height (cm) of D. magna. I-J: Average speed (cm/s) of *D. magna*. L-L: Temperature (°C).

Second Carbofuran Concentration

Third Carbofuran Concentration

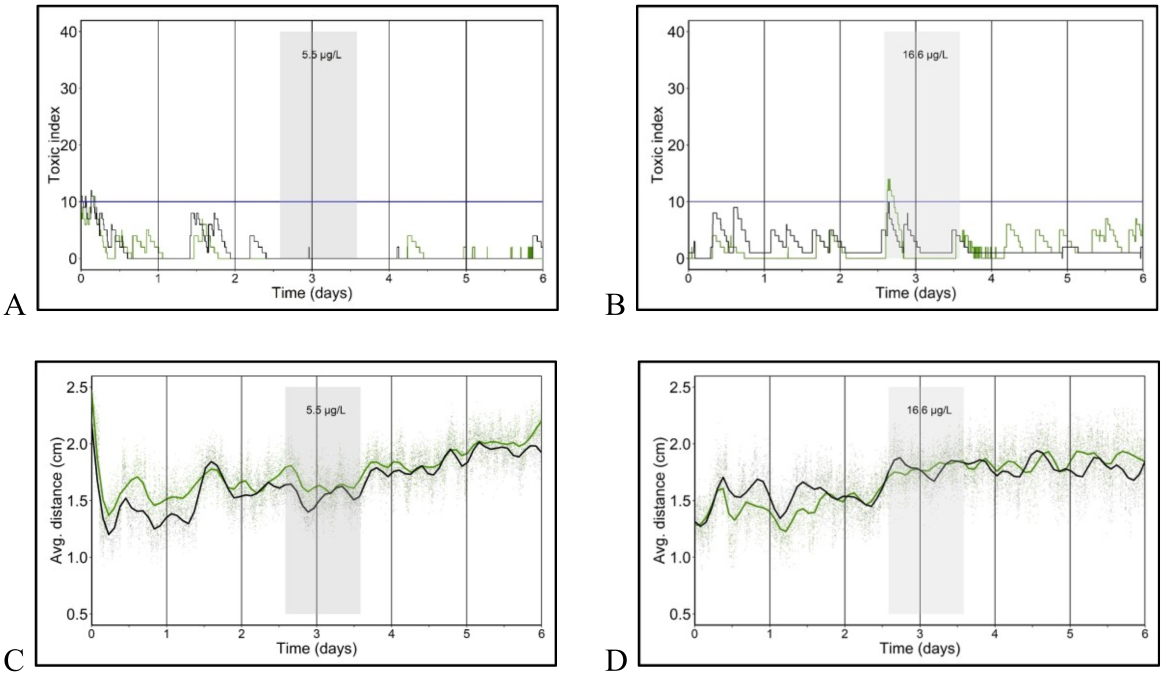

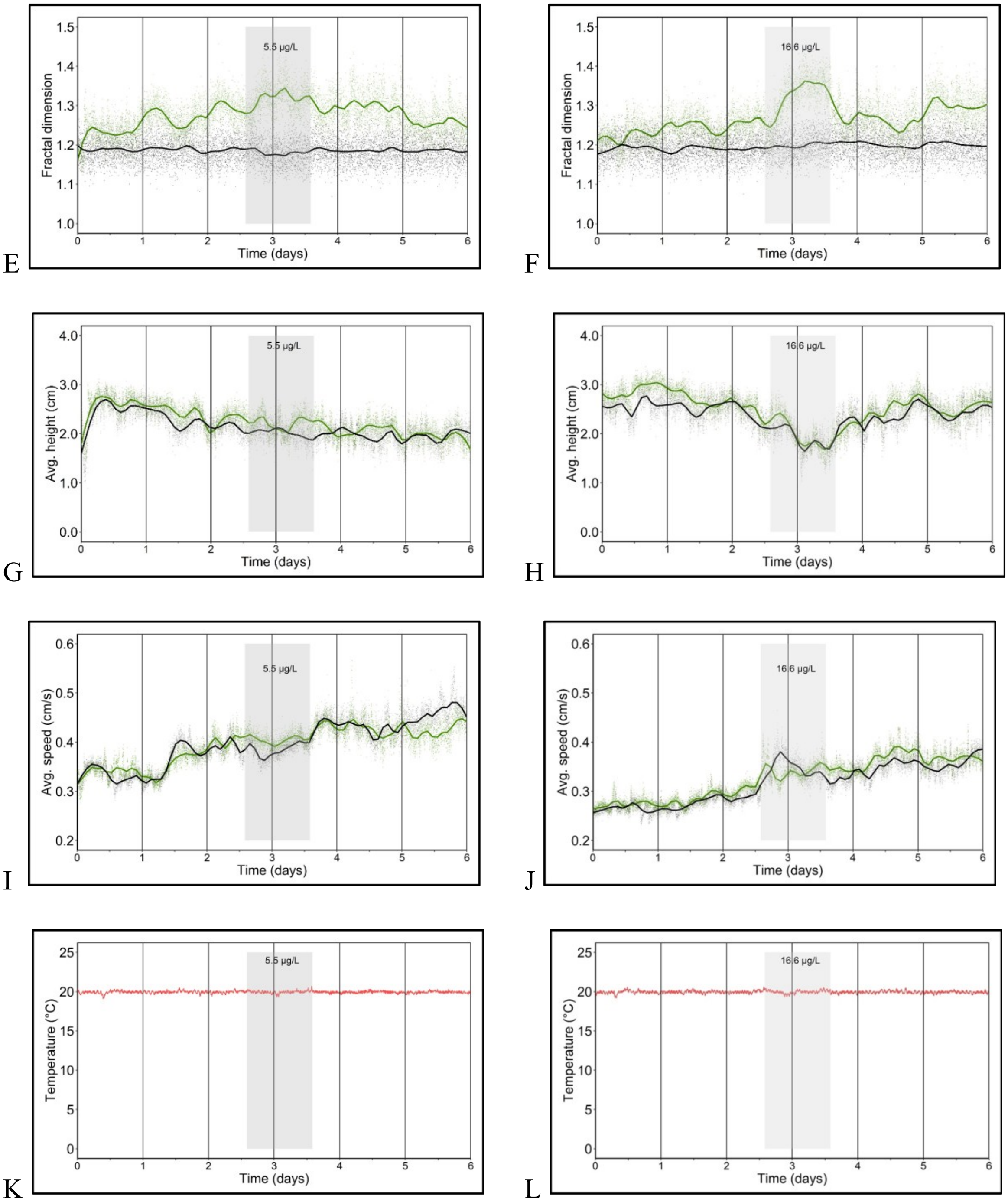

Figure S13. DaphTox II - Calculated parameters during the experimental baseline week with exposure of *D. magna* with second (5.5 µg/L) (A; C; E; G; I; K) and third carbofuran concentration (16.6 µg/L) (B; D; F; H; J; L). Experimental period of 6 days is displayed. Black line and green line represent behavioral activity in the test chambers 1 and 2. A-B: toxic index of *D. magna*. Vertical blue line indicates alarm threshold C-D: Average Distance of D. magna. E-F: Fractal dimension of D. magna. G-H: Average height (cm) of *D. magna*. I-J: Average speed (cm/s) of *D. magna*. L-L: Temperature (°C).

Fourth Carbofuran Concentration

All Carbofuran Concentrations

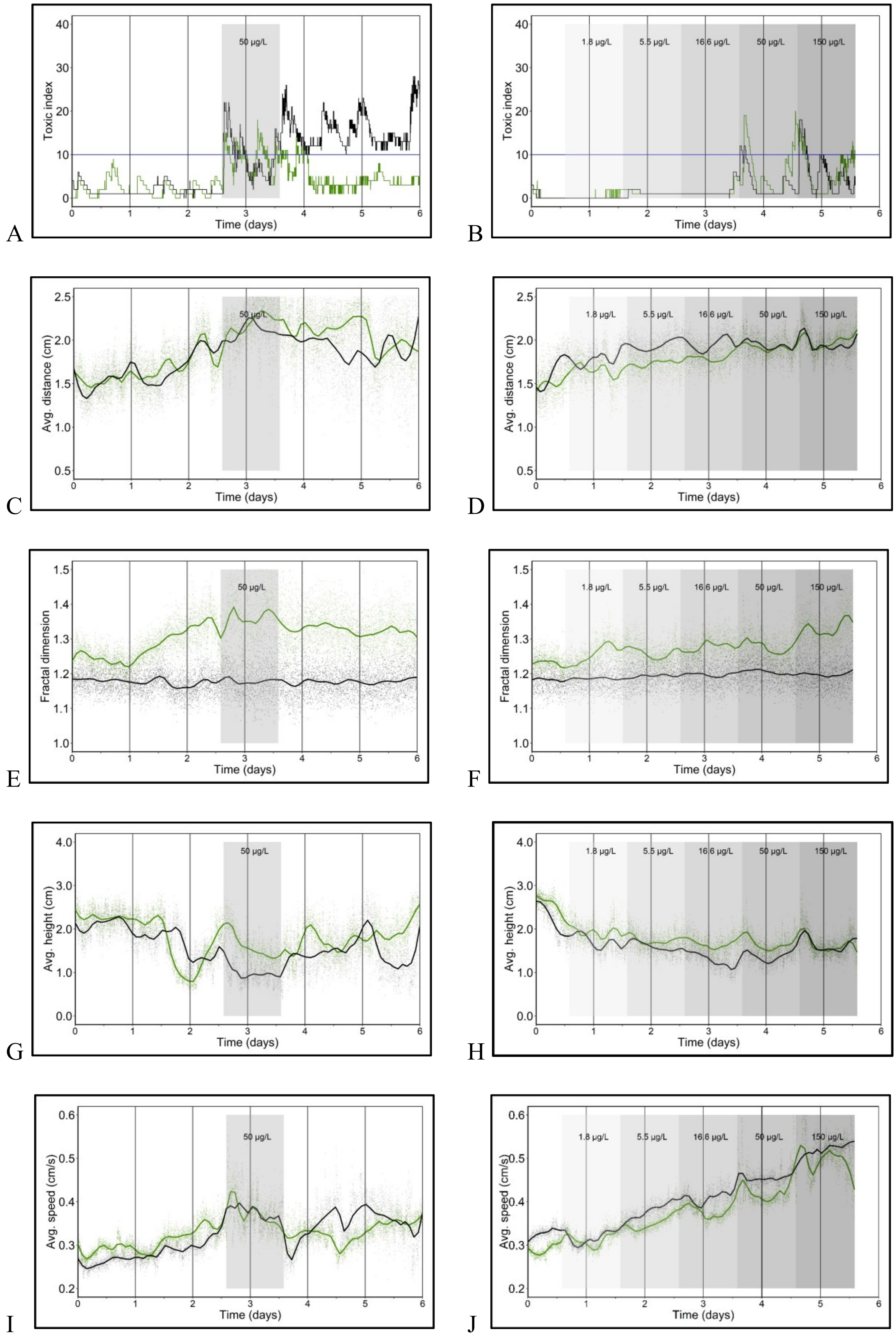

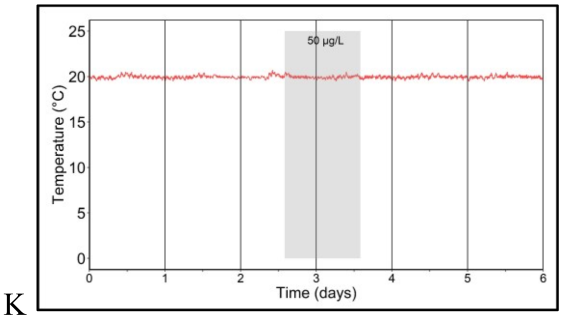

K

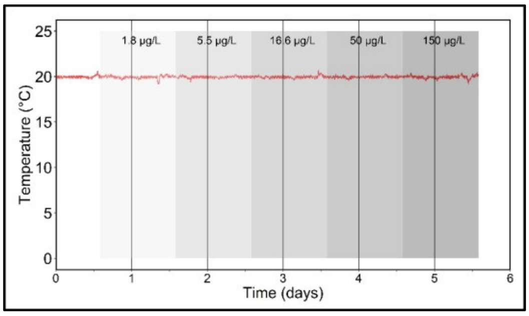

L

Figure S14. DaphTox II - Calculated parameters during the experimental baseline week with exposure of *D. magna* with fourth (50 µg/L) (A; C; E; G; I; K) and all carbofuran concentrations in one week (B; D; F; H; J; L). Experimental period of 6 days is displayed. Black line and green line represent behavioral activity in the test chambers 1 and 2. A-B: toxic index of D. magna. Vertical blue line indicates alarm threshold C-D: Average Distance of D. magna. E-F: Fractal dimension of D. magna. G-H: Average height (cm) of *D. magna*. I-J: Average speed (cm/s) of D. magna. L-L: Temperature (°C).

Sensaguard

Analysis of the movement patterns of *G. pulex* by examining individual signals from the measurement chambers and applying multivariate changepoint analysis revealed differences in behavior between the individual treatments. In the following, the systems own parameter "AlarmSum", the individual movement patterns and the multivariate changepoint analysis of the average distance moved parameter of each carbofuran treatment are displayed over 6 days (Figures S15-17).

Baseline

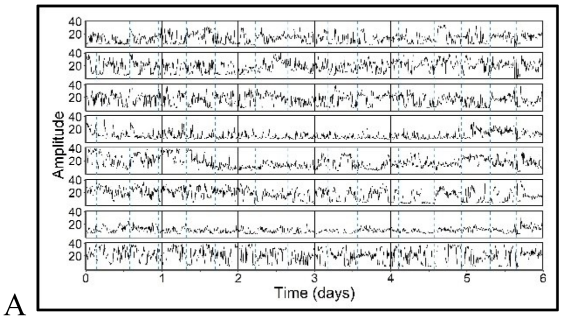

A

First Carbofuran Concentration

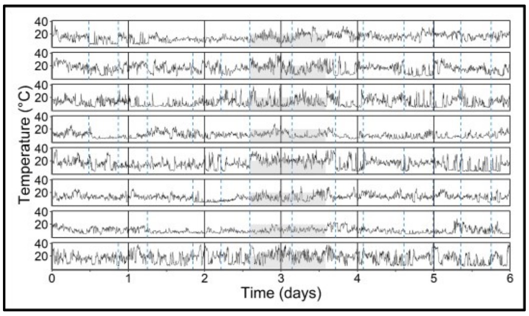

B

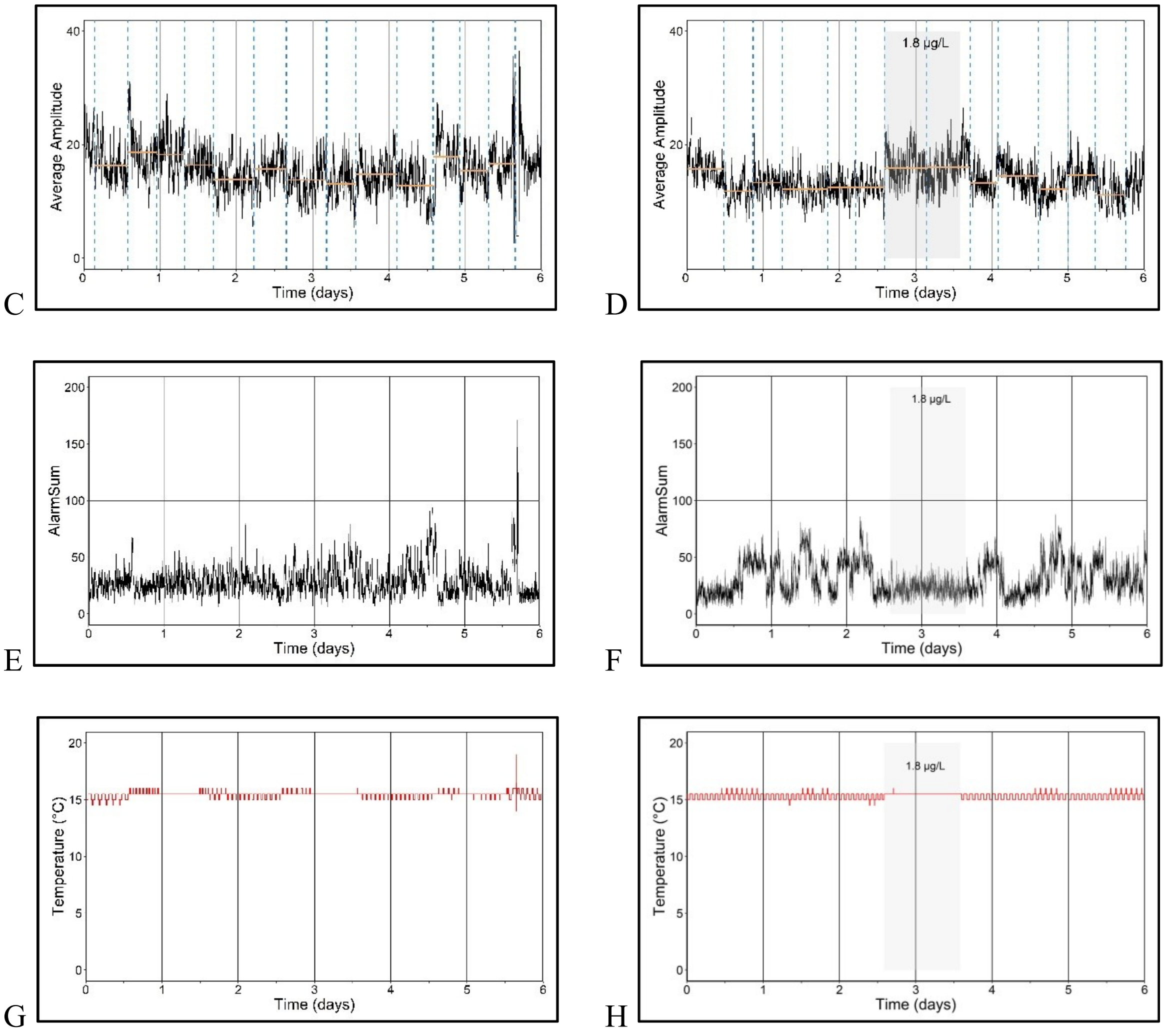

Figure S15**.** Sensaguard - Amplitudes of the individual organisms (A, B), average amplitude (C, D) (including changepoints), AlarmSum (E, F) of *G. pulex* behavior and temperature (G, H) during the experimental week with wastewater (A, C, E, G) and first carbofuran concentration (B, D, F, H): A, B: black lines indicate the behavioral activity, vertical blue dotted lines indicate changepoints, where changes in single activity were detected in the multivariate change point analysis. C, D: Calculated average amplitude of 8 *G. pulex*: Vertical blued dotted lines display the time-points detected in the multivariate change point analysis; orange lines show the mean of each segment whose length is determined by the blue dotted lines. E-F: Calculated AlarmSum parameter of 8 *G. pulex*. Horizontal black line shows alarm threshold. G, H: temperature during the experimental period.

Second Carbofuran Concentration

Third Carbofuran Concentration

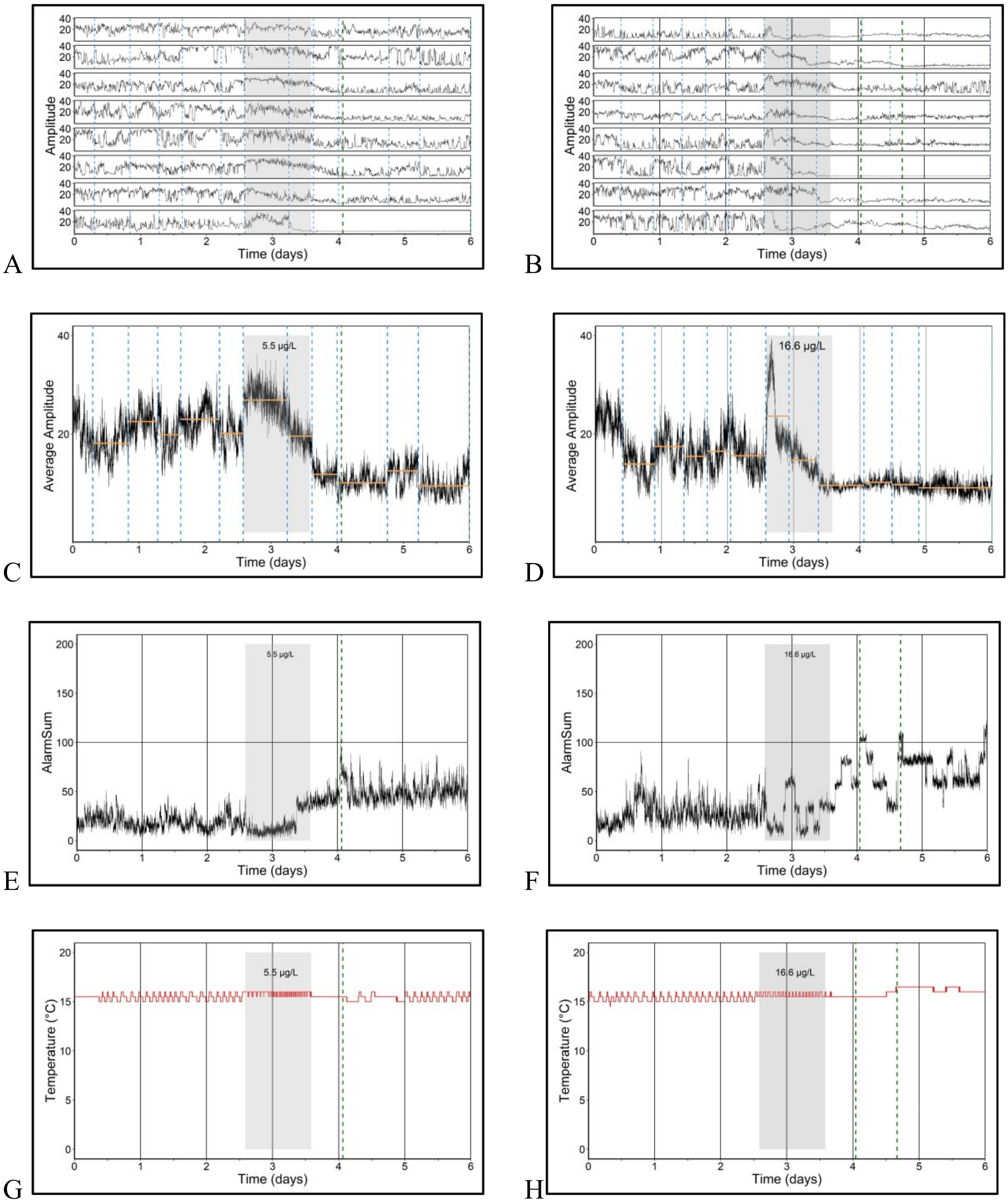

Figure S16. Sensaguard - Amplitudes of the individual organisms (A, B), average amplitude (C, D) (including changepoints), AlarmSum (E, F) of *G.s pulex* behavior and temperature (G, H) during the experimental week with second (A, C, E, G) and third carbofuran concentration (B, D, F, H): A,B: black lines indicate the behavioral activity, vertical blue dotted lines indicate changepoints, where changes in single activity were detected in the multivariate change point analysis. C, D: Calculated average amplitude of 8 *G. pulex*: Vertical blued dotted lines display the time-points detected in the multivariate change point analysis; orange lines show the mean of each segment whose length is determined by the blue dotted lines. E-F: Calculated AlarmSum parameter of 8 *G. pulex*. Horizontal black line shows alarm threshold. G, H: temperature during the experimental period

Fourth Carbofuran Concentration

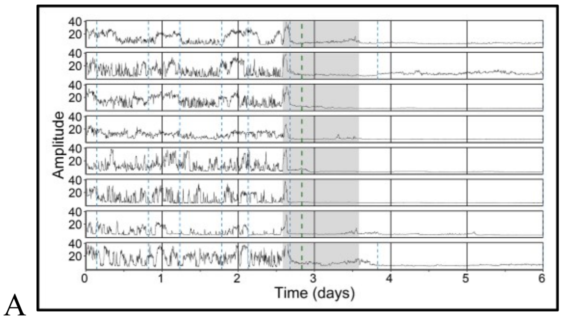

A

All Carbofuran Concentrations

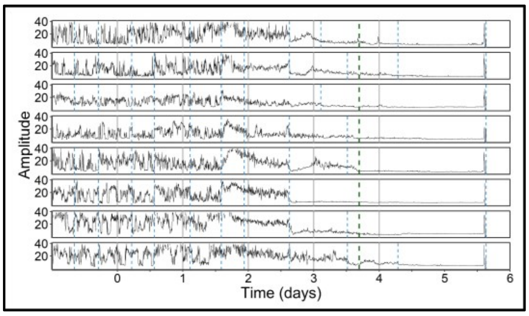

B

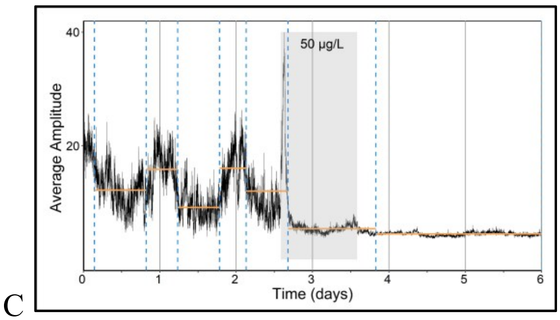

C

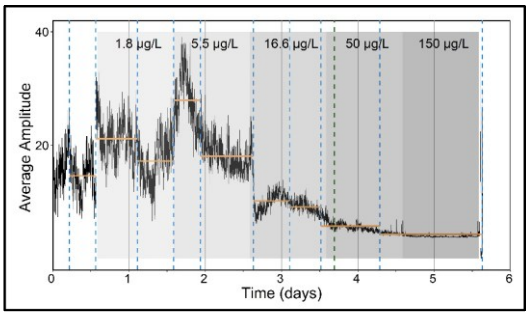

D

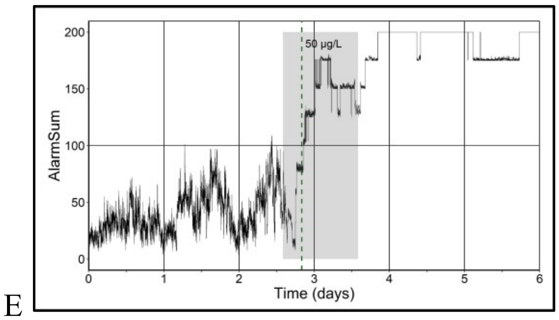

E

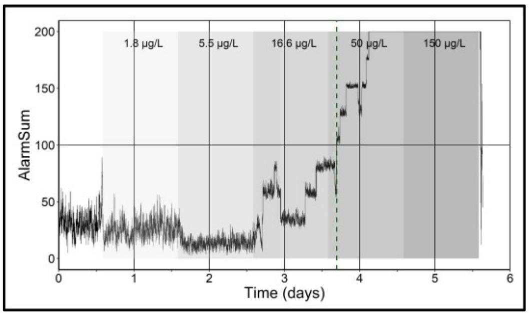

F

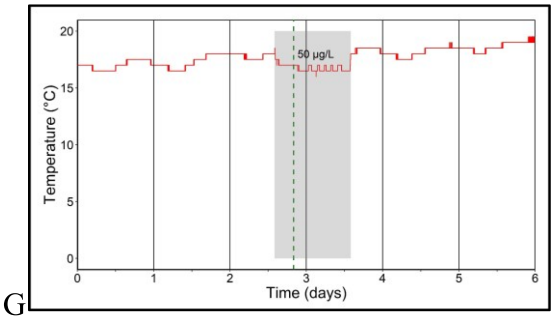

G

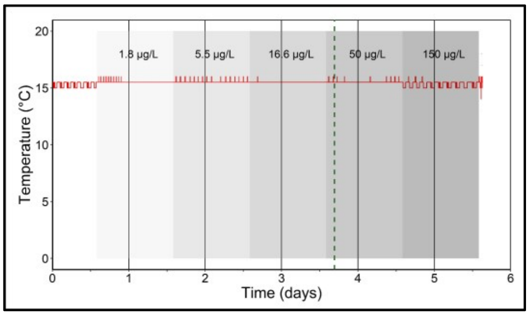

H

Figure S17. Sensaguard - Amplitudes of the individual organisms (A, B), average amplitude (C, D) (including changepoints), AlarmSum (E, F) of *G. pulex* behavior and temperature (G, H) during the experimental week fourth concentration (A, C, E, G) and all carbofuran concentration in one week (B, D, F, H): A,B: black lines indicate the behavioral activity, vertical blue dotted lines indicate changepoints, where changes in single activity were detected in the multivariate change point analysis. C, D: Calculated average amplitude of 8 *G. pulex*: Vertical blued dotted lines display the time-points detected in the multivariate change point analysis; orange lines show the mean of each segment whose length is determined by the blue dotted lines. E-F: Calculated AlarmSum parameter of 8 *G. pulex*. Horizontal black line shows alarm threshold. G, H: temperature during the experimental period

S11 Statistics

S12 Statistical Methods for Biomonitoring Data

Multivariate Changepoint Analysis of Sensaguard Data

All data was analyzed and visualized using the statistical software R, version 4.2.0 for Windows[11]. The behavior analysis during each of the one-week experiments focused on pattern analysis. Changes in patterns for the eight individual organisms were studied by applying multivariate changepoint detection methodology. In this paper, the term “changepoint” is defined as a time-point where a change in the mean occurs along the one-week experiment. We used the smop[12] package in R to identify the changepoints that occurred for the eight individual organisms. Due to the intrinsic variability between the eight organisms, the pattern analysis focus on changepoints, that not necessarily occurred simultaneously in all eight organisms, but in a subset of them. For this purpose, the smop package uses the approximation of the SMOP

algorithm, details on the algorithm and changepoint detection with subsets can be found in Pickering (2015)[13].

To reduce the computational time in the multivariate changepoint analysis, the raw amplitude values (one per second) in the time-series were grouped in five min average intervals. For a simple visualization, the individual detected changepoints were transferred to the average amplitude of the eight organisms – this time with a resolution of one-minute interval. Based on these changepoints, the average amplitude was divided into segments and an orange horizontal line representing the average amplitude of each segment was added. This graph allows a straightforward observation of the differences in the average amplitude between the segments determined by the changepoints.

Simquant Analysis of Sensaguard Data

For assessing whether there is a pattern in the behavior of the Gammarids' movement, the daily rhythms have been assessed. In order to see the general structure, it has been decided to use hourly averages as a basis. A linear random effects model has been fitted with the hourly averages as fixed effect, and the individuals' hourly behavior as random effects. The effect of the "hour" was treated as fixed effect, with potential random effects due to the individual animals, the days, a modified pattern of the hourly behavior due to the day or due to the individual, an effect per day per individual.

For assessing the difference between hours, we use the emmeans[14] package of R which allows to estimate marginal means and also yields adjusted p-values for the multiple comparisons, as well as visualizations. For comparison, we have also calculated the compact letter display using multcomp[15] package.

For determining the limits of the confidence bands, a bootstrap procedure has been used where the variances obtained from the linear mixed models served as a basis. 10'000 "studies", each comprising 6-day time series for 8 individuals, have been simulated after collection 7 weeks of

baseline information (Figure S18). From the 8 individuals, the average 6-day time series for the "study" has been calculated. The empirical 95% and 99.7% intervals from these simulations serve as the prediction limits, for the behavior of the individuals as well as for the average behavior of 8 individuals stemming from the same "study". There is still some variability between the "days" due to the model. For obtaining a single limit only comprising a 24-h-period, the 6 days within a study have been combined as if they were independent measures. Taking the empirical intervals of these yields a smoothed version of the 6 per-day limits. For the plots, we have used the 95% prediction limits

Baseline Data Sets for Simquant analysis

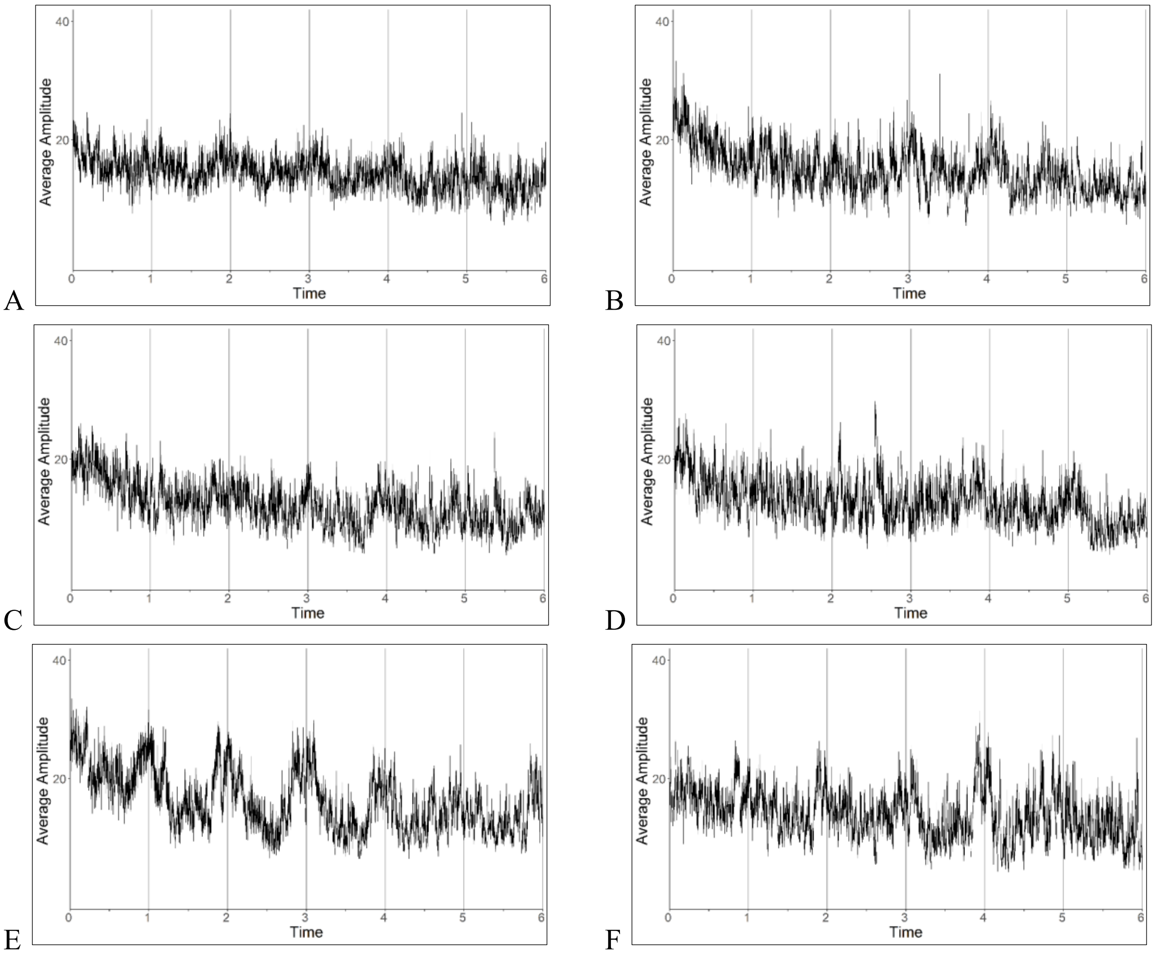

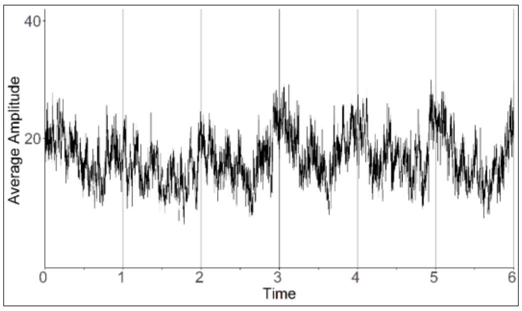

G

Figure S18**.** Sensaguard - Average amplitude (A-G) of *Gammarus pulex* behavior during six baseline weeks. Measured average amplitude of *G. pulex*: Black lines display the average behavioral activity of all 8 organisms

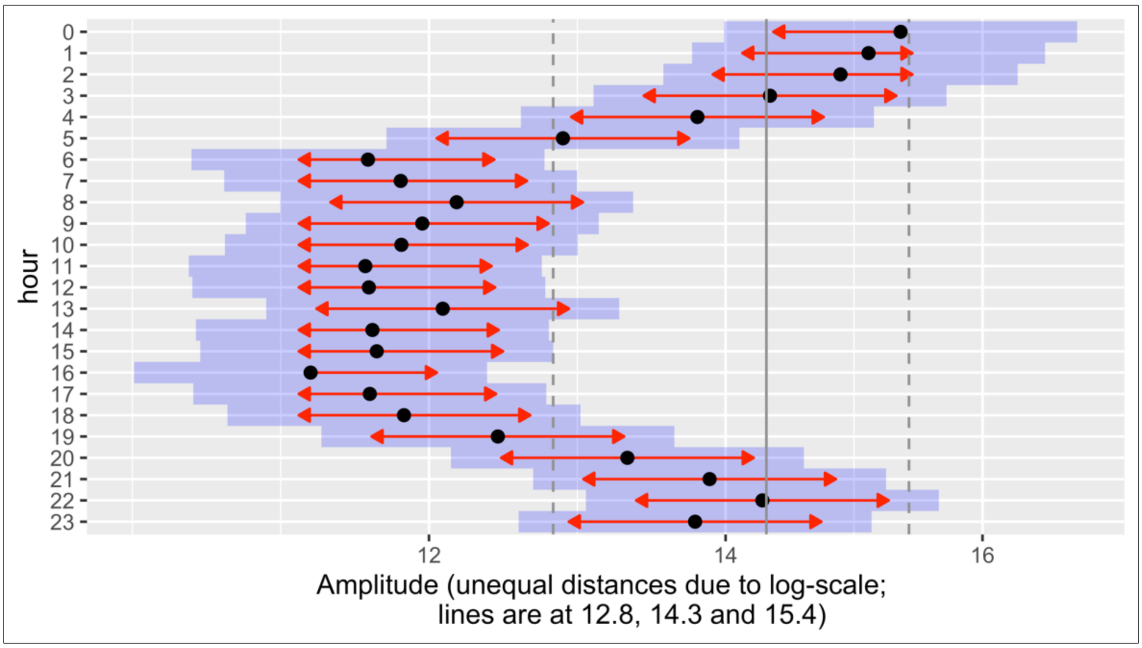

Figure S19. emmeans-plot for the baseline data set. The least activity was between 6 h and 17 h, and the highest between 21 h and 3 h (dashed lines).

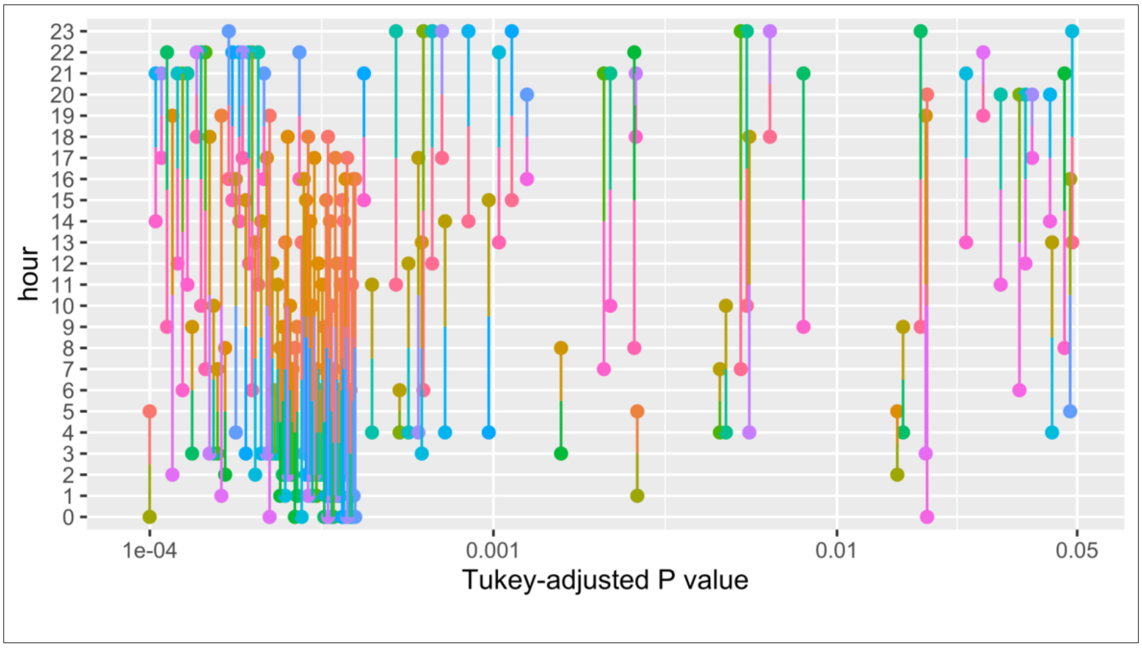

Figure S20. plot of the p-values of the pairwise differences between hours for the simquant analysis

S13 Regression Model to Correlate Behavioral Data with Chemical and Abiotic Information

A Generalized Linear Mixed Model (GLMM) was used in our analysis to investigate the relationship between chemical data or abiotic parameters and organism behavior over time, using the glmmTMB[16] package in R. The model for the analysis considered behavioral data as the response variable, chemical (target and non-target compounds) and abiotic (temperature, oxygen, pH, conductivity and NH_4) data as predictor variables, and individual differences between organisms and the temporal correlation of measurements taken on the same organism over time as random variation. To test the assumptions of the model and to ensure that they were met, we used a Type II analysis of variance, analogous to ANOVA for generalized linear models, to test the null hypothesis that each predictor has no effect on the response variable. The model was used to test the following hypotheses (H1-3) in terms of compound effects:

Table S3. Analysis of Deviance with Type II Wald chi-square test for each predictor's significance correlated to behavioral data in the GLMM

Week 2

H1: Increased behavioral activity of gammarids correlates is attributed 2,4 Dichlorophenol

Predicted variables	chi-square (χ^2)	p-value	significance
Temperature	4.0871	0.0432	*
pH	4.9023	0.0268	*
Oxygen	3.5206	0.0606	.
Conductivity	1.4064	0.2356	
NH4	1.1704	0.2793	
2,4-Dichlorophenol	0.0991	0.7529	

No, the p-value for the predictor "2,4-Dichlorophenol" in the Type II Wald chi-square test is 0.7529, which is greater than the common significance level of 0.05. Therefore, there is no significant correlation between 2,4-Dichlorophenol and gammarid behavior in the model.

Week 3

H2.1: Change in behavioral activity of gammarids correlates is attributed to carbofuran

Predicted variables	chi-square (χ^2)	p-value	significance
Temperature	4.0742	0.0435	*
pH	1.7121	0.1907	
Oxygen	7.1162	0.0076	**
Conductivity	4.4318	0.0352	*
NH4	0.8660	0.3520	
4-Aminoantipyrine	5.9837	0.0144	*
Carbofuran	12.8676	0.0003	***
Lidocaine	1.8278	0.1763	
Xylazine	0.5423	0.4614	

Yes, the p-value for the predictor "Carbofuran" in the Type II Wald chi-square test is 0.00047, which is greater than the significance level of 0.001. Therefore, there is highly significant correlation between carbofuran and gammarid behavior in the model.

H2.2: Change in behavioral activity of daphnia is attributed to carbofuran

Predicted variables	chi-square (	p-value	significance
Temperature	2.5757	0.1085	
pH	1.1973	0.2738	
Oxygen	1.4714	0.2251	
Conductivity	5.3384	0.0208	*
NH4	1.7613	0.1844	
4-Aminoantipyrine	0.7505	0.3863	
Carbofuran	0.0604	0.8058	
Lidocaine	0.2056	0.6502	
Xylazine	11.7596	0.0006	***

No, the p-value for the predictor "Carbofuran" in the Type II Wald chi-square test is 0.8058, which is greater than the common significance level of 0.05. Therefore, there is no significant correlation between carbofuran and daphnia behavior in the model.

Week 5

H3: Death of one gammarid and alarm is attributed to tributyl phosphate

Predicted variables	chi-square (χ^2)	p-value	significance
Temperature	12.7127	0.0003	***
pH	0.1634	0.6860	
Oxygen	0.1179	0.7313	
Conductivity	1.1198	0.2899	
NH4	0.3686	0.5437	
Tributyl phosphate	0.1098	0.7403	

No, the p-value for the predictor "Tributyl phophate" in the Type II Wald chi-square test is 0.8058, which is greater than the common significance level of 0.05. Therefore, there is no significant correlation between tributyl phosphate and gammarid behavior in the model.

Chi-square (χ^2): The larger the chi-square value, the more evidence there is against the null hypothesis (i.e., the predictor has a significant effect).
p-value: The p-value indicates the probability of obtaining a chi-square statistic as extreme as the one observed, assuming the null hypothesis is true.

Significance Codes:

- '***' (0.001): Highly significant
- '**' (0.01): Very significant
- '*' (0.05): Significant
- '.' (0.1): Marginally significant
- ' ' (1): Not significant

S14 MS2Field

In this section, we provide a detailed description of the MS2Field trailer's technical specifications and analytical setup. We also outline the LC-HRMS settings and methods used during the measurement of WWTP effluent. This includes the quantification process for selected target compounds, the approach for non-target screening, and the steps involved in the identification and quantification of non-target compounds.

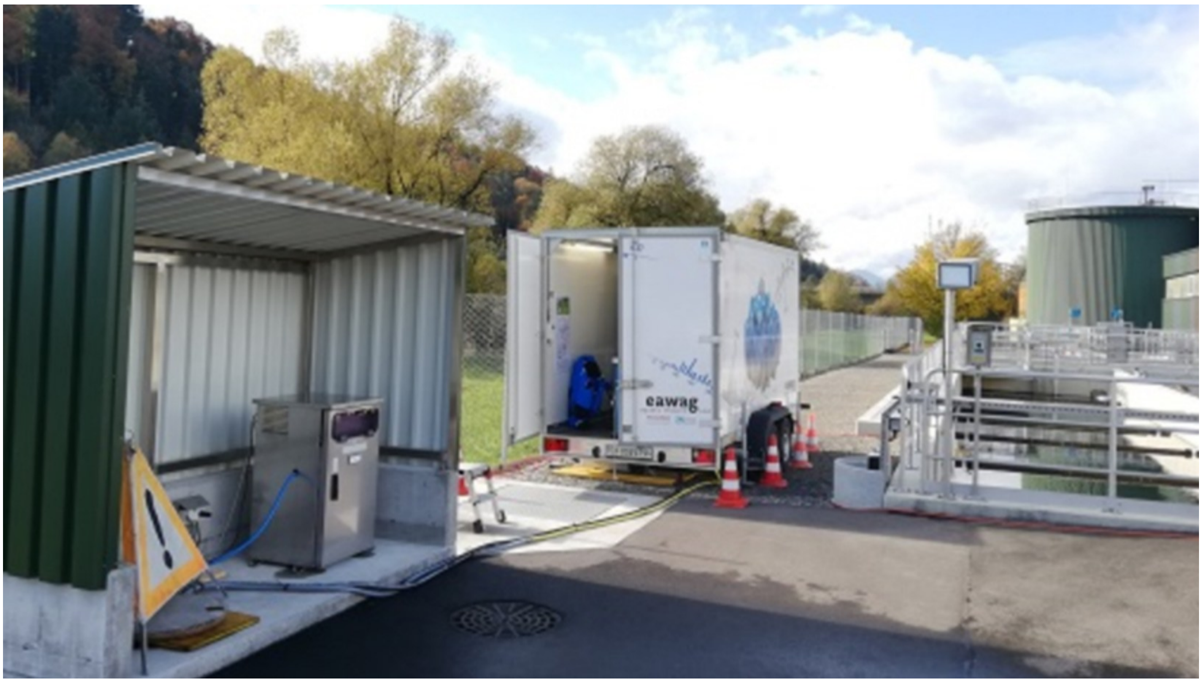

Figure S21. Setup of MS2Field at the municipal WWTP

S15 Technical Information

The MS2Field[17] is a mobile trailer with dimensions of 4 meters in length, 1.85 meters in width, and 2 meters in height (Figure S21). It is equipped with an eccentric screw pump that delivers water at a rate of 10 liters per minute through a self-cleaning filtration device (Collins 9150, 2 μm mesh stainless steel disc, TWPInc.). Water flow control at the end of the loop is facilitated by a controllable ball valve (R420+LR24A-SR/Z, Belimo). The pressure is meticulously monitored using a flow meter (Picomag DMA20-AAAAA1, Endress+Hauser) and a pressure

gauge (Cerabar PMC11, Endress+Hauser) positioned before the ball valve. Additionally, the trailer features a compact nitrogen generator with a built-in compressor (LCMS30-1, Parker Hannifin) and a dry compressing vacuum pump (ECODRY 65 plus, Leybold). Maintaining controlled temperature and humidity conditions is achieved through a split air conditioning system (MSZ-SF50VE3, Mitsubishi). The room conditions are constantly monitored using two temperature sensors (TST434, Endress+Hauser) and a smoke detector (RAUCH-8 v2, WUE). Further sensors are employed to detect water on the floor (Liquipoint T FTW31/FEW54, Endress+Hauser), temperature of the filtration device (TST602, Endress+Hauser), and nitrogen pressure on the generator (Cerabar M PMP51, degreased, Endress+Hauser). Surveillance within the trailer is ensured by two installed cameras (TV-IP420P, TRENDNET) monitoring the interior and the dilutor tool syringe (TV-IP340PI, TRENDNET). An electrical cabinet, mounted on the back wall, incorporates controllable electric plugs for all instruments and appliances. Within the cabinet, an LTE modem (Teltonika RUT-955 LTE) provides internet connectivity.

A programmable logic controller (PLC; 750-8204, WAGO) responsible for controlling the sensors, appliances, and the pump. An auxiliary computer, mounted on the cabinet (PowerBox 300-i5, Spectra), serves the purpose of monitoring surveillance cameras and acts as a data processing and backup device.

To operate the MS2Field, a level surface of approximately 5x3 meters and access to a three-phase, 400 V power supply are required. The transportation and installation process typically spans over two days. A weekly maintenance trip is necessary for treated wastewater, primarily involving tasks such as cleaning the filter, replacing SPE cartridges, cleaning the cone and ion capillary, and refilling eluents, standard, and internal standard solutions.

S16 Analytical Setup

For sample preparation and analysis procedures a programmable autosampler (PAL RTC, CTC Analytics) and a high-performance liquid chromatography (HPLC) pump (Rheos 2000, Flux Instruments) coupled to a high-resolution mass spectrometry (HRMS) instrument (Q-Exactive HF, Thermo Scientific, U.S) with ESI source was utilized. During sampling, triggered by the autosampler signal, the solenoid valve opens and increases the backpressure to initiate filtrate flow. Subsequent to sampling, the valve closes, and the ball valve opens to release pressure. At every hour, synchronized with the completion of a sampling event, the pump flow direction reverses for approximately 20 seconds to perform a "flushback" and eliminate any blockages at the sample inlet.

The six ports of the autosampler dilutor syringe are interconnected as follows:

1. Connected to the filtrate line using 0.7 mm ID, 1/16" OD PEEK tubing.
2. Linked to a reservoir containing 50 mL of internal standard solution (1000 ng/L) per compound in evian®) using 0.7 mm ID, 1/16" OD PEEK tubing.
3. Connected to a reservoir containing 50 mL of standard solution (500 ng/L per compound in Evian) using 0.7 mm ID, 1/16" OD PEEK tubing.
4. Linked to a two-port magnetic microvalve (0127-T-01,5-CC-TZ-UNFB-024/DC-03, Bürkert) that can alternate between a 5 L plastic canister containing ethanol with 5% isopropyl alcohol and a 10 L plastic canister containing evian®.
5. Connected to the 6-port valve.
6. Linked to a waste canister

The wastewater sample was collected every 20 min and was filled into a dilutor syringe that was connected to a 6-port valve. Two ports were connected to isotopically labelled internal standards (ILIS) and reference standard (STD) solutions. Then the diluter syringe was filled with a total 250 µL of the wastewater sample, 50 µL of isotope-labelled internal standard (ILIS)

(final concentration 1 μg/L) and 400 μL nanopure:Evian®water (80:20). For quality assurance, every 20th sample was either (i) spiked with 100 μL STD instead of wastewater (calibration control), (ii) with 250 μL nanopure:Evian®water (80:20) instead of wastewater (blind), (iii) with 300 μL nanopure:Evian®water (80:20) instead of wastewater and ILIS (blank), or (iv) with 100 μL STD instead of nanopure:Evian®water (spiked sample for recovery). To remove small particles, the sample was directed through a self-packed pre-column (stainless steel, 2.1 x 20 mm, BGB Analytik AG, Switzerland) containing Atlantis® T3 material (10 μm, Waters, Ireland). Then, analytes were separated on a reverse phase analytical column (Atlantis® T3 5 μm, 3.0 x 50 mm, Waters, Ireland). The sample was transferred into the LC-HRMS/MS system by large volume direct injection and was eluted at a flow rate of 300 μL/min using a gradient of ultrapure water and methanol (both acidified with 0.1 % formic acid). LC-HRMS data was acquired on a hybrid quadrupole-orbitrap mass spectrometer (Q-Exactive HF, Thermo Scientific, U.S) with an ESI source that was operated in switching mode (-3.0, 4.0 kV).

S17 LC-HRMS Method

Sampling and measurement operations are initiated and managed by the LC-HRMS data system. Each sampling and measurement cycle for one sample has a duration of 20 min, resulting in a frequency of 3 samples per hour. The first 4 min of the eluate was sent to waste to protect the mass spectrometer. Elution was achieved by an ultrapure water, methanol gradient (both acidified with 0.1% formic acid) at 300 μL/min (Figure S22).

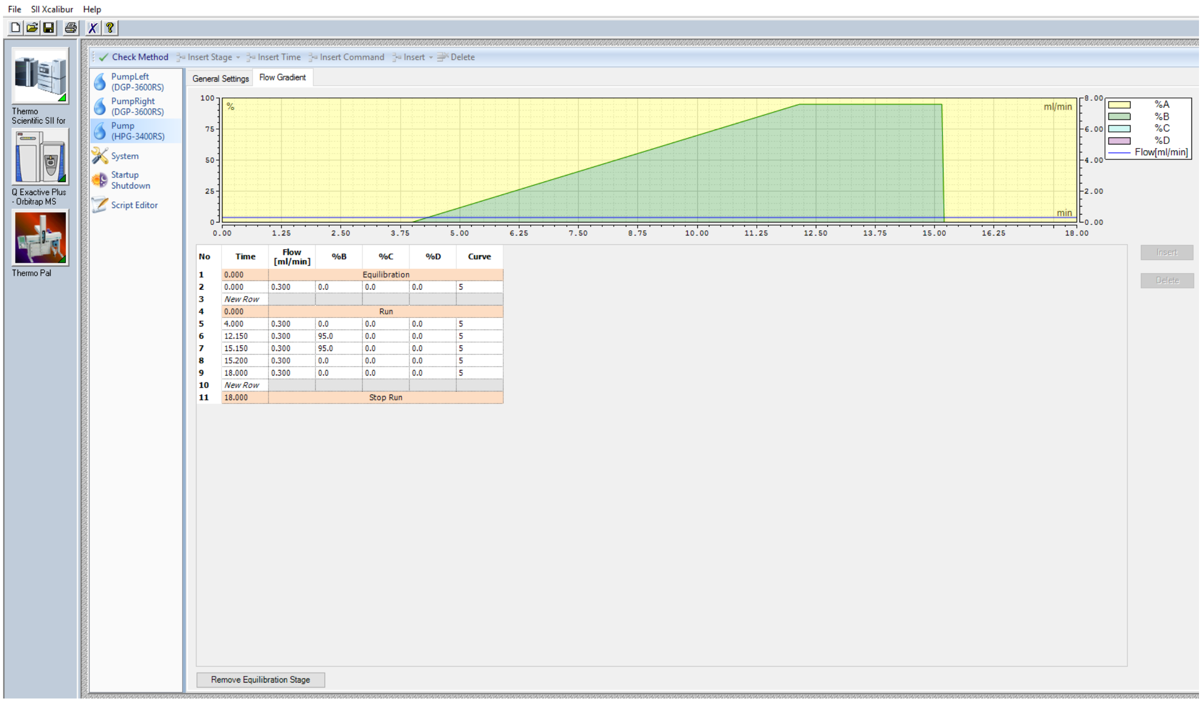

Figure S22. Gradient of HPLC-method. The first 4 min of the eluate was sent to waste to protect the mass spectrometer. Flow was kept at 300 µL/min. %B is ultrapure water with 0.1% formic acid (%) and %C is Methanol with 0.1% formic acid (%).

S18 LC-HRMS Settings

Ionization and detection in the mass spectrometer (MS) are executed using a heated electrospray source (HESI-II, Thermo Scientific). Detailed MS parameters are outlined in Table S4.

Table S4. LC-HRMS parameters

Spray voltage (kV)	4 (pos), -3 (neg)
Capillary temperature (°C)	320
Sheath gas (AU)	35
Auxiliary gas (AU)	10
Auxiliary gas temperature (°C)	40
Reserve gas	0
Probe heater temperature	40
S-lens RF level	50

The MS was operated in polarity switching mode, alternating between data-dependent and data-independent acquisition after each sample. In data-dependent acquisition, MS1 is acquired with top-2, MS2 with an inclusion list in positive and negative mode and parameters displayed in Table S5 and S6.

Table S5. Parameters for data dependent acquisition with MS1

Resolution MS1 (at m/z 200), neg/pos	120 000 / 120 000
AGC target MS/MS, neg/pos	5e5/5e5
Maximum injection time (IT) MS1 (ms), neg/pos	50/50
Scan range MS1 (m/z)	110-1000
Isolation window (m/z)	1
Number of dd-MS/MS, neg/pos	Top2/Top2
S-lens RF level (AU)	50

Table S6. Settings for data dependent acquisition with MS2

Resolution MS2 (at m/z 200), neg/pos	15 000/15 000
AGC target MS/MS, neg/pos	2e5/5e5
Maximum injection time (IT) MS2 (ms), neg/pos	32/32
Scan range MS2 (m/z)	1 m/z
Isolation window (m/z)	15, 60, 105
Number of dd-MS/MS, neg/pos	Top2/Top2
Dynamic exclusion time (s)	5

Solutions of individual reference standards were prepared as 1 g/L or 0.1 g/L in acetonitrile, ethanol, ethanol-water (50:50, v:v) or ultrapure water depending on their physicochemical properties. Reference standards prepared in the same solvent were then combined to master mixes at 10 mg/L from which dilutions were made and from which they were spiked to spike samples and calibration samples. Isotopically labelled internal standards (ILIS) were handled accordingly. Upon startup and after maintenance of the system a calibration series in evian® was prepared and measured by the LC-HRMS system at 20, 50, 100, 400, 1000 and 6000 ng/L.

S19 Quantification of Selected Targets

Data evaluation for lab samples and MS2field samples was performed using TraceFinderTM 5.1[18] (Thermo Fisher Scientific, U.S.). In the MS2field, matrix factor (MF) and relative recovery (RR) were calculated based on only one spike concentration (200 ng/L). The MF was determined by calculating the ILIS area in matrix samples relative to blank samples where a corresponding ILIS was present.

$$Matrix\ factor^{STD\ i}_{matching\ ILIS} = median(\frac{A^{ILIS\ i}_{sample\ n}}{median(A^{ILIS\ i}_{ultrapure\ samples})})\ , \qquad \text{Eq. 0.1}$$

A denotes the detected peak area for compound *i* and n the respective sample. Compounds with no matching ILIS were assigned an ILIS based on similar retention time and structural similarity. The matrix factor for these compounds was determined according to:

$$Matrix\ factor^{STD\ i}_{non-matching\ ILIS} = median(\frac{A^{STD\ i}_{spiked\ sample\ n} - A^{STD\ i}_{non-spiked\ sample\ n}}{median(A^{STD\ i}_{calibration\ samples})} \qquad \text{Eq. 0.2}$$

Limits of quantification were determined as the lowest calibration sample concentration at which a peak with at least five scans could be observed. Further, the LOQs determined in ultrapure water were corrected by the matrix factor:

$$LOQ^{STD\ i}_{Matrix} = \frac{LOQ^{STD\ i}_{ultrapure\ water}}{Matrix\ factor^{STD\ i}} \qquad \text{Eq. 0.3}$$

The relative recovery was calculated by subtracting the amount quantified in spiked samples from the amount in the preceding sample:

$$Relative\ Recovery^{STD\ i} = median(\frac{C^{STD\ i}_{spiked\ sample\ n} - C^{STD\ i}_{non-spiked\ sample\ n}}{theoretically\ spiked\ concentration})\ , \qquad \text{Eq. 0.4}$$

C denotes the raw concentration determined in TraceFinder™. However, the relative recovery was calculated only if the following condition was met:

$$C^{STD\ i}_{spiked\ sample\ n} < 1.7 * (C^{STD\ i}_{spiked\ sample\ n} - C^{STD\ i}_{non-spiked\ sample\ n}) \qquad \text{Eq. 0.5}$$

The final concentration was corrected by the relative recovery if no matching ILIS was available:

$$C_{final}^{STD\,i} = \frac{C_{raw}^{STD\,i}}{RR^{STD\,i}} \qquad \text{Eq. 0.6}$$

Table S7. Information to targets and their quantification, WWTP dataset. LOQ: limit of quantification; in water, determined as the lowest standard observed in calibration. In matrix, determined from the water LOQ divided by the matrix factor, or, if not determinable, the lowest observed value. ND (not determined) for samples where unspiked sample concentrations exceeded 500 ng/L, such that the area difference from spiking could not reliably be determined.

Compound	Lidocaine	Xylazine	4-Aminoantipyrine
Formula	C14H22N2O	C12H16N2S	C11H13N3O
CAS number	137-58-6	7361-61-7	83-15-8
Compound class	Local anesthetic	Veterinary anesthetic	Anti-inflammatory, antirheumatic drug
Ionization	[M+H]+	[M+H]+	[M+H]+
m/z	235.1805	220.1034	203.1058
Retention time (min)	8.601	8.906	7.895
LOQ (ng/L)	20	5	20
MF	0.79	0.78	0.93
RR	113	103	114
Internal Standard	Lidocaine-D10	Xylazine-D6	4-Aminoantipyrine-D3
Analytical concentration in ng/L (max)	1424	1817	4259

S20 Non-Target Screening

The raw files were converted to mzXML format using ProteoWizard MSConvert[19] and processed using an in-house workflow based on enviPick[20], open source enviMass[21] functionality and RMassScreening[22] with additional processing to improve data quality and extract fragment spectra. The complete workflow is available as a Github repository (https://github.com/meowcat/ms2field-wwtp). All specific parameters were deposited in the corresponding configuration files. Features were detected in positive and negative mode using enviPick and aligned to features across files using RMassScreening/enviMass. To reduce the

number of redundant features resulting from peak splitting (the formation of two features for the same analyte), features with very close m/z and retention time were merged into one. Features were filtered to retain only features present in non-fortified samples and absent in blanks.

Preliminary annotation of features was performed with an internal database of retention times and accurate masses using RMassScreening, and the corresponding data were extracted and visualized to verify correct processing with quantified target compounds and internal standards. Data from positive and negative modes were combined and features were grouped into components (ions belonging to the same analyte) using a procedure based on RAMClust[23] with a modification that adapts the metric for features with low sample frequency. To avoid the contribution of diurnal and other periodic matrix effects in the frequency analysis, the intensities for all features were normalized using the smoothed median of the internal standard relative log intensities for the positive and negative modes separately, and the component intensities were recalculated.

For both the normalized and the non-normalized intensity matrix of all components in the fortified samples, the Lomb periodogram[24,25] was calculated for each component and decomposed into magnitude and sine and cosine of phase. For each frequency and component, the lower of the corrected and uncorrected frequency magnitudes was retained to avoid the introduction of frequency components from the normalization procedure. From the column-wise z-scaled and centered matrix, the Euclidean distance matrix was computed and used as input for hierarchical agglomerative clustering using average linkage[26]. The dendrogram was cut into clusters using dynamic tree cutting[27].

Metadata (precursor, intensity, etc.) was extracted for all tandem mass spectra in all files acquired in data-dependent mode, and spectra were grouped into spectral groups based on precursor mass and retention time. For each spectral group, the (up to) five spectra with the

highest precursor intensity were extracted. For all clusters identified as above, all associated spectra were exported in NIST MS-Search compatible format (.msp) and in SIRIUS/CSI:FingerID input format (.ms). Spectra were exported unfiltered and, to improve the quality of potentially chimeric spectra, filtered to include only fragments whose EIC followed the parent ion EIC in the data-independent acquisition. Preliminary identification was performed utilizing spectral libraries, including mzCloud[28] and Massbank[29]. Predicted compositions were derived based on the accurate mass of ions, enabling the inference of elemental formulas for the compounds. Additionally, experimental mass spectra were compared against predefined m/z values in the MassList to aid in compound identification. Identification confidence was assigned by expert judgement according to Schymanski et al. (2014)[30]. Level 1 (authentic standard) was assigned for an MS2 spectral match if, in addition, the EIC of the precursor mass matched the authentic standard. Level 2a was assigned 6 for a good spectral match when there are no other good matches and other likely compounds (isomers) are not expected to have a very similar spectrum. Level 3 was assigned to compounds with a good spectral match to several similar compounds, or when other isomers that could be imagined to be present are expected to have a similar spectrum. The agreement of several cluster A features with bile acid compounds is rated as level 3, because the MS2 spectra of bile acids are very similar and hardly distinguishable from the agreement score alone (note that different weighting of the score could already influence the results). For homologous series in cluster E, level 3 (chemical class) was assigned if CSI:FingerID predicted structures of the polyol homolog type, or if there was an MS2 database match to a propylpolyol homolog. As MS2 spectra appear to be very similar for all polyol-based structures, level 2a was not assigned even for database matches. Level 4 (formula only) was assigned when homolog series membership was inferred from $\Delta m/z$ and ΔRT only.

S21 Identification and Quantification of Non-Targets

For the non-target screening, Compound Discoverer (V3.3) software was used and the filtration (retention time between 1 and 20 min, quality criteria for the signals, blank subtraction as well as a minimum area of equal to or greater than 1.0×10^8) resulted in a total of 600 substance profiles for the second week, 57 for the third week and 278 for the fifth week, which are discussed in the results and discussion section. The structures of the filtered substances were matched and confirmed utilizing spectral libraries, including mzCloud[28] and ChemSpider[31]. Predicted compositions were derived based on the accurate mass of ions, enabling the inference of elemental formulas for the compounds. Additionally, experimental mass spectra were compared against predefined m/z values in the MassList to aid in compound identification. Reference standards for all three compounds confirmed the identification according to Schymanski et al. (2014)[17]. The quantification of non-target compounds, including carbofuran, 2,4-dichlorphenol, and tributyl phosphate, was conducted using a QExactive Plus LCMS system (Thermo Scientific). A calibration curve was generated from known standards of Lidocaine-D10, and peak intensities of the non-target compounds in samples were compared to this curve to determine their concentrations. Data analysis was performed using TraceFinder 5.1 software. Information on compounds is shown in Table S8 and calibration curves in Figure S24.

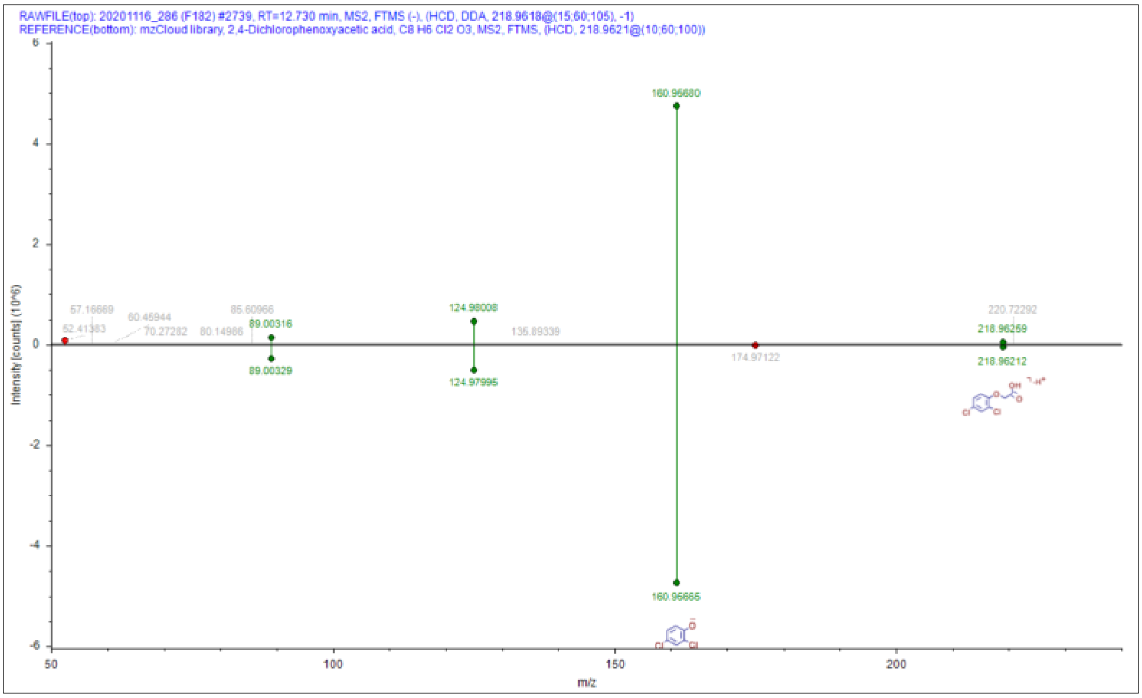
RAWFILE(top): 20201116_286 (F182) #2739, RT=12.730 min, MS2, FTMS (-), (HCD, DDA, 218.9618@(15;60;105), -1)
REFERENCE(bottom): mzCloud library, 2,4-Dichlorophenoxyacetic acid, C8 H6 Cl2 O3, MS2, FTMS, (HCD, 218.9621@(10;60;100))
160.96680
124.98008
89.00316
218.96259
89.00329
124.97995
218.96212
160.96665
Intensity [counts] (10^6)
m/z
A

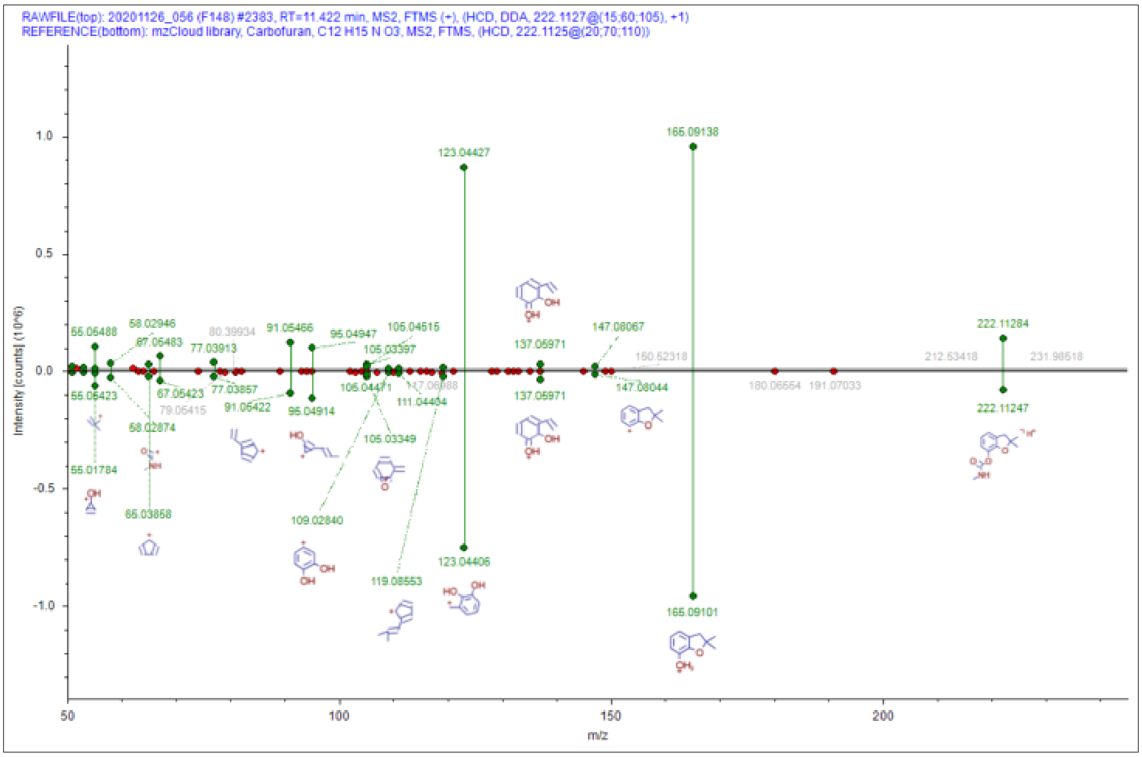
RAWFILE(top): 20201126_056 (F148) #2383, RT=11.422 min, MS2, FTMS (+), (HCD, DDA, 222.1127@(15;60;105), +1)
REFERENCE(bottom): mzCloud library, Carbofuran, C12 H15 N O3, MS2, FTMS, (HCD, 222.1125@(20;70;110))
123.04427
165.09138
222.11284
222.11247
123.04406
165.09101
Intensity [counts] (10^6)
m/z
B

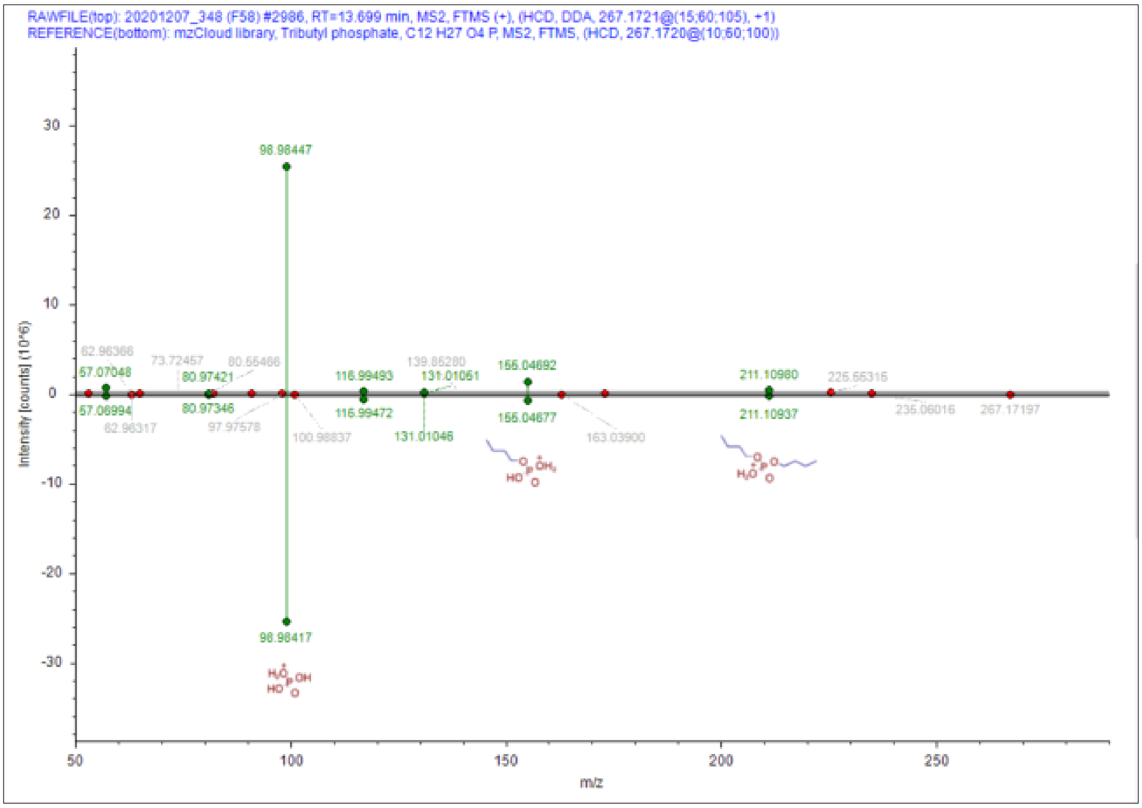

C

Figure S23. MS2 Spectra and comparison with library match in ChemSpider. A: 2,4-D. B: Carbofuran. C: Tributyl phosphate

Table S8. Analytical parameters for non-target quantification, WWTP dataset. LOQ: limit of quantification; in water, determined as the lowest standard observed in calibration. In matrix, determined from the water LOQ divided by the matrix factor, or, if not determinable, the lowest observed value.

Compound	2,4-Dichlorophenol	Carbofuran	Tributyl phosphate
Formula	C6H4Cl2O	C12H15NO3	C12H27O4P
CAS number	21563-66-2	1563-66-2	126-73-8
Compound class	Pesticide	Insecticide	Solvent
Ionization	[M+H]+	[M+H]+	[M+H]+
m/z	222.1125	160.9466	267.1720
Area (Max)	358727817	33991518	920370709
Retention time (min)	12.7	11.49	13.7
Internal Standard	Losartan-D4	Midazolam-D4	Fenofibrate-D6
Retention time ILIS (min)	12.17	10.23	14.15
Analytical concentration in ng/L (max)	1040200	1462	15333
Calibration points	4	5	5

A
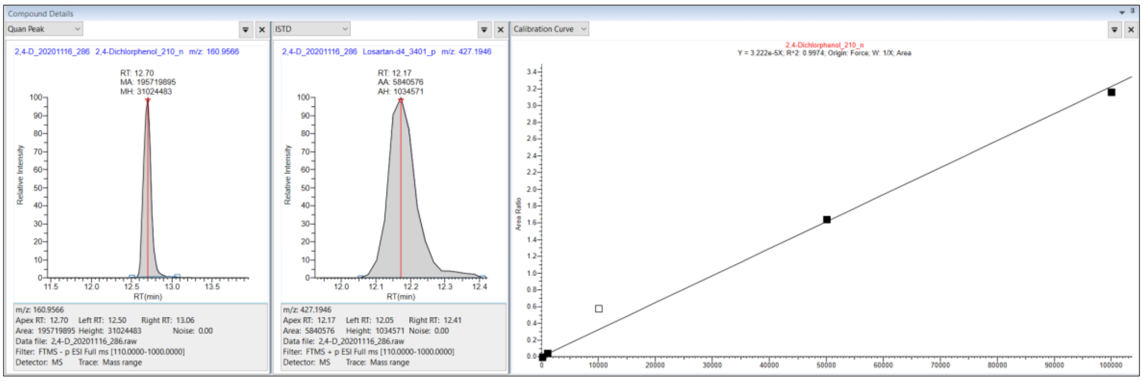

B
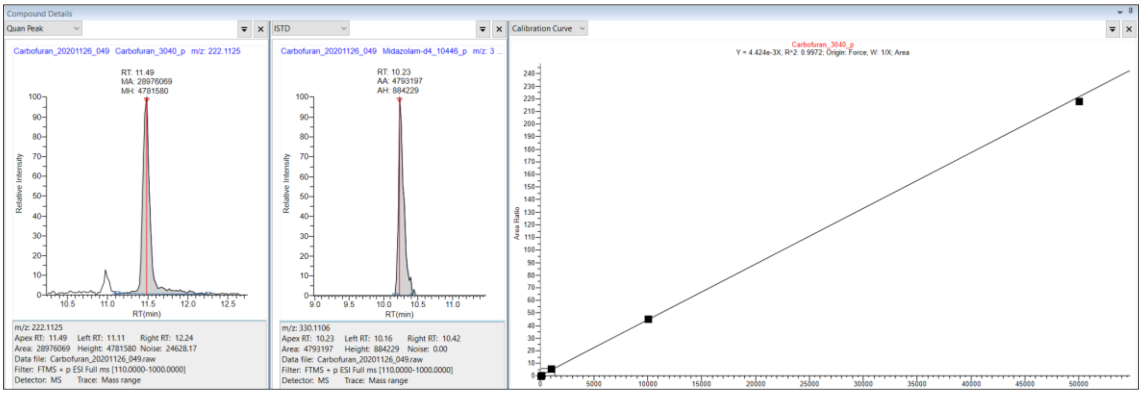

C
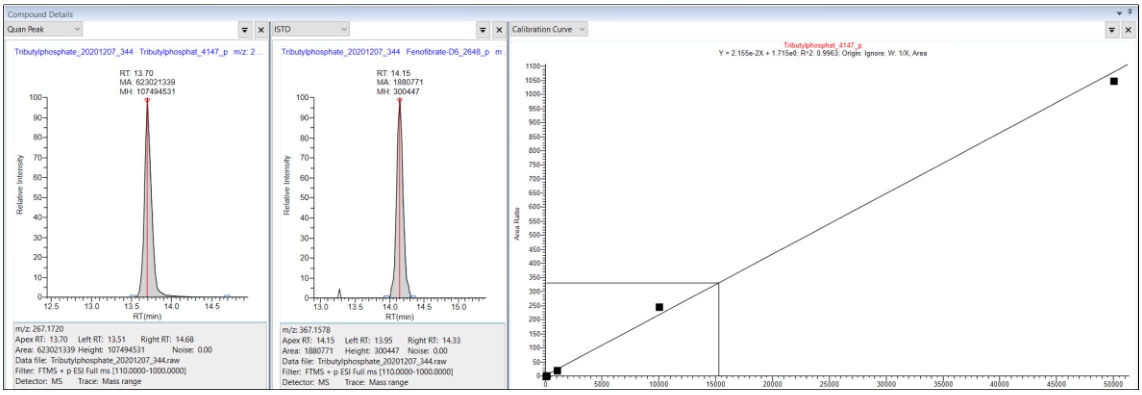

Figure S24. Chromatograms of quan peak, ILIS and calibration curve of quantified non-targets. A: 2,4-D. B: Carbofuran. C: TBP

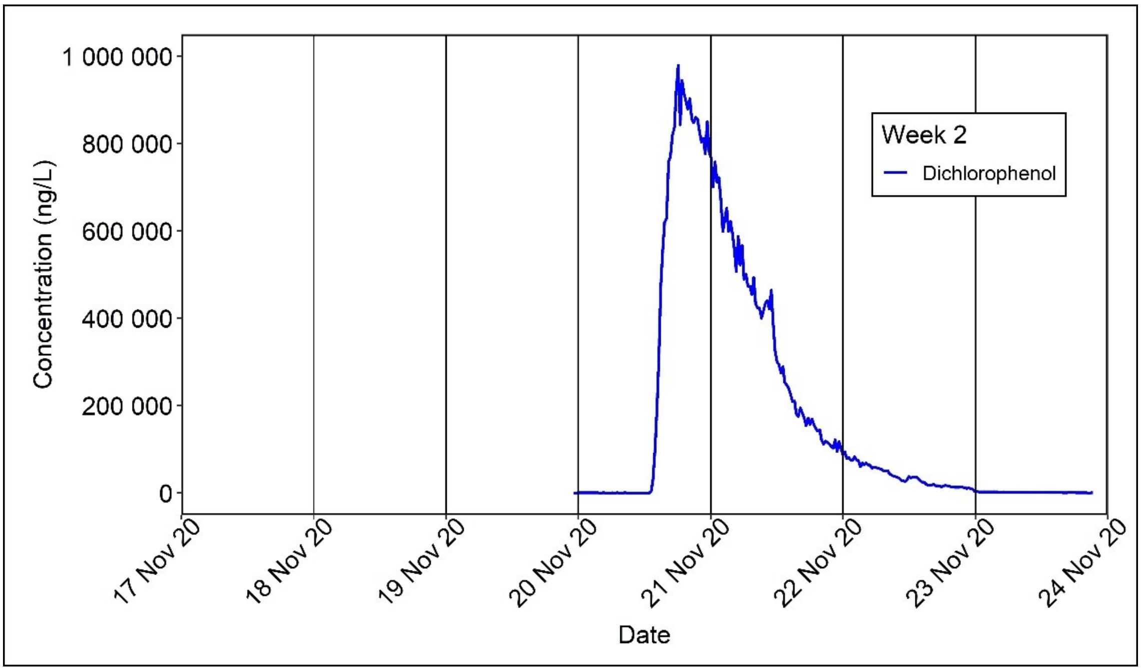

Week 2
Dichlorophenol
Concentration (ng/L)
1 000 000
800 000
600 000
400 000
200 000
0
17 Nov 20
18 Nov 20
19 Nov 20
20 Nov 20
21 Nov 20
22 Nov 20
23 Nov 20
24 Nov 20
Date

A

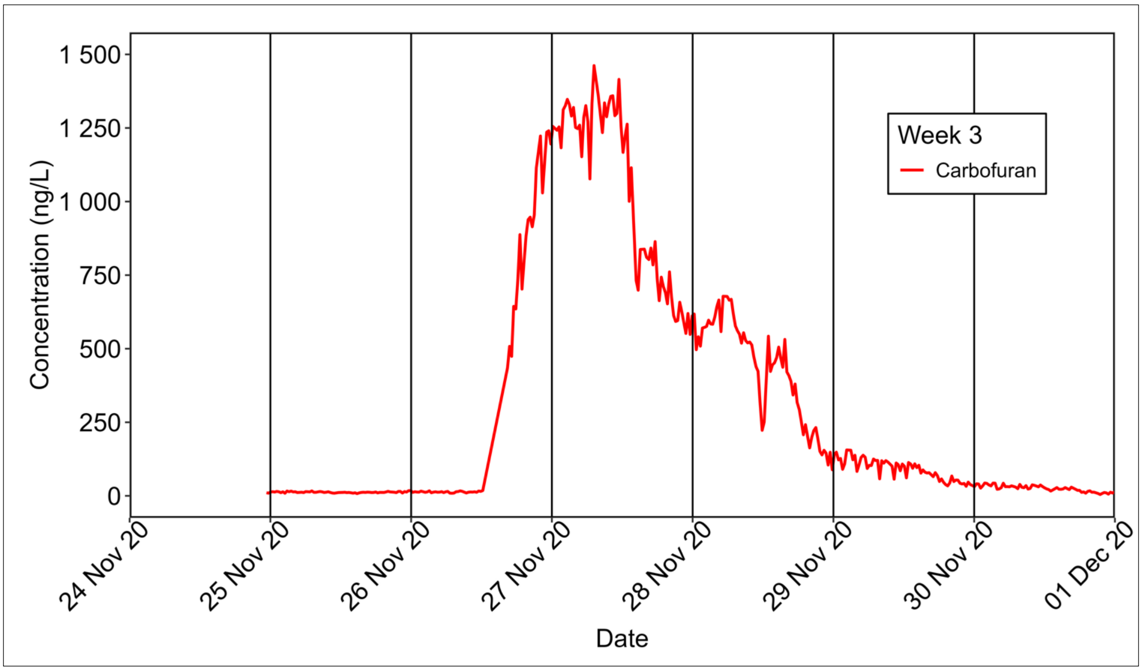

Week 3
Carbofuran
Concentration (ng/L)
1 500
1 250
1 000
750
500
250
0
24 Nov 20
25 Nov 20
26 Nov 20
27 Nov 20
28 Nov 20
29 Nov 20
30 Nov 20
01 Dec 20
Date

B

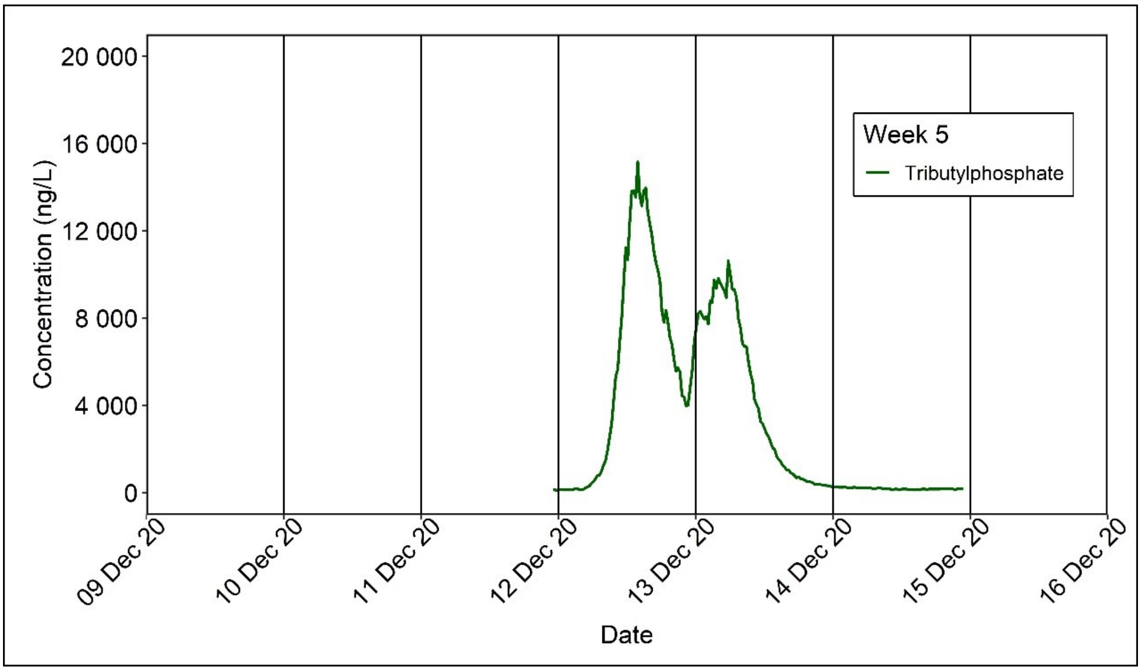

C

Figure S25. Concentration trends of quantified non-targets. A: 2,4-D. B: Carbofuran. C: Tributyl phosphate.

S22 Preparation and Analysis of Laboratory Experiment Samples

S23 Sample Preparation for Laboratory Experiments

Table S9. Information on spike substance, preparation for exposure tests, sampling and analysis

General Information	
Sample type	Spiked wastewater samples of verification tests
Substance	Carbofuran PESTANAL analytical standard
Brand	Sigma-Aldrich
CAS Number	1563-66-2
Formula	$C_{12}H_{15}NO_3$
PURITY (HPLC AREA %)	98.0 % ≤
Wastewater preparation	
Volume	100 L + 20 L of Sensaguard container
Filtration	0.08 µm
Type	effluent
Treatments	biological
Temperature	16 ± 1 °C
Spike preparation (exemplary) → See Excel-SI Table X1 for all calculations	

Compound	Carbofuran
Mass	5.4 mg
Sample Volume	0.5 mL
1. Concentration	10.8 mg/L
Dilution	1:40 (12.5 mL in 487.5 mL)
Sample Volume	0.5 L
2. Concentration	0.27 mg/L
Medium	Nanopure water
Solvent	No
End volume	119.5 L filtrated wastewater effluent + 0.5 mL spike solution
End concentration	0.0022 mg/L
Sampling for chemical analysis	
Exposure	20 h
1. Sampling	after 1 h (pre-exposure)
2. Sampling	after 20 h (post-exposure)
Volume	50 mL
Storage	in dark, at -20°C
Vessel	Schott flask
Sample preparation procedure for analysis	
1. Thaw in a warm water bath (20 °C)	
2. Transfer 1 mL of the sample into a microcentrifuge tube (MS tube)	
3. Centrifuge the sample for 10 min at room temperature, at 5000 rpm	
4. Transfer 600 µL of the supernatant into a new microcentrifuge vial (MS vial)	
5. Spike the sample with 10 µL of Internal Standard (ISTD) to achieve a final concentration of 1000 ng/L.	
6. Spike the sample with 50 µL of ethanol	
7. Shake the vial to ensure homogeneity	
Spike analysis → See Excel-SI Table X2 for all analytical concentrations	

S24 Quantification of Spike Samples of Laboratory Experiments

Chemical analysis of carbofuran was performed on an Orbitrap Fusion Lumos (Thermo Scientific) Mass Spectrometer coupled to an Ultra High Performance Liquid Chromatograph (UHPLC-System) for chromatographic separation. The external standard calibration with structurally identical deuterated analytes (i.e., adding the IS mixture to the sample directly before LC-MS/MS analysis) and interpolation using a linear regression model were performed to determine the concentrations of the individual compounds by Tracefinder 5.1 (Thermo

Scientific). a calibration series in evian® was prepared and measured by the LC-HRMS system at 10, 25, 50, 100, 250, 500, 1000, 2500, 5000 and 10000 ng/L. The calibration curve is shown in Figure S26.

The target substance was quantified based on the area ratio of the analyte and its structurally identical isotope-labelled internal standard. The extracted signals were verified as target substance by comparing the retention time (RT), the MS/MS spectra and the isotopic patterns to those of the corresponding reference standard. Limit of detection (LOD) as a signal-to-noise (S/N) ratio of 3:1 and and at least five data points. Analytical concentrations are listed in table X2 in Excel-SI.

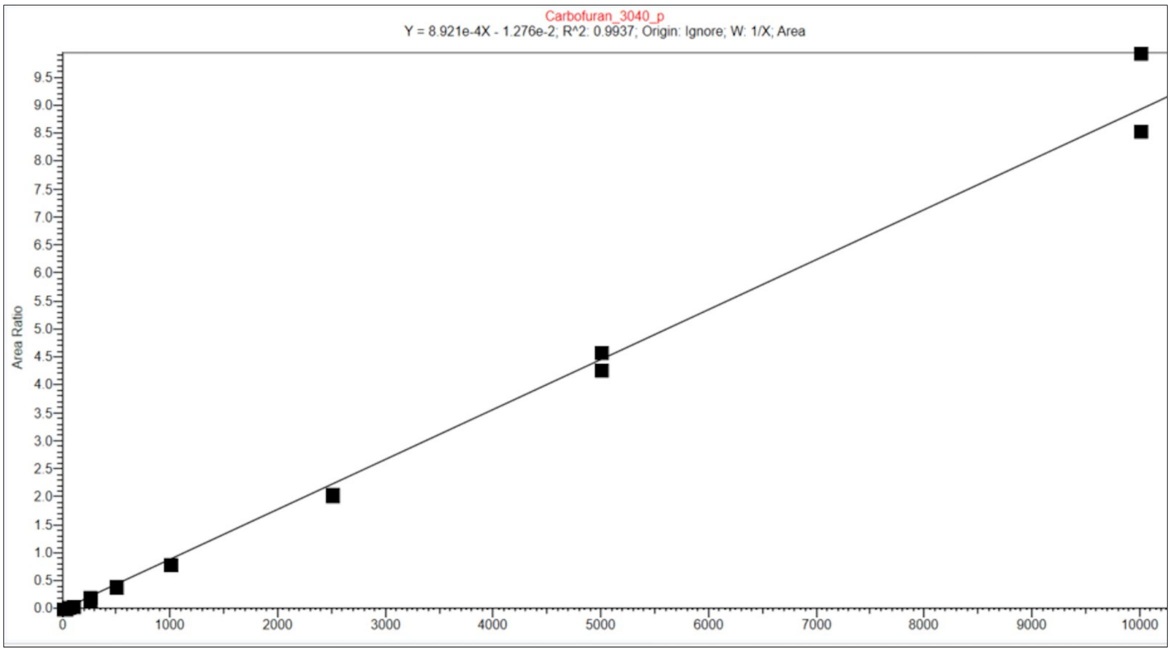

Figure S26. Calibration curve of carbofuran for analytical concentrations of laboratory experiments.

S25 Physicochemical Parameters

Detailed information about the measured physicochemical parameters (pH, oxygen and conductivity) and general water chemistry (ammonia, nitrite and nitrate) of the effluent of the pilot WWTP during the verification test with carbofuran and the effluent of the municipal WWTP during the monitoring can be found in Excel-SI (Table X3-5).

S26 Space and Time Requirements for Online Monitoring Tools

Table S10. Space and time per week to maintain and operate BEWS and MS2Field in WWTPs provides a comparative overview of the time and space requirements associated with operating three different BEWS and MS2Field in the WWTPs.

Table S10. Space and time per week to maintain and operate BEWS and MS2Field in WWTPs

System	Cultivation/ Sampling (h)	Maintenance (h)	Infrastructure
**Algae Toximeter**	1	2	Indoor space of 10 m^2 with access to treated wastewater, clean water, electricity and internet
**DaphTox II**	1.5	1.5	
**Sensaguard**	2	1	
**MS2Field**	-	2-3	Flat utility space with access to treated wastewater, electricity and internet
Additional costs including travel time to the WWTP, the initial installation and the purchase or rental fees of the systems are excluded			

References

[1] Bundschuh, M., Zubrod, J. P., Seitz, F., Stang, C., & Schulz, R. (2011). Ecotoxicological evaluation of three tertiary wastewater treatment techniques via meta-analysis and feeding bioassays using Gammarus fossarum. *Journal of Hazardous Materials*, *192*(2), 772-778. https://doi.org/10.1016/j.jhazmat.2011.05.079

[2] Herrmann, H., Töfke, S., & Baier, B. (2009). Untersuchungen zur Praxistauglichkeit eines modifizierten Algentoximeters.

[3] bbe Fundamental Manual for the Algae Toximeter. BBE Moldaenke, Preetzer Chaussee 177, D -24222 Schwentinental. Version 3.2 E1, 09/08

[4] Lechelt, M., Blohm, W., Kirschneit, B., Pfeiffer, M., Gresens, E., Liley, J., Holz, R., Lüring, C. & Moldaenke, C. (2000). Monitoring of surface water by ultrasensitive Daphnia toximeter. *Environmental Toxicology: An International Journal*, *15*(5), 390-400. https://doi.org/10.1002/1522-7278

[5] Hinkley, D. V. (1970). Inference about the change-point in a sequence of random variables. Biometrika, 57(1), 1-17.

[6] bbe Fundamental Manual for the DaphtoxII - Annotations and explanations to fundamental and theoretical aspects of toxic effects to Daphnia magna. BBE Moldaenke, Preetzer Chaussee 177, D -24222 Schwentinental. Version 3.5 E2, 10/2012)

[7] Lopez-Mangas, A. Singer, H., Langer, M., (2024, unpublished). Simultaneous online chemical and behavior measurements in the field indicate that pesticide peaks alter gammarid behavior patterns.

[8] Weinrich, L., & Grélot, A. (2008). Evaluation of innovative operation concept for fiat sheet MBR filtration system. *Water Science and Technology*, *57*(4), 613–620. https://doi.org/10.2166/wst.2008.124

[9] Lin, K. (2009). Joint acute toxicity of tributyl phosphate and triphenyl phosphate to Daphnia magna. Environmental Chemistry Letters, 7(4), 309-312. https://doi.org/10.1007/s10311-008-0170-1

[10] ABC Laboratories (199. Acute flow-through toxicity exposure of TBP to *Gammarus pseudolimnaeus*. ABC Study No. 39499. Test conducted at the request of the Synthetic Organic Chemical Manufacturers Association, Inc. Columbia (MO): ABC Laboratories.

[11] R. R Foundation for Statistical Computing, **2024**. R version 4.4.1. https://www.R-project.org/ (accessed 2024-07-15).

[12] Pickering, B. J. (2019). smop: Subset Multivariate Optimal Partitioning. R package version 0.1.1. https://eprints.lancs.ac.uk/id/eprint/81171/1/2016PickeringPhd.pdf

[13] Pickering, B. J. (2015). Changepoint Detection for Acoustic Sensing Signals. December, 1–181. Retrieved from https://eprints.lancs.ac.uk/id/eprint/81171/1/2016PickeringPhd.pdf

[14] Lenth, R. V. (2022). emmeans: Estimated Marginal Means, aka Least-Squares Means. R package version 1.8.0. Available at: https://CRAN.R-project.org/package=emmeans

[15] Hothorn, T., Bretz, F., & Westfall, P. (2008). Simultaneous inference in general parametric models. Biometrical Journal, 50(3), 346-363. doi:10.1002/bimj.200810425.

[16] Brooks, M. E., Kristensen, K., van Benthem, K. J., Magnusson, A., Berg, C. W., Nielsen, A., Skaug, H. J., Maechler, M., & Bolker, B. M. (2017). glmmTMB balances speed and flexibility among packages for zero-inflated generalized linear mixed modeling. The R Journal, 9(2), 378–400.

[17] Stravs, M. A., Stamm, C., Ort, C., & Singer, H. (2021). Transportable automated HRMS platform "MS2Field" enables insights into water-quality dynamics in real time. Environmental Science & Technology Letters, 8(5), 373-380. https://doi.org/10.1021/acs.estlett.1c00066

[18] TraceFinder 5.1. Thermo Fisher Scientific, **2023**. Retrieved from https://www.thermofisher.com/ (accessed 2024-07-15).

[19] Chambers, M. C., Maclean, B., Burke, R., Amodei, D., Ruderman, D. L., Neumann, S., Gatto, L., Fischer, B., Pratt, B., Egertson, J., Hoff, K., Kessner, D., Tasman, N., Shulman, N., Frewen, B., Baker, T. A., Brusniak, M. Y., Paulse, C., Creasy, D., … Mallick, P. (2012). A cross-platform toolkit for mass spectrometry and proteomics. Nature Biotechnology, 30(10), 918-920. doi:10.1038/nbt.2377

[20] Loos, M., & Singer, H. P. (2017). Exploring the identification of microcontaminants in lake water by combining passive sampling with high resolution mass spectrometry and a case-control approach. Environmental Science & Technology, 51(11), 5972–5980. doi:10.1021/acs.est.6b05733

[20] Loos, M. (2020). enviPick: peak picking for liquid chromatography–high-resolution mass spectrometry data in non-targeted environmental analysis. Journal of Cheminformatics, 12(1), 1-12. doi:10.1186/s13321-020-00456-2

[21] Loos, M., & Singer, H. P. (2017). Exploring the identification of microcontaminants in lake water by combining passive sampling with high resolution mass spectrometry and a case-control approach. Environmental Science & Technology, 51(11), 5972–5980. doi:10.1021/acs.est.6b05733

[22] Stravs, M. (2022). RMassScreening: Screening workflow for environmental analysis using high-resolution mass spectrometry data. GitHub repository. Available at: https://github.com/meowcat/RMassScreening

[23] Broeckling, C. D.; Afsar, F. A.; Neumann, S.; Ben-Hur, A.; Prenni, J. E. RAMClust: A Novel Feature Clustering Method Enables Spectral-Matching-Based Annotation for Metabolomics Data. Analytical chemistry 2014, 86 (14), 6812–6817. https://doi.org/10.1021/ac501530d.

[24] Lomb, N. R. Least-Squares Frequency Analysis of Unequally Spaced Data. Astrophys Space Sci 1976, 39 (2), 447–462. https://doi.org/10.1007/BF00648343.

[25] Scargle, J. D. Studies in Astronomical Time Series Analysis. II - Statistical Aspects of Spectral Analysis of Unevenly Spaced Data. The Astrophysical Journal 1982, 263, 835–853. https://doi.org/10.1086/160554.

[26] Sokal, R. R.; Michener, C. D. A Statistical Method for Evaluating Systematic Relationships. The University of Kansas Science Bulletin 1958, 38 (2), 1409–1438.

[27] Langfelder, P.; Zhang, B.; Horvath, S. Defining Clusters from a Hierarchical Cluster Tree: The Dynamic Tree Cut Package for R. Bioinformatics 2008, 24 (5), 719–720. https://doi.org/10.1093/bioinformatics/btm563.

[28] MZCloud. HighChem LLC, Slovakia, **2024**. https://www.mzcloud.org/ (accessed 2024-07-15).

[29] MassBank. MassBank Project, **2011**. https://massbank.eu/ (accessed 2024-07-15).

[30] Schymanski, E. L., Singer, H. P., Longrée, P., Loos, M., Ruff, M., Stravs, M. A., ... & Hollender, J. (2014). Strategies to characterize polar organic contamination in wastewater: exploring the capability of high-resolution mass spectrometry. *Environmental science & technology*, *48*(3), 1811-1818. https://doi.org/10.1021/es4044374

[31] Chemspider. Royal Society of Chemistry, **2024**. https://www.chemspider.com/ (accessed 2024-07-15).
